# Risk of hospital admission with covid-19 among teachers compared with healthcare workers and other adults of working age in Scotland, March 2020 to July 2021: population based case-control study

**DOI:** 10.1136/bmj.n2060

**Published:** 2021-09-02

**Authors:** Lynda Fenton, Ciara Gribben, David Caldwell, Sam Colville, Jen Bishop, Martin Reid, Jane White, Marion Campbell, Sharon Hutchinson, Chris Robertson, Helen M Colhoun, Rachael Wood, Paul M McKeigue, David A McAllister

**Affiliations:** 1 Public Health Scotland, Glasgow, G2 6QE, UK; 2 Department of Statistics, Glasgow Caledonian University, Glasgow, UK; 3 Department of Mathematics and Statistics, University of Strathclyde, Glasgow, UK; 4 Usher Institute, University of Edinburgh, Edinburgh, UK; 5 Institute of Health and Wellbeing, University of Glasgow, Glasgow, UK

## Abstract

**Objective:**

To determine the risk of hospital admission with covid-19 and severe covid-19 among teachers and their household members, overall and compared with healthcare workers and adults of working age in the general population.

**Design:**

Population based nested case-control study.

**Setting:**

Scotland, March 2020 to July 2021, during defined periods of school closures and full openings in response to covid-19.

**Participants:**

All cases of covid-19 in adults aged 21 to 65 (n=132 420) and a random sample of controls matched on age, sex, and general practice (n=1 306 566). Adults were identified as actively teaching in a Scottish school by the General Teaching Council for Scotland, and their household members were identified through the unique property reference number. The comparator groups were adults identified as healthcare workers in Scotland, their household members, and the remaining general population of working age.

**Main outcome measures:**

The primary outcome was hospital admission with covid-19, defined as having a positive test result for SARS-CoV-2 during hospital admission, being admitted to hospital within 28 days of a positive test result, or receiving a diagnosis of covid-19 on discharge from hospital. Severe covid-19 was defined as being admitted to intensive care or dying within 28 days of a positive test result or assigned covid-19 as a cause of death.

**Results:**

Most teachers were young (mean age 42), were women (80%), and had no comorbidities (84%). The risk (cumulative incidence) of hospital admission with covid-19 was <1% for all adults of working age in the general population. Over the study period, in conditional logistic regression models adjusted for age, sex, general practice, race/ethnicity, deprivation, number of comorbidities, and number of adults in the household, teachers showed a lower risk of hospital admission with covid-19 (rate ratio 0.77, 95% confidence interval 0.64 to 0.92) and of severe covid-19 (0.56, 0.33 to 0.97) than the general population. In the first period when schools in Scotland reopened, in autumn 2020, the rate ratio for hospital admission in teachers was 1.20 (0.89 to 1.61) and for severe covid-19 was 0.45 (0.13 to 1.55). The corresponding findings for household members of teachers were 0.91 (0.67 to 1.23) and 0.73 (0.37 to 1.44), and for patient facing healthcare workers were 2.08 (1.73 to 2.50) and 2.26 (1.43 to 3.59). Similar risks were seen for teachers in the second period, when schools reopened in summer 2021. These values were higher than those seen in spring/summer 2020, when schools were mostly closed.

**Conclusion:**

Compared with adults of working age who are otherwise similar, teachers and their household members were not found to be at increased risk of hospital admission with covid-19 and were found to be at lower risk of severe covid-19. These findings should reassure those who are engaged in face-to-face teaching.

## Introduction

School closures have formed part of the response to the covid-19 pandemic in most countries. Although the duration and extent of closures have varied, children and young people across many countries and regions have had limited access to schools throughout the pandemic.^1^ Such limited access has been found to reduce educational opportunities, limit social interactions, and harm physical and mental health, particularly among children from socioeconomically deprived backgrounds.^2^

Worldwide, governments have been required to weigh the risks and benefits of school closures. Among the complex considerations is whether providing education in person poses an increased risk to teachers, which has also been a concern for teachers’ representatives. Studies of this risk have been limited by small numbers of events, selection biases, a lack of data on potentially important potential confounders, such as the prevalence of underlying conditions, and too narrow a focus on specific settings resulting in findings of uncertain wider applicability.^3^ ^4^ ^5^ ^6^ ^7^

Using the well established informatics infrastructure for covid-19 health records in Scotland,^8^ ^9^ ^10^ we examined the risk of covid-19 in teachers in Scotland. Scottish schools closed during the first wave of the pandemic but were fully open with in-person teaching from August to December 2020. At that time class sizes were unchanged from those before the pandemic, and physical distancing was not required between primary school pupils but was recommended between staff and pupils and encouraged among secondary school pupils when possible. Primary school pupils were not required to wear masks, and pupils in secondary schools were initially only required to wear masks in communal, non-classroom areas, and then from November 2020 in classrooms in areas with high case rates, and from March 2021 mask wearing was required at all times in school.^11^ In October 2020 a second wave of covid-19 occurred, with overall population rates of infection reaching a seven day case rate of around 150 per 100 000 in early November. Antibody testing at the end of October 2020 indicated that just over 7% of the adult population had antibodies to SARS-CoV-2; and similar levels were observed among staff in educational settings.^12^ ^13^ A further wave of infections associated with the alpha variant prompted further school closures in January 2021, with subsequent phased reopening. In May and June 2021, the delta variant spread in Scotland. During this time, Scotland had some of the highest rates of covid-19 in Europe, and the covid-19 vaccination programme was underway, with many adults of working age having been offered a first dose. At this time, schools were fully open.

This combination of circumstances provided us with an opportunity to estimate the risk of covid-19 among teachers in Scotland throughout the whole academic year, and during two separate periods of full in-person teaching when community transmission of SARS-CoV-2 was substantial. Before obtaining data on exposures, we prespecified hospital admission with covid-19 as the primary outcome. We chose hospital admission rather than focusing on any case of covid-19 or severe covid-19, as we judged that cases of covid-19 were highly susceptible to ascertainment bias (affected by both individual behaviour for testing and individual access to testing) and number of events of severe covid-19 were likely to be too small in populations of working age. We estimated the relative risks in summer 2020 and winter 2020/2021 while schools were closed, in spring 2021 during a period of phased reopening, and in autumn 2020 and summer 2021 when schools were fully open.

## Methods

We linked datasets of all the teachers to an existing case-control dataset that contains information on covid-19 cases in Scotland and matched population controls. The advantage of linking to an existing case-control study was that we could leverage the extensive data processing and cleaning (especially of covariate data) that we had already performed to produce results more rapidly. The case-control study uses incidence density sampling such that the effect estimates calculated using these data are identical to hazard ratios obtained from an equivalent whole population cohort study analysed using Cox proportional hazards models.^14^

As a result of previous work, the existing case-control study includes information on whether participants are healthcare workers.^10^ This enabled us to compare not only rates of covid-19 in teachers with rates in the general population but also teachers compared with a known high risk group (patient facing healthcare workers) and with an occupational group not expected to be at increased risk (non-patient facing healthcare workers). For patient facing healthcare workers we used our previous definitions.^10^ For non-patient facing healthcare workers we applied a stricter definition, including only those staff most likely to be working in non-clinical settings (finance, human resources, information technology, and call centre work) or based in National Health Service organisations not directly involved in patient care (eg, Public Health Scotland). When the patient facing status of healthcare workers was uncertain, we removed them from the case-control dataset.

The complete case data from the case-control study was also used alongside denominator data (not linked to covariates), which included all teachers and healthcare workers (and by subtraction from the population mid-year estimates, adults in neither category) to allow us to estimate absolute risks in all three groups.

### Case-control study

Public Health Scotland maintains a nested case-control study sampled from population-wide healthcare utilisation databases held by the organisation. This study, described in detail elsewhere,^9^ includes all people in Scotland who are classified as cases of covid-19, and for each case 10 controls randomly selected from the Scottish population who are of the same age (in single years) and sex, and are registered at the same general practice as the case, but who did not (on or before that date) meet the case definition. The case-control study is regularly updated, with the most recent update on 28 June 2021. Controls were ascertained using the Community Health Index database, which contains the unique healthcare identifier, other identifiers, age, sex, and general practice for people in the total population of Scotland. For the entire analysis, only adults of working age (21 to 65 years) were included. The case-control dataset is linked to recent hospital admission and prescribing data to identify underlying diseases, and to contemporaneous hospital admission and intensive care data to characterise the severity of each case. Ten controls were matched to each case.^15^

### Outcomes

All events from the onset of the pandemic until 30 June 2021 were included in the analysis. We included a further 28 days of follow-up to determine whether events on 30 June resulted in hospital admission, admission to intensive care, or death.

As in previous analyses, we defined cases of covid-19 as people with a positive reverse transcription polymerase chain reaction (RT-PCR) test result for SARS-CoV-2, or a hospital discharge with a diagnosis of covid-19 regardless of testing positive, or any death with covid-19 included as a cause (regardless of whether it was recorded as the underlying cause and regardless of any previous test result).

The primary outcome was prespecified as hospital admission with covid-19, defined as anyone with a positive test result for SARS-CoV-2 while in hospital, being admitted to hospital within 28 days of a positive test result, or a diagnosis of covid-19 noted on a hospital discharge letter.

The number of teachers and healthcare workers with severe covid-19 was anticipated to be low because this outcome is rare among adults of working age and the number of teachers and healthcare workers is relatively small. As such, despite being more robust to clinical decision making than hospital admission, severe covid-19 was selected as a secondary outcome. Severe covid-19 was defined as covid-19 resulting in death or admission to intensive care within 28 days of a positive test result.

### Occupational status

The General Teaching Council for Scotland (GTCS) holds data on every teacher in Scotland, including name, sex, date of birth, home postcode, work sector (nursery, primary school, or secondary school), last known employer, and registration number. Teachers are prompted to update their registration details annually. Teachers were defined as those of working age registered with the GTCS and currently working, or believed to be currently working, in a Scottish school. The GTCS indicated those who were teaching in February 2020 or November 2020, or both (see supplementary methods for additional details). These data were linked to the case-control study using name, sex, date of birth, and home postcode. Healthcare workers were identified using the General Practitioner Contractor Database and Scottish Workforce Information Standard System databases, as described previously.^9^

We compared outcomes in teachers with outcomes in patient facing healthcare workers, non-patient facing healthcare workers, and adults of working age who were neither teachers nor healthcare workers (the general population comparator).

Schools in Scotland reopened on or shortly after 12 August 2020. A five day lag period was included to allow for the time between exposure to SARS-CoV-2 and a positive test result. We present the results for two periods when schools were closed in spring/summer 2020 (1 March to 24 August 2020) and winter 2021 (24 December to 26 February 2021); one period of phased reopening in winter/spring 2021; and two periods when schools were fully open (25 August to 23rd December and 24 April to 30 June 2021). Household members of healthcare workers and teachers were identified through the unique property reference number, which was added to the national general practice registration database register in 2020.

### Covariates

We obtained data on age, sex, and Scottish index of multiple deprivation (an area based measure of socioeconomic deprivation) from the national general practice registration database, race/ethnicity through self-report from a range of healthcare utilisation databases (Scottish Morbidity Records (SMR) 01, 02, and 04), and comorbidity from previous hospital admission (SMR01) and drug dispensing (Prescribing Information System) data using the same definitions developed previously.^9^ ^10^ Additionally, we used the unique property reference number to obtain the numbers of adults (≥18 years) in the households of cases and controls. Vaccine status was obtained from the Scottish vaccine database.

### Statistical analysis

Summary statistics for personal, socioeconomic, and clinical characteristics were calculated for teachers, healthcare workers, and the remaining population of adults of working age. The control arm of the case-control study is effectively a stratified random sample from the entire Scottish population, where the strata are defined by the age and sex of individuals and the general practice within which they are registered. As such, if the probability of inclusion is known, the control arm can be used to obtain valid summary statistics for the whole population; this is analogous to the reweighting used when analysing survey data. To estimate the inclusion probabilities, we obtained counts of the Scottish population stratified by age (in single years), sex, Scottish index of multiple deprivation, and health board area,^16^ and for the same strata produced counts for the control arm of the case-control study. The inclusion probability was then calculated as the number of controls in each stratum divided by the total population in that stratum. We then produced statistics for the whole of Scotland (and plots of vaccination over time) for all teachers, healthcare workers, and the remaining population of adults of working age using the TableOne package in R, which allows the estimation of summary statistics in the presence of stratified sampling (including counts, proportions, means, and standard deviations) through inverse probability weighting.^17^

We produced cumulative incidence (risk) plots for hospital admission with covid-19 for all four groups (patient facing or non-patient facing healthcare workers, teachers, and adults of working age in the general population), stratified by age and sex. All events for Scotland were obtained through the case arm of the case-control study, with the denominators obtained directly for teachers and healthcare workers, and through subtraction from the mid-year estimates for the remaining population of adults of working age.

For all covid-19 outcomes (any covid-19, hospital admission, and severe covid-19), we fitted unadjusted conditional logistic regression models. These effect estimates can be interpreted as rate ratios. Unadjusted models were conditional on the matching variables (age, sex, and general practice). In the adjusted models, we additionally included terms for potential confounders such as Scottish index of multiple deprivation, race/ethnicity, number of comorbidities, and whether the individual shared a household with a healthcare worker. Because the dataset was large, to reduce computational time we restricted the conditional logistic regression models to strata including at least one teacher or one healthcare worker or one member of their household, as strata without such individuals will not contribute to effect estimates for those variables. In the main analysis we included individuals who had received a vaccine dose as this allowed us to examine the risk of hospital admission with covid-19 in teachers in relation to both their access to and their uptake of vaccination. In exploratory analyses, we censored events (and person time) occurring after vaccination. For the purposes of this analysis, we considered individuals to be post first dose from 14 days after the date of their injection. The standard error for the difference in rate ratios was calculated as the square root of the sum of the individual standard errors for each rate ratio squared. The supplementary file shows the prespecified statistical analysis plan. The analysis code is available at https://github.com/dmcalli2/tchr.

### Patient and public involvement

The constraints on time and resources of responding to the covid-19 pandemic for both public health and teaching workforces meant that a formal process of public involvement was not feasible within the timescales of this research. However, this work has been informed from inception by dialogue with representatives of teaching professionals and those working in education policy.

This research was prompted by concern expressed by teaching professionals in Scotland about their potential occupational risk, in discussions before the return to in-person teaching in August 2020. This was communicated through the engagement of professional associations in policy forums, in particular the COVID Education Recovery Group (CERG, www.gov.scot/groups/covid-19-education-recovery-group/). This study design was proposed as part of a programme of enhanced surveillance for education discussed in July 2020 with the range of education partners on CERG.

A communication was prepared in conjunction with GTCS to notify all registered teachers of the proposed sharing of registration data. This included details of the rationale for the research and provided a period during which registrants could raise objections to data sharing.

## Results

By the end of June 2021, the case-control study included 132 420 cases, and 1 306 566 controls matched on age, sex, and general practice. Of 66 710 individuals in the teacher dataset 25 687 were selected (as cases or controls) into the case-control study. Of 87 273 patient facing healthcare workers, 38 993 were selected into the case-control study. Of 8501 non-patient facing healthcare workers, 2731 were selected into the case-control study. Table 1 shows the characteristics of the teachers and healthcare workers compared with the general population using reweighted data from controls. Compared with the general population, teachers and healthcare workers were similar for age and race/ethnicity but were more likely to be women and to have fewer comorbidities. Both teachers and healthcare workers were less likely to live in the most deprived fifth of areas than the general population, with a larger difference for teachers. Teachers were predominantly women; even in secondary schools, where the proportion of men was higher than that in other schools, two thirds of teachers were women. By the end of follow-up, the proportion who had received a first dose of covid-19 vaccine was similar between teachers and healthcare workers and higher than in adults of working age in the general population. Many more healthcare workers had received second doses than had the other groups. Supplementary figure S1 shows the proportion of each group vaccinated over time by age and sex.

**Table 1:** Characteristics of teachers, healthcare workers, and others selected as controls, as well as estimated characteristics of these groups in the Scottish population. Values are percentages (numbers) unless stated otherwise

Characteristics	General population	Healthcare worker status			Teaching sector				
		Patient facing	Non-patient facing		Any	Nursery and primary school	Primary school	Secondary school	Other
No of participants	1 204 818	33 461	2234		23 857	5764	5636	9126	3331
Mean (SD) age (years)	43 (13)	44 (11)	47 (10)		43 (11)	42 (11)	41 (11)	43 (11)	46 (11)
Women	49	82	66		80	91	90	69	79
Scottish index of multiple deprivation fifth:									
1 (most deprived)	20 (317 638)	13 (5593)	12 (352)		5.3 (1763)	3.7 (309)	6.6 (491)	5.5 (714)	5.2 (249)
2	20 (259 658)	17 (6414)	17 (444)		11 (3157)	10 (696)	12 (801)	11 (1256)	10 (404)
3	20 (210 136)	20 (6127)	17 (373)		20 (4451)	21 (1128)	19 (1032)	20 (1697)	19 (594)
4	20 (210 403)	25 (7487)	26 (527)		29 (6619)	29 (1656)	28 (1471)	29 (2589)	28 (903)
5 (least deprived)	20 (204 197)	26 (7742)	28 (530)		35 (7794)	36 (1957)	35 (1824)	34 (2840)	38 (1173)
Unknown	0.2 (2786)	0.2 (98)	0.3 (8)		0.2 (73)	0.2 (18)	0.3 (17)	0.3 (30)	0.2 (8)
Race/ethnicity:									
Asian	1.0 (13 653)	1.0 (337)	0.8 (19)		0.3 (81)	0.2 (10)	0.4 (21)	0.3 (35)	0.4 (15)
Black	0.4 (4871)	0.5 (166)	0.3 (6)		0.1 (24)	0.1 (6)	0.1 (-)*	0.1 (9)	0.2 (-)*
Chinese	0.2 (2908)	0.2 (58)	0.1 (-)*		0.0 (9)	0.0 (-)*	0.0 (-)*	0.1 (-)*	0.0 (-)*
Other	0.8 (10 739)	1.0 (327)	0.5 (13)		0.4 (86)	0.3 (17)	0.2 (11)	0.5 (40)	0.6 (18)
Unknown	46 (541 457)	33 (10 530)	40 (841)		39 (8992)	38 (2123)	37 (2027)	42 (3691)	36 (1151)
White	52 (631 190)	64 (22 043)	59 (1354)		60 (14 665)	62 (3608)	63 (3570)	57 (5346)	63 (2141)
Shielding	2.5 (29031)	2.3 (756)	2.2 (47)		1.6 (381)	1.5 (85)	1.8 (100)	1.6 (141)	1.7 (55)
No of comorbidities:									
None	82 (987 156)	80 (26 798)	81 (1806)		84 (20 077)	85 (4866)	84 (4743)	85 (7738)	82 (2730)
1	13 (154 399)	14 (4881)	14 (310)		12 (2916)	12 (714)	12 (690)	12 (1058)	14 (454)
≥2	5.4 (63 263)	5.3 (1782)	5.3 (118)		3.8 (864)	3.3 (184)	3.9 (203)	3.7 (330)	4.6 (147)
Household member of patient facing healthcare worker	2.4 (28 988)	-	-		2.4 (597)	2.2 (131)	2.2 (128)	2.8 (262)	2.1 (76)
Vaccination status:									
Unvaccinated	21 (269 708)	5.6 (2018)	4.7 (115)		5.4 (1421)	6.2 (387)	6.5 (401)	4.5 (445)	5.0 (188)
First dose	20 (266 197)	4.5 (1615)	11 (271)		23 (6158)	24 (1546)	26 (1625)	24 (2395)	16 (592)
Second dose	59 (668 913)	90 (29 828)	84 (1848)		71 (16 278)	70 (3831)	67 (3610)	72 (6286)	79 (2551)

* Counts <5 have been redacted.

Numbers are for observed controls, but percentages, means, and standard deviations are reweighted to account for the stratified sampling of controls by age, sex, health board area, and Scottish index of multiple deprivation.

### Risks of covid-19 by occupation

**Fig 1:**
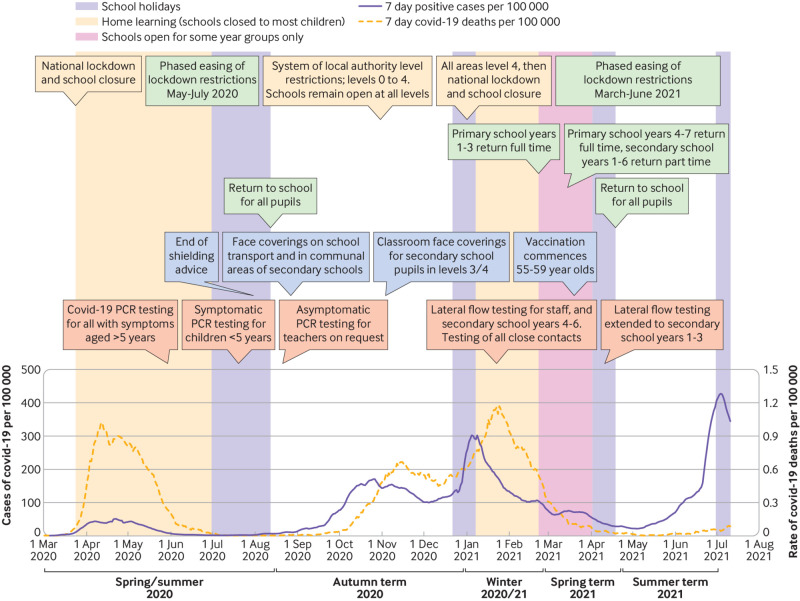
Course of covid-19 pandemic in Scotland and relation to policies in schools. PCR=polymerase chain reaction

**Fig 2:**
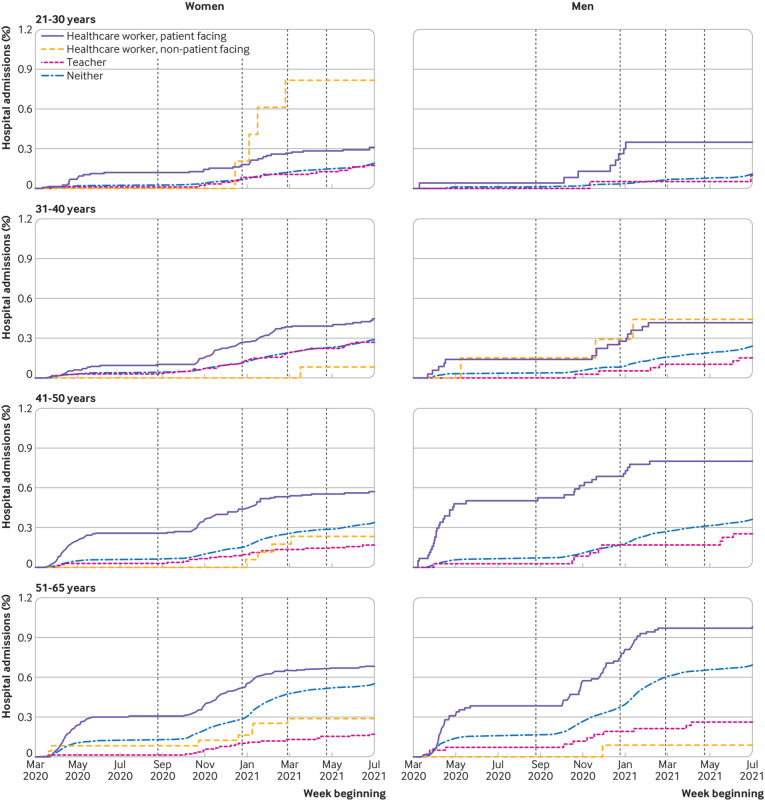
Cumulative incidence of hospital admission with covid-19. Vertical lines indicate transitions between five periods: spring/summer 2020 (closed), autumn term 2020 (open), winter 2020/21 (closed), spring term 2021 (phased), summer term 2021 (open)

Over the study period (fig 1), the cumulative incidence (risk) of hospital admission with covid-19 remained <1% for teachers, healthcare workers, and adults of working age in the general population (fig 2). The increase in risk over time in teachers was generally similar to that in non-patient facing healthcare workers and adults of working age in the general population and lower than in patient facing healthcare workers.

### Primary endpoint: hospital admission

**Fig 3:**
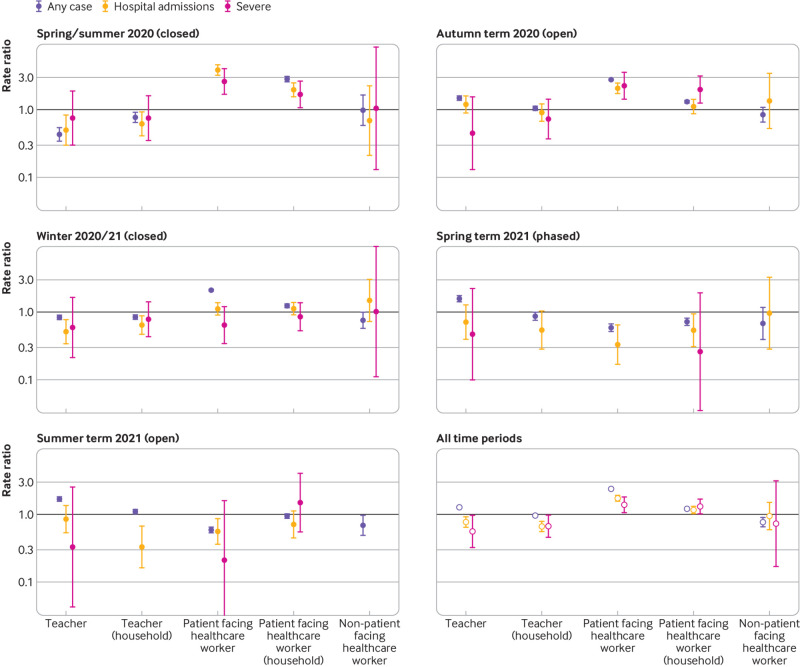
Rate ratios for any case of covid-19, hospital admission with covid-19, and severe covid-19. Figure represents graphically results shown in table 2. Circles are rate ratios and whiskers are 95% confidence intervals for adjusted models. The rate ratio for any case among healthcare workers in the period before schools reopened in 2020 is excluded as this was high, making the results for other groups more difficult to distinguish

The following rate ratios are from the fully adjusted models, but similar results were obtained for unadjusted models, which were conditional only on age, sex, and general practice (table 2, fig 3). Taking the entire study period into consideration, the risk of hospital admission with covid-19 was found to be lower in teachers (0.77, 0.64 to 0.92) and their household members (0.66, 0.56 to 0.80) compared with adults of working age in the general population. In contrast, the risk was found to be higher in patient facing healthcare workers (1.73, 1.56 to 1.91) and their household members (1.17, 1.03 to 1.33), whereas the risk in non-patient facing healthcare workers was observed to be similar to that adults of working age in the general population (0.95, 0.60 to 1.51).

**Table 2:** Rate ratios for any case of covid-19, hospital admission with covid-19, and severe covid-19 for teachers and healthcare workers and their household members

Outcome by period and model*	General population	Teachers	Household members of teachers		Patient facing healthcare workers	Household members of patient facing healthcare workers	Non-patient facing healthcare workers
**All periods: any case**							
Cases/controls:	80 124/676 522	3794/21 792	3372/21 629		10 089/29 043	5175/26 125	184/2153
Unadjusted	1	1.26 (1.22 to 1.30)	1.10 (1.07 to 1.14)		2.37 (2.32 to 2.43)	1.39 (1.35 to 1.43)	0.64 (0.55 to 0.74)
Adjusted	1	1.27 (1.22 to 1.31)	0.97 (0.93 to 1.00)		2.38 (2.33 to 2.44)	1.21 (1.17 to 1.24)	0.77 (0.66 to 0.90)
**All periods: hospital admission**							
Cases/controls:	5517/751 129	128/25 458	131/24 870		501/38 631	301/30 999	20/2317
Unadjusted	1	0.68 (0.57 to 0.82)	0.67 (0.56 to 0.80)		1.65 (1.50 to 1.83)	1.28 (1.14 to 1.45)	0.81 (0.51 to 1.28)
Adjusted	1	0.77 (0.64 to 0.92)	0.66 (0.56 to 0.80)		1.73 (1.56 to 1.91)	1.17 (1.03 to 1.33)	0.95 (0.60 to 1.51)
**All periods: severe covid-19**							
Cases/controls:	1141/755 505	15/25 571	28/24 973		66/39 066	77/31 223	2/2335
Unadjusted	1	0.47 (0.28 to 0.80)	0.67 (0.47 to 0.97)		1.22 (0.94 to 1.58)	1.44 (1.14 to 1.83)	0.49 (0.12 to 2.02)
Adjusted	1	0.56 (0.33 to 0.97)	0.67 (0.45 to 0.98)		1.39 (1.07 to 1.82)	1.31 (1.02 to 1.69)	0.73 (0.17 to 3.14)
**Spring/summer 2020 (closed): any case**							
Cases/controls:	4261/64 150	72/2349	108/1793		2640/3509	532/2272	15/231
Unadjusted	1	0.41 (0.32 to 0.52)	0.77 (0.65 to 0.92)		10.45 (9.83 to 11.11)	2.95 (2.67 to 3.25)	0.88 (0.52 to 1.48)
Adjusted	1	0.43 (0.34 to 0.54)	0.77 (0.65 to 0.92)		10.74 (10.09 to 11.43)	2.82 (2.55 to 3.12)	0.98 (0.58 to 1.66)
**Spring/summer 2020 (closed): hospital admission**							
Cases/controls:	1087/67 324	16/2405	23/1878		204/5945	94/2710	3/243
Unadjusted	1	0.44 (0.27 to 0.74)	0.58 (0.39 to 0.87)		3.49 (2.94 to 4.13)	1.96 (1.57 to 2.46)	0.62 (0.19 to 2.01)
Adjusted	1	0.50 (0.30 to 0.84)	0.62 (0.41 to 0.93)		3.86 (3.23 to 4.61)	1.97 (1.55 to 2.50)	0.69 (0.21 to 2.27)
**Spring/summer 2020 (closed): severe covid-19**							
Cases/controls:	294/68 117	5/2416	7/1894		30/6119	25/2779	1/245
Unadjusted	1	0.56 (0.23 to 1.40)	0.70 (0.34 to 1.45)		2.30 (1.52 to 3.47)	1.77 (1.16 to 2.72)	0.74 (0.10 to 5.66)
Adjusted	1	0.75 (0.30 to 1.91)	0.75 (0.35 to 1.60)		2.61 (1.69 to 4.02)	1.68 (1.07 to 2.64)	1.05 (0.13 to 8.22)
**Autumn term 2020 (open): any case**							
Cases/controls:	24 614/248 683	1424/8947	1127/8307		3807/12 498	1624/9840	64/839
Unadjusted	1	1.48 (1.40 to 1.57)	1.20 (1.13 to 1.27)		2.76 (2.66 to 2.87)	1.51 (1.44 to 1.60)	0.70 (0.55 to 0.91)
Adjusted	1	1.48 (1.40 to 1.57)	1.05 (0.98 to 1.11)		2.76 (2.66 to 2.87)	1.31 (1.25 to 1.39)	0.84 (0.65 to 1.09)
**Autumn term 2020 (open): hospital admission**							
Cases/controls:	1478/271 819	54/10 317	48/9386		160/16 145	75/11 389	5/898
Unadjusted	1	1.09 (0.82 to 1.46)	0.90 (0.67 to 1.21)		1.97 (1.65 to 2.35)	1.21 (0.95 to 1.54)	1.02 (0.41 to 2.57)
Adjusted	1	1.20 (0.89 to 1.61)	0.91 (0.67 to 1.23)		2.08 (1.73 to 2.50)	1.11 (0.87 to 1.43)	1.34 (0.52 to 3.42)
**Autumn term 2020 (open): severe covid-19**							
Cases/controls:	291/273 006	3/10 368	8/9426		24/16 281	24/11 440	0/903
Unadjusted	1	0.49 (0.15 to 1.58)	0.70 (0.36 to 1.34)		1.88 (1.21 to 2.91)	1.97 (1.28 to 3.03)	-
Adjusted	1	0.45 (0.13 to 1.55)	0.73 (0.37 to 1.44)		2.26 (1.43 to 3.59)	1.99 (1.25 to 3.17)	-
**Winter 2020/21 (closed): any case**							
Cases/controls:	26 396/252 286	813/9000	914/8642		2933/12 636	1701/10 654	57/855
Unadjusted	1	0.81 (0.76 to 0.88)	0.95 (0.89 to 1.01)		2.07 (1.99 to 2.16)	1.42 (1.35 to 1.50)	0.61 (0.47 to 0.80)
Adjusted	1	0.83 (0.77 to 0.90)	0.84 (0.78 to 0.90)		2.09 (2.01 to 2.18)	1.23 (1.17 to 1.30)	0.75 (0.57 to 0.98)
**Winter 2020/21 (closed): hospital admission**							
Cases/controls:	1726/276 956	24/9789	42/9514		106/15 463	98/12 257	9/903
Unadjusted	1	0.42 (0.28 to 0.63)	0.68 (0.50 to 0.94)		1.11 (0.90 to 1.37)	1.31 (1.06 to 1.61)	1.22 (0.61 to 2.46)
Adjusted	1	0.51 (0.34 to 0.78)	0.64 (0.47 to 0.89)		1.11 (0.90 to 1.38)	1.12 (0.90 to 1.39)	1.48 (0.72 to 3.04)
**Winter 2020/21 (closed): severe covid-19**							
Cases/controls:	398/278 284	4/9809	13/9543		11/15 558	22/12 333	1/911
Unadjusted	1	0.41 (0.15 to 1.12)	0.87 (0.49 to 1.55)		0.54 (0.29 to 1.00)	1.03 (0.66 to 1.62)	0.85 (0.11 to 6.72)
Adjusted	1	0.59 (0.21 to 1.65)	0.78 (0.43 to 1.43)		0.64 (0.34 to 1.20)	0.85 (0.53 to 1.37)	1.01 (0.11 to 8.91)
**Spring term 2021 (phased): any case**							
Cases/controls:	6882/64 102	386/2418	251/2346		224/3420	268/2973	14/231
Unadjusted	1	1.48 (1.32 to 1.65)	1.01 (0.89 to 1.15)		0.60 (0.52 to 0.69)	0.84 (0.74 to 0.95)	0.55 (0.32 to 0.94)
Adjusted	1	1.57 (1.40 to 1.75)	0.87 (0.76 to 0.99)		0.59 (0.52 to 0.68)	0.72 (0.64 to 0.82)	0.68 (0.39 to 1.18)
**Spring term 2021 (phased): hospital admission**							
Cases/controls:	550/70 434	13/2791	10/2587		9/3635	14/3227	3/242
Unadjusted	1	0.67 (0.38 to 1.18)	0.52 (0.28 to 1.00)		0.32 (0.16 to 0.63)	0.56 (0.32 to 0.96)	0.90 (0.27 to 2.97)
Adjusted	1	0.71 (0.39 to 1.27)	0.54 (0.28 to 1.03)		0.33 (0.17 to 0.65)	0.54 (0.31 to 0.95)	0.96 (0.28 to 3.24)
**Spring term 2021 (phased): severe covid-19**							
Cases/controls:	81/70 903	2/2802	0/2597		0/3644	1/3240	0/245
Unadjusted	1	0.57 (0.13 to 2.41)	-		-	0.30 (0.04 to 2.21)	-
Adjusted	1	0.47 (0.10 to 2.27)	-		-	0.26 (0.03 to 1.93)	-
**Summer term 2021 (open): any case**							
Cases/controls:	18 185/16 8350	1103/5530	976/6499		509/7496	1059/8303	34/531
Unadjusted	1	1.77 (1.65 to 1.89)	1.30 (1.22 to 1.39)		0.61 (0.55 to 0.67)	1.09 (1.02 to 1.16)	0.56 (0.40 to 0.79)
Adjusted	1	1.69 (1.58 to 1.81)	1.10 (1.03 to 1.18)		0.59 (0.54 to 0.65)	0.94 (0.88 to 1.00)	0.69 (0.48 to 0.97)
**Summer term 2021 (open): hospital admission**							
Cases/controls:	676/185 859	21/6612	8/7467		22/7983	20/9342	0/565
Unadjusted	1	0.77 (0.49 to 1.21)	0.33 (0.16 to 0.68)		0.55 (0.35 to 0.85)	0.80 (0.51 to 1.25)	-
Adjusted	1	0.85 (0.54 to 1.36)	0.33 (0.16 to 0.67)		0.56 (0.36 to 0.87)	0.71 (0.45 to 1.13)	-
**Summer term 2021 (open): severe covid-19**							
Cases/controls:	79/186 456	1/6632	0/7475		1/8004	5/9357	0/565
Unadjusted	1	0.26 (0.03 to 1.91)	-		0.23 (0.03 to 1.70)	1.54 (0.59 to 4.04)	-
Adjusted	1	0.33 (0.04 to 2.55)	-		0.21 (0.03 to 1.60)	1.49 (0.55 to 4.07)	-

As these were conditional logistic regression models, in addition to the variables shown in the table, unadjusted models were nonetheless conditional on age, sex, and general practice.

* Models were adjusted for race/ethnicity, Scottish index of multiple deprivation, number of comorbidities, and number of adults in the household. Counts of controls in the table for teachers, and hence in the general population, do not sum to equal the counts shown in table 1 for two reasons. Firstly, for computational reasons strata where none of the cases or controls were teachers, healthcare workers, or household members were excluded from the regression modelling (see methods). Secondly, because individuals changed status between teachers and non-teachers during these periods; this table indicates each person’s status during the time when they experienced the event (or were selected as a control), whereas table 1 counts individuals as teachers if they were assigned that category at any time during the follow-up period. Individuals who are household members of both teachers and healthcare workers are counted here only once as household members of teachers, although the regression modelling allowed for individuals to be classified as household members of neither, either, or both.

Similarly, in the initial period of school closure (spring/summer 2020), the risk of hospital admission with covid-19 was found to be lower in teachers (0.50, 0.30 to 0.84) and their household members (0.62, 0.41 to 0.93) than in adults of working age in the general population (table 2). During this same period, the risk was found to be higher in patient facing healthcare workers (3.86, 3.23 to 4.61) and their household members (1.97, 1.55 to 2.50) but similar in non-patient facing healthcare workers (0.69, 0.21 to 2.27). During the later period of school closure (winter 2020/21), teachers and their household members again showed a lower risk of hospital admission than adults of working age in the general population (0.51, 0.34 to 0.78 and 0.64, 0.47 to 0.89, respectively), whereas the risk in the other groups was observed to be similar to that in adults of working age in the general population.

In the first period of full school opening (autumn term 2020), the risk of hospital admission in teachers was observed to be higher than in the initial period when schools were closed (1.20, 0.89 to 1.61), although with 95% confidence intervals that included 1. For the same period, the rate ratios for non-patient facing healthcare workers and household members of teachers and of healthcare workers were all close to 1—respectively, 1.34 (0.52 to 3.42), 0.91 (0.67 to 1.23), and 1.11 (0.87 to 1.43). Patient facing healthcare workers continued to have higher rates of hospital admission than adults of working age in the general population in this period (2.08, 1.73 to 2.50). When the risk in teachers was compared between this period and spring/summer 2020 (ie, a difference-in-differences approach) when schools were closed, the rate ratio was 2.37-fold higher (95% confidence interval 1.31-fold to 4.28-fold).

In the second period of full school opening (summer term 2021), the risk of hospital admission for teachers was again observed to be higher than in the initial period before schools closed (rate ratio 0.85, 95% confidence interval 0.54 to 1.36), although the point estimate remained <1 (ie, lower than the general population average). The risk for patient facing healthcare workers was found to be lower (0.56, 0.36 to 0.87), and the risk for household members of each group was close to 1. When the risk for teachers in this period was compared with the risk for teachers in the spring/summer 2020 period when schools were closed, the rate ratio was 1.69-fold higher (95% confidence interval 0.85-fold to 3.38-fold), with a confidence interval that included the null.

Whether these differences in risk between the 2020 and 2021 periods when schools were open were related to vaccination status was explored in analyses that were prespecified before extraction of the most recent data. On repeating the summer 2020/21 comparison after restricting the analysis to cases and controls who were either unvaccinated or within 14 days of their first vaccine, the risks of hospital admission among teachers and patient facing healthcare workers (compared with adults of working age in the general population) were found to be similar to those observed in the autumn 2020 term when schools were open: rate ratios 1.42 (95% confidence interval 0.78 to 2.60) and 1.89 (0.72 to 4.96), respectively. As only seven hospital admissions with covid-19 occurred after the first dose among teachers and 39 among patient facing healthcare workers, the number of events were insufficient to estimate relative risks among vaccinated people. Supplementary figure 2 shows the full results for unvaccinated individuals.

### Secondary endpoint: severe covid-19

Throughout the entire pandemic period, the risk of severe covid-19 among teachers compared with adults of working age in the general population was observed to be similar or lower (0.56, 0.33 to 0.97; table 2, fig 3). In contrast, patient facing healthcare workers showed an increased risk (1.39, 1.07 to 1.82) as did their household members (1.31, 1.02 to 1.69). The risk observed in household members of teachers and non-patient facing healthcare workers was lower than in adults of working age in the general population (0.67, 0.45 to 0.98 and 0.73, 0.17 to 3.14, respectively), although the 95% confidence intervals for both were wide and for non-patient facing healthcare works included the null. No evidence was found for an increased risk of severe covid-19 among teachers in the autumn term of 2020 when schools were open (fig 3); instead, the risk was observed to reduce, with a 0.59-fold difference (95% confidence interval 0.13-fold to 2.80-fold) compared with the earlier period, although this confidence interval was wide and includes the null. During the second period when schools were open (summer term 2021), the risk was also observed to decrease for teachers; the confidence intervals at this time, however, were wide, reflecting that this estimate is based on only one event among teachers.

### Non pre-specified endpoint: any case of covid-19

For any case of covid-19 in the period when schools were closed (spring/summer 2020), teachers showed a lower risk (0.43, 0.34 to 0.54), as did household members of teachers (0.77, 0.65 to 0.92), while the rates were markedly higher among patient facing healthcare workers (10.74, 10.09 to 11.43), and household members of healthcare workers (2.82, 2.55 to 3.12). In the first period of school opening (autumn term 2020), the risk was observed to be higher than in adults of working age in the general population among teachers (1.48, 1.40 to 1.57), healthcare workers (2.76, 2.66 to 2.87), and household members of healthcare workers (1.31, 1.25 to 1.39), but the risk in household members of teachers was similar to that of the general population (1.05, 0.98 to 1.11). During the second period when schools were open (summer term 2021), the risk for teachers was found to increase (1.69, 1.58 to 1.81). The supplementary file provides details on testing of teachers and healthcare workers. Rates in non-patient facing healthcare workers were <1 throughout all periods (0.77, 0.66 to 0.90).

### Risk of covid-19 by sector

The risks of hospital admission were broadly similar across different teaching sectors. During the autumn 2020 term, however, when most schools were open, lower rate ratios were observed among teachers in the nursery and primary school category (0.64, 0.27 to 1.51) and primary schools (0.99, 0.51 to 1.93) than among teachers in secondary schools (1.35, 0.88 to 2.07) and among others (1.81, 0.95 to 3.44). The numbers of events were, however, low and in all cases the confidence intervals were wide. Moreover, these differences were not found in the second period of schools reopening (table 3).

**Table 3:** Rate ratios for any case of covid-19, hospital admission with covid-19, and severe covid-19 for teachers by sector

Outcome by period and adjustment*	Primary, with nursery onsite	Primary with no nursery onsite	Secondary	Other
**All periods: any case**				
Cases/controls:	910/5316	1111/4991	1321/8426	452/3059
Unadjusted	1.27 (1.18 to 1.36)	1.53 (1.44 to 1.63)	1.14 (1.07 to 1.21)	1.10 (1.00 to 1.22)
Adjusted	1.27 (1.18 to 1.36)	1.54 (1.44 to 1.64)	1.15 (1.09 to 1.22)	1.10 (1.00 to 1.22)
**All periods: hospital admission**				
Cases/controls:	27/6199	21/6081	58/9689	22/3489
Unadjusted	0.68 (0.46 to 1.01)	0.51 (0.33 to 0.79)	0.74 (0.57 to 0.97)	0.74 (0.48 to 1.15)
Adjusted	0.76 (0.51 to 1.13)	0.59 (0.38 to 0.92)	0.84 (0.64 to 1.10)	0.84 (0.54 to 1.30)
**Spring/summer 2020 (closed): any case**				
Cases/controls:	15/583	17/531	20/894	20/341
Unadjusted	0.36 (0.21 to 0.60)	0.44 (0.27 to 0.71)	0.29 (0.19 to 0.46)	0.73 (0.46 to 1.15)
Adjusted	0.38 (0.23 to 0.64)	0.47 (0.29 to 0.76)	0.30 (0.19 to 0.47)	0.77 (0.49 to 1.22)
**Spring/summer 2020 (closed): hospital admission**				
Cases/controls:	2/596	3/545	7/907	4/357
Unadjusted	0.28 (0.07 to 1.16)	0.38 (0.12 to 1.19)	0.48 (0.22 to 1.02)	0.63 (0.23 to 1.75)
Adjusted	0.28 (0.07 to 1.18)	0.41 (0.13 to 1.31)	0.57 (0.26 to 1.23)	0.78 (0.28 to 2.18)
**Autumn term 2020 (open): any case**				
Cases/controls:	290/2096	407/2240	532/3370	195/1241
Unadjusted	1.32 (1.17 to 1.50)	1.66 (1.50 to 1.85)	1.46 (1.33 to 1.60)	1.47 (1.26 to 1.71)
Adjusted	1.33 (1.17 to 1.51)	1.65 (1.48 to 1.84)	1.47 (1.34 to 1.62)	1.46 (1.25 to 1.70)
**Autumn term 2020 (open): hospital admission**				
Cases/controls:	6/2380	10/2637	26/3876	12/1424
Unadjusted	0.59 (0.26 to 1.35)	0.89 (0.46 to 1.70)	1.25 (0.83 to 1.89)	1.68 (0.91 to 3.10)
Adjusted	0.64 (0.27 to 1.51)	0.99 (0.51 to 1.93)	1.35 (0.88 to 2.07)	1.81 (0.95 to 3.44)
**Winter 2020/21 (closed): any case**				
Cases/controls:	198/2223	231/2083	283/3420	101/1274
Unadjusted	0.80 (0.69 to 0.93)	0.98 (0.86 to 1.13)	0.74 (0.66 to 0.84)	0.74 (0.60 to 0.91)
Adjusted	0.81 (0.70 to 0.94)	1.00 (0.87 to 1.15)	0.77 (0.68 to 0.87)	0.76 (0.62 to 0.93)
**Winter 2020/21 (closed): hospital admission**				
Cases/controls:	8/2413	3/2311	10/3693	3/1372
Unadjusted	0.66 (0.32 to 1.35)	0.28 (0.09 to 0.88)	0.41 (0.22 to 0.78)	0.30 (0.09 to 0.95)
Adjusted	0.75 (0.36 to 1.57)	0.33 (0.10 to 1.05)	0.53 (0.28 to 1.00)	0.37 (0.12 to 1.18)
**Spring term 2021 (phased): any case**				
Cases/controls:	121/564	146/596	78/929	41/329
Unadjusted	2.00 (1.64 to 2.45)	2.27 (1.89 to 2.73)	0.78 (0.62 to 0.98)	1.15 (0.83 to 1.60)
Adjusted	2.15 (1.75 to 2.64)	2.44 (2.02 to 2.95)	0.81 (0.64 to 1.03)	1.18 (0.84 to 1.64)
**Spring term 2021 (phased): hospital admission**				
Cases/controls:	5/680	2/740	5/1002	1/369
Unadjusted	1.27 (0.49 to 3.28)	0.41 (0.10 to 1.71)	0.63 (0.25 to 1.56)	0.38 (0.05 to 2.81)
Adjusted	1.53 (0.56 to 4.18)	0.47 (0.11 to 1.99)	0.63 (0.25 to 1.59)	0.38 (0.05 to 2.89)
**Summer term 2021 (open): any case**				
Cases/controls:	286/1363	310/1265	410/2183	97/719
Unadjusted	1.87 (1.64 to 2.13)	2.15 (1.89 to 2.44)	1.66 (1.49 to 1.84)	1.23 (0.99 to 1.52)
Adjusted	1.75 (1.54 to 2.00)	2.08 (1.83 to 2.36)	1.60 (1.43 to 1.78)	1.18 (0.95 to 1.46)
**Summer term 2021 (open): hospital admission**				
Cases/controls:	6/1643	3/1572	10/2583	2/814
Unadjusted	0.92 (0.39 to 2.13)	0.49 (0.15 to 1.57)	0.92 (0.48 to 1.78)	0.53 (0.13 to 2.22)
Adjusted	0.94 (0.40 to 2.20)	0.60 (0.18 to 1.95)	1.06 (0.54 to 2.07)	0.52 (0.12 to 2.27)

As these were conditional logistic regression models, in addition to the variables shown in the table, unadjusted models were nonetheless conditional on age, sex, and general practice of registration.

* Adjusted for race/ethnicity, Scottish index of multiple deprivation, number of comorbidities, and number of adults in household.

The supplementary file provides event numbers and effect estimates from regression models of each outcome by age, sex, and more granular time periods, and further comparisons across time periods. The analysis code is available at the project’s GitHub repository (https://github.com/dmcalli2/tchr).

## Discussion

Among all adults of working age in Scotland, for the period from the first case of covid-19 to 28 July 2021 (including two periods when schools were largely closed, two periods when schools were fully open, and one period of phased reopening), we examined the risks of covid-19 among teachers and their household members. Neither was shown to be at increased risk of hospital admission or severe covid-19 at any time, whether compared with healthcare workers or with adults of working age in the general population; including during the periods when schools were fully open. These findings were robust to adjustment for age, sex, socioeconomic deprivation, geography, race/ethnicity, household composition, and number of comorbidities.

The risk of hospital admission for teachers was around half of that seen in the general population in each of two periods when schools were closed, but increased by around 2.4-fold during the autumn term of 2020 when schools were open—this increase suggests that the risk to teachers during the autumn 2020 term was similar to that in the general population. In the summer term of 2021, when schools were also open and vaccination of the Scottish population was underway, a smaller increase of around 1.7-fold was seen. No accompanying increase in the relative risk of severe covid-19 was observed during either period; instead the relative risks for severe covid-19 appeared to decrease, although the confidence intervals were wide. In additional analyses restricted to cases and controls who were not yet vaccinated, the increase in the risk of hospital admission among such teachers during the summer term of 2021 was similar to that seen in the autumn term of 2020, although the confidence intervals were wide.

### Strengths and limitations of this study

Our study has several strengths. This was a large and largely complete sample of teachers and their household members, and the outcome data were obtained in the same manner for the different occupational groups such that valid comparisons could be made, especially for outcomes such as hospital admissions and admissions to intensive care. The data cover periods of full school opening with in-person teaching when community transmission of covid-19 was considerable, including when both the alpha variant (winter 2020/21) and delta variant (summer term 2021) were dominant in Scotland. We were also able to examine associations for teachers in the context of partial vaccination.

Our study also has several limitations. Firstly, we were unable to link a small number of GTCS registrants to healthcare records. Secondly, the incidence and therefore precision of the estimates was low for severe covid-19, particularly in the more recent periods. Finally, we did not have detailed information on the specific circumstances of individual teachers for factors such as class size and control measures within individual schools, although we have provided information on these factors for all of Scotland (alongside infection rates) so that readers might judge the applicability of our findings to other settings.

### Comparison with other studies

Several studies have examined the risks of covid-19 among adults working with children. A Norwegian study, covering periods when schools were both closed and open, provided estimates of the risk of hospital admission with covid-19, adjusting for age, sex, and country of birth, compared with the general population of adults. The overall estimate was found to be higher in preschool teachers (relative risk 1.86, 95% confidence interval 0.97 to 3.57), lower in primary school teachers (0.73, 0.44 to 1.22), and null in secondary school teachers (1.01, 0.46 to 2.21), although the confidence intervals were wide. In the period when schools were open (18 July to 18 October 2020), the risks of any covid-19 (SARS-CoV-2 on PCR testing or a coded diagnosis, or both) appeared modestly increased in primary school teachers (1.14, 0.99 to 1.32), secondary school teachers (1.10, 0.82 to 1.47), and childcare workers (1.15, 1.02 to 1.29) but not in preschool teachers (0.73, 0.54 to 0.99).^3^

In Sweden, where schools remained mostly open, lower secondary school teachers (who taught in person) were compared with upper secondary school teachers (who did not). On adjusting for age, sex, income, and region, the relative risks for hospital admission and death with covid-19, testing positive for SARS-CoV-2, and diagnosis of covid-19, were around twofold higher in the teachers in lower secondary school than those in upper secondary school.^5^ However, an increased risk was not consistently found in other analyses compared with non-teaching occupations. Compared with IT technicians, teachers in Sweden were found to not be at increased risk of mortality from covid-19 (adjusting for age, sex, country of birth, living in Stockholm, educational attainment, and income), although the confidence intervals were wide (rate ratio 0.91, 95% confidence interval 0.55 to 1.51).^6^ Compared with other occupations, the age-sex adjusted risk of admission to intensive care with covid-19 was observed to be slightly increased among teachers of preschool children (rate ratio 1.10, 95% confidence interval 0.49 to 2.49) and lower among school teachers (0.43, 0.28 to 0.68).^7^ Therefore, the differences within secondary teachers in Sweden might be associated with a protective effect of working at home rather than a harmful effect of working in a school setting. These findings are consistent with our own observations that the rate ratio for hospital admission with covid-19 for teachers is close to 1 when schools are opened but close to 0.5 when schools are closed, and that the risk of severe covid-19 in teachers remained low throughout the pandemic.

In the UK, the Office for National Statistics examined deaths associated with covid-19 from 9 March 2020 to 28 December 2020 and found that teaching and educational professionals had lower age standardised mortality than all residents of England and Wales aged 20 to 64 years, both among men (age-standardised mortality rate 18.4, 95% confidence interval 14.0 to 23.6 *v* 31.4, 30.6 to 32.3, ranked 19th of 24 occupations from highest to lowest mortality) and among women (9.8, 7.5 to 12.5 *v* 16.8, 16.2 to 17.5, ranked 15th of 20 from highest to lowest mortality).^18^ Mortality data covering a longer period in Scotland (1 March 2020 to 30 June 2021) shows a lower age standardised mortality for teaching and education professionals of 7.1 per 100 000 (95% confidence interval 2.7 to 11.6 per 100 000), with this grouped ranked 19 out of 20 occupational groups with a rate calculated.^19^

In our study, we also reported on the risk of any covid-19 in teachers compared with adults of working age in the general population, and we found a 1.4-fold increase in the period after schools opened. In all groups this measure is largely driven by positive test results in people who were not admitted to hospital or severely ill. We explicitly did not prespecify this as an outcome because it is subject to ascertainment bias as a result of unmeasurable variation in testing policies and practices. These factors influence not only the likelihood of testing but also the timing and circumstances of testing, such as the presence of symptoms or regular screening tests; we include this outcome here for completeness only. In a random sample of the UK population who are not susceptible to such biases, the ONS survey found that between September 2020 and January 2021, teaching and education professionals showed strong evidence of a higher probability of testing positive than six of 24 other occupational groups, and no evidence of a difference from 15 other groups.^12^ The ONS School Infection Survey might provide additional information, although with fewer than 5000 adult participants it will be unable to report on the risks of hospital admission with covid-19, which was the focus of our analysis, and has not yet produced analyses comparing the risk in teachers after adjusting for age and sex.^20^ In a non-random design, Public Health Scotland offered SARS-CoV-2 antibody testing to all people working in early learning and school settings in Scotland in October and November 2020. Overall, 12 171 teachers opted to participate, and the prevalence of positive antibodies among teaching and teaching support staff was 7.1% (95% confidence interval 6.6% to 7.6%),^19^ broadly similar to rates found for those aged 16 and older in a nationally representative household survey in October 2020 (7.1%, 4.6% to 10.4%).^13^ Consequently, we would favour these findings over our own in examining the risks of any case of covid-19 among teachers.

The international and UK based findings for hospital admission and death are, however, broadly consistent with our own observations, showing that teachers are not at increased risk of hospital admission with covid-19 and are at lower risk of severe covid-19 (admission to intensive care unit or death) compared with other adults of working age in the general population. To these previous reports, which generally adjusted for age, sex, and socioeconomic status, we add evidence that differences in household composition or the prevalence of common underlying conditions does not account for the lower risk of severe covid-19 outcomes among teachers. We also add the important observation that adults of working age who are household members of teachers are not at increased risk compared with adults of working age in the general population. Also, as Scotland had among the highest rates of covid-19 in Europe during the summer term of 2021, we also add results on the risks to teachers and their household members when the delta variant is common.

Several explanations are plausible for the lower risk of hospital admission with covid-19 we observed among teachers (compared with adults of working age in the general population), having adjusted for age, sex, socioeconomic deprivation, household composition, geography, race/ethnicity, and comorbidities, during the periods when schools were closed. Firstly, teachers might be more careful about potential exposure to SARS-CoV-2 (eg, more rigorous observation of social distancing rules, increased attention to ventilation) than other adults, thereby reducing their risk of infection. Secondly, teachers might differ from other groups for incompletely ascertained comorbidities or unmeasured lifestyle risk factors (eg smoking, diet, and physical exercise) known to affect the risk of covid-19. Thirdly, teachers might have more efficient immune responses after exposure to SARS-CoV-2 owing to the presence of cross reactive T cells^21^ from increased previous occupational exposures to viruses. Finally, during periods when schools are closed, teachers might have low numbers of contacts compared with other adults of working age, such that, even where they have similar underlying health, teachers are at lower risk of covid-19. Some support for this last suggestion comes from the finding that working exclusively from home is associated with a lower risk of covid-19,^22^ and in our view this is the most plausible explanation for the lower risk among teachers during the period when schools were closed.

This latter explanation is also consistent with the observation that when teachers returned to in-person teaching, the risks of hospital admission with covid-19 were observed to increase. It is perhaps surprising that unlike among healthcare workers no commensurate increases were seen for severe covid-19 among teachers. Nor was the risk of covid-19 noticeably increased among household members of teachers. Potential explanations for these discrepant findings include a lack of statistical power to detect differences in the risk of severe covid-19 and differences in onward transmission of infection to household members, increased diagnostic activity resulting in increased hospital admission among teachers for a given severity of covid-19 (ie, an artefactual increase in hospital admission), and unaccounted differences in comorbidity or behaviour, or differences in immunity more strongly impacting the observed associations for severe covid-19 than for hospital admission with covid-19.

### Policy implications

Notwithstanding mechanisms, our observations are likely to be of interest to teachers, their household members, policy makers, and the wider population. Whether teachers are generally healthier, more careful about covid-19 related behaviours, or other explanations exist, the observation remains that after adjusting for age, sex, location, socioeconomic deprivation, race/ethnicity, comorbidities, and household composition, the risk of hospital admission with covid-19 in teachers was similar to that in adults of working age in the general population, even when schools were open. In contrast, the risk of hospital admission with covid-19 was found to be higher among patient facing healthcare workers and their household members. Non-patient facing healthcare workers, as with teachers, were not observed to be at increased risk compared with adults of working age in the general population.

We also found that, compared with adults of working age in the general population, teachers were not at increased risk of hospital admission with covid-19 when schools were open, vaccine uptake was high among teachers, the delta variant was dominant, and a large surge in cases occurred. We believe that this has important policy implications for both the vaccination of teachers and the delivery of education as schools plan for the autumn 2021 term.

### Conclusion

In our study, most of the teachers were young, were women, and had few comorbidities and so were at low absolute risk of severe covid-19 and hospital admission with covid-19. Furthermore, compared with healthcare workers and with other adults of working age who are otherwise similar, teachers showed no increased risk of hospital admission with covid-19 or severe covid-19. These findings should reassure most adults engaged in in-person teaching.

**What is already known on this topic**

The rates of covid-19 and mortality are higher in some occupational groups than others
Existing studies do not indicate that teachers have been at increased risk of hospital admission with covid-19, although some variation was found by type of teacher, and predate the widespread circulation of alpha and delta variants
Contemporary data on the risk of covid-19 among teachers, which is critical to inform decision making on schools and education, is therefore needed

**What this study adds**

From March 2020 to June 2021 in Scotland, no evidence was found that the risk of admission to hospital with covid-19 was higher among teachers than among other adults of working age in the general population, after adjusting for age, sex, socioeconomic deprivation, location, race/ethnicity, household composition, and comorbidities
When schools were largely closed, teachers showed a lower risk of being admitted to hospital with covid-19 than other adults of working age, and when schools were fully open, the risk in both groups was similar
Prompt uptake of vaccination in teachers might have contributed to their protection during a period when the delta variant was common

## Data Availability

Access to data for third parties would require agreement of the organisations who provided data to Public Health Scotland (NHS Education Scotland and the General Teaching Council Scotland).

## Web extra

Extra material supplied by authors

## Notes

The lead author (DM) affirms that the manuscript is an honest, accurate, and transparent account of the study being reported; that no important aspects of the study have been omitted; and that any discrepancies from the study as planned (and, if relevant, registered) have been explained.

Dissemination to participants and related patient and public communities: This research has been made available to policy makers and key education stakeholders through the presentation of results to the Scottish Government CERG and the Scottish Government Advisory Sub-Group on Education and Children’s Issues. Study authors have engaged in meetings with professional associations representing teachers to discuss information on covid-19 and education, including communication of and reflections on these results. A plain language summary and visualisation of this research will be prepared and shared via social media and through educational and public health networks.

Provenance and peer review: Not commissioned; externally peer reviewed.
