# Supplementary material for: Risk of hospital admission with covid-19 among teachers compared with healthcare workers and other adults of working age in Scotland, March 2020 to July 2021: population based case-control study
Source: BMJ. 2021 Sep 2;374:n2060. doi: 10.1136/bmj.n2060 (PMC8408959; doi:10.1136/bmj.n2060)
Supplement: Supplementary file 1 — Supplementary information: additional material [file fenl064704.ww1.pdf]

Supplementary appendix to Risk of hospitalisation with COVID-19 among teachers compared to healthcare workers and other working-age adults. A nationwide case-control study

Fenton L, Gribben C, Caldwell D, Colville S, Bishop J, Reid M, White J, Goldberg D, Hutchinson S, Robertson C, Colhoun H, Wood R, McKeigue P, McAllister DA

30/07/2021

Exposure status and the GTCS register

All teachers working in the state sector are included on the General Teaching Council for Scotland (GTCS) register, and from 1st October 2017 all newly appointed teachers working at independent schools have also been required to register. However, the deadline for registration for those already in post prior to the 1st of October 2017 is not until 1st of June 2021, so some teachers working in independent schools will not have been included (https://www.gtcs.org.uk/registration/independent-schools-registration.aspx). Teachers were defined by the GTCS as actively teaching if they had completed their "professional update" (the ongoing professional learning requirement that is a requirement for employment as a teacher) and had a last known employer suggestive of a teaching post. A small set of teachers (< 400) were removed from the data before it was provided to PHS at their own request following a consultation held by the GTCS.

Since case/control status is defined at a specific date (generally the date at which a case tested positive for both cases and their matched control), teachers believed **not** to be actively teaching at the this time-point were not defined as teachers for this analysis; within the case-control study, 44 individuals were not actively teaching in February but were in November and 309 teachers were actively teaching in February but not in November. Teachers were further sub-divided based on their self-reported setting. The original settings were collapsed into the following sectors; nursery and primary, primary, secondary and other. Few primary teachers in Scotland are based in nurseries. As such, the majority of teachers allocated to the "nursery and primary" category are teachers of primary-school-aged children (based in schools with on-site nurseries) rather than teachers of pre-school children working within nurseries. The "other" group comprised further education colleges, local government, "miscellaneous", "nursery/primary/special", "primary/special" and "special".

Overview of schools in Scotland during the pandemic

Schools in Scotland

There are nearly 2,500 schools in Scotland, providing education for over 700,000 pupils aged 4-18 years. Children spend seven years in primary school, starting at age 4.5-5.5 years (with classes labelled P1-P7), and up to six years in secondary school (S1-S6). In 2020, the average primary school class size was 23.1 pupils. The overall teacher to pupil ratio in publicly funded schools is 1:15.4 in primary schools, 1:12.5 in secondary, and 1:3.8 in special schools. Of 357 secondary schools, three-quarters (75.1%) have 600 or more pupils, and more than two-thirds (36.1%) have 1,000 or more pupils. The majority of children (73%) attend schools in large urban areas or other urban areas, with 14% attending schools in small towns, and 13% those in rural areas. [Scottish Government. Pupil census 2020 supplementary statistics. https://www.gov.scot/publications/pupil-census-supplementary-statistics/ Accessed 30/04/2021]

Periods of home learning and attendance levels

Figure 1 provides a timeline showing periods of lockdown, school closures, and date of implementation of relevant control measures. In the period following the implementation of a national lockdown at the end of March 2020, nearly all education was moved to home learning. Care in education 'hubs' was maintained for the children of key workers who could not access other childcare, and for vulnerable children. Fewer than 2% of all pupils attended such provision per day, along with an average of just under 8,000 teaching staff present on school sites each day. [Scottish Government. Attendance and absence data for pupils and staff in schools and childcare settings. https://public.tableau.com/profile/sg.eas.learninganalysis#!/vizhome/Covid19ELCandHubs/Introduction?publish=yes Accessed 30/04/2021]. From August to December 2020 schools were fully re-opened for in-person, full-time learning in the school environment. Daily attendance over this period was around 90%, which is comparable to that seen in the 2018/19 academic year. [Scottish Government. School attendance and absence statistics. https://www.gov.scot/publications/school-attendance-and-absence-statistics/ Accessed 30/04/2021].

Following a surge of cases at the end of 2020, associated with spread of the B.1.1.7 variant, there was a further national lockdown and a return to learning at home for the majority of children. School sites did remain open for vulnerable children, and the children of key workers, and in this period (Jan-Feb 2021) around 7% of children attended each day. [Scottish Government. Attendance and absence data for pupils and staff in schools and childcare settings. https://public.tableau.com/profile/sg.eas.learninganalysis#!/vizhome/Covid19ELCandHubs/Introduction?publish=yes Accessed 30/04/2021].

A phased return to in-person education commenced on 22/02/2021 with all P1-3 pupils returning full-time (42% of the primary pupil population) and a small proportion of S4-6 pupils returning part-time (to a maximum of 8% of secondary pupils on site at any time). [Scottish Government. News 'Back to school'. https://www.gov.scot/news/back-to-school-1 Accessed 30/04/2021]. From 15/03/2021 all primary pupils returned full-time and all secondary pupils part-time with two-metre physical distancing for. [Scottish Government. News 'Phase 2 of schools return'. https://www.gov.scot/news/phase-2-of-schools-return Accessed 30/04/2021]. From mid-April, following a holiday period, all pupils have returned full-time, with secondary pupils no longer required to be physically distanced. Scotland remained in lockdown until 26/04/2021, in terms of travel restrictions, and closure of non-essential retail and hospitality.

Recommended measures within schools

A table summarising the recommended measures for reducing risk in schools, and relevant policies regarding testing and contact tracing, is provided below. Key points include

- Physical distancing –two-metre distancing required between staff and staff and pupils; encouraged 'where possible' for secondary pupils; not required between primary pupils.
- Face coverings – required for staff in communal areas, and when face-to-face interaction at a distance of less than two metres required for greater than 15 minutes. Not required within school at any point for primary school children. Required for secondary school pupils whilst in communal areas of the school, such as corridors. From 02/11/2020, following the introduction of a tiered system of local control measures, senior (S4-6) pupils attending schools in higher incidence (level 3 and 4) areas were required to wear face coverings in the classroom. The majority of local authority areas (24 out of 32 areas, covering 86% of the S4-6 pupil population) were in these levels for at least some of that period, however overall only for 12% of the total S4-6 'pupil weeks' in the period Aug-Dec 2020 were these pupils required to wear face coverings in class. Required for all secondary staff and pupils and in all areas from March 2021. [Scottish Government. Coronavirus (COVID-19): guidance on reducing the risk

in schools. https://www.gov.scot/publications/coronavirus-covid-19-guidance-on-reducing-the-risks-in-schools/pages/scientific-and-public-health-advice. Accessed 30/04/2021 The Scottish Parliament. Coronavirus (COVID-19): Protection levels. https://spice-spotlight.scot/2021/03/24/coronavirus-covid-19-protection-levels/ Accessed 30/04/2021]

- Testing – in the early pandemic PCR testing was restricted and of the groups represented here, would have been largely limited to health care workers. From late May 2020 testing was available to all those aged >5 years with symptoms of COVID-19. From Aug 2020 teachers could access asymptomatic PCR tests if they wished to. An at-home lateral flow device testing programme was implemented from 15/02/2021, offering twice weekly testing to all people who work in schools, and S4-6 pupils; this was extended to S1-3 pupils from April 2021. Throughout the pandemic there was no specific additional testing measures or programme in place for school clusters, although asymptomatic testing for school and local community populations was occasionally used in this scenario from Feb 2021.

•	**Primary pupils**	**Secondary pupils S1-3**	**Secondary pupils S4-6**	**Staff**
Physical distancing	No	Encouraged (required for 3 weeks from 15/3/2021)	Encouraged (required for 3 weeks from 15/3/2021)	Recommended
Face coverings	No	In communal areas from Aug 2020.	In communal areas from Aug 2020.	In communal areas from Aug 2020.
		In classroom from April 2021.	In classrooms for pupils in level3/4 areas from Nov 2020, and for all pupils from Mar 2021.	In classrooms when teaching S4-6 pupils in level3/4 areas from Nov 2020.
				At all times in secondary schools from Jan 2021.
Testing	Symptomatic testing from mid-May 2020 (late July 2020 for those under 5 years). Testing of close contacts from mid-Feb 2021.	Symptomatic testing from mid-May 2020. Testing of close contacts from mid-Feb 2021.	Symptomatic testing from mid-May 2020.	Symptomatic testing from mid-May 2020. Asymptomatic PCR testing available on request from Aug 2020.
		Twice weekly at-home LFD testing from April 2021.	Testing of close contacts from mid-Feb 2021.	Testing of close contacts from mid-Feb 2021.
			Twice weekly at-home LFD testing from mid-Feb 2021.	Twice weekly at-home LFD testing from mid-Feb 2021.

	**All groups**
Individuals at highest clinical risk – ‘shielding’	Individual risk assessments to inform decisions. General principles: able to return to school from Aug 2020; following introduction of local protection levels in Nov 2020 those in level 4 areas to work from home/home learning.
Hand hygiene	Measures to encourage and ensure hand hygiene set out in guidance
Environmental cleaning	Enhanced environmental cleaning regime described in guidance
Ventilation	Guidance recommends measures to ensure adequate ventilation
Personal Protective Equipment (PPE)	Not required for routine educational activities. Guidance provides recommendations for particular circumstances, such as cases of suspected COVID-19 and provision of intimate care.

Contact tracing	Same definitions of direct (<1m) and proximity (<2m for >15mins) non-household contacts applied in educational settings, as elsewhere. Schools support identification of contacts. Contacts required to self-isolate for 10 days from exposure. Testing of all contacts implemented mid-Feb 2021 (only advised for symptomatic contacts prior to this).
Incident management	Incidents of potentially linked cases managed by local Health Board Health Protection Teams. No general programme of increased testing in the context of incidents, but used to investigate some clusters linked to educational settings from Feb 2021. Decisions about closure of specific schools for infection control or due to staffing difficulties are made at local level.
'Grouping' to minimise contacts among pupils	'Grouping' is the recommended approach of keeping pupils in consistent groups throughout the school day, and minimising contacts between groups, for example through one-way corridors and staggered break times. These measures are applied in different ways according to educational requirements and physical constraints.

https://www.gov.scot/publications/coronavirus-covid-19-guidance-on-reducing-the-risks-in-schools/pages/scientific-and-public-health-advice/#PPE https://www.hps.scot.nhs.uk/web-resources-container/covid-19-contact-tracing-health-protection-team-guidance/ https://www.hps.scot.nhs.uk/web-resources-container/covid-19-guidance-for-health-protection-teams-hpts/

Figure S1 - Vaccination status by occupation

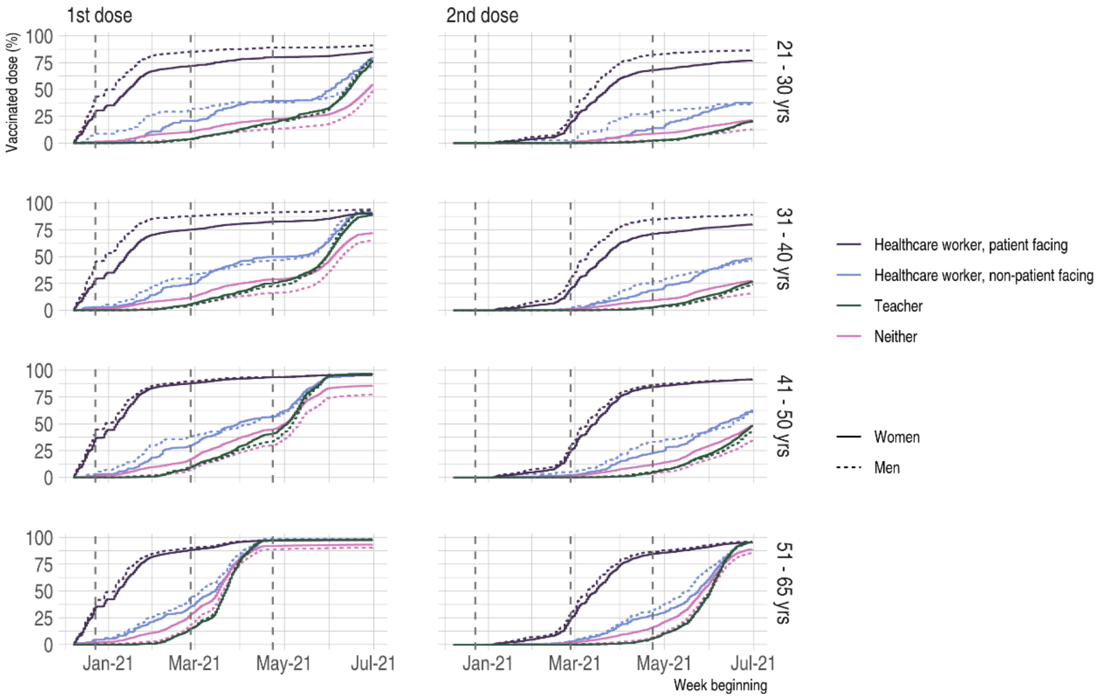

The percentage vaccinated was estimated using the control arm of the case-control study, re-weighting the proportions based on the fact that the control arm represents a stratified random sample of the Scottish population (as described in the main methods section). As for Table 1 in the main manuscript, the probability of selection into the control arm was calculated by age, sex, Scottish Index of Multiple Deprivation (SIMD) and healthboard area of residence and the inverse of this value was used to produce a weighted proportion at each time-point.

Figure S2 - Rate ratios for any case of COVID-19, hospitalisation with COVID-19 and severe COVID-19 having censored events and person-time after any individual had received a first vaccine

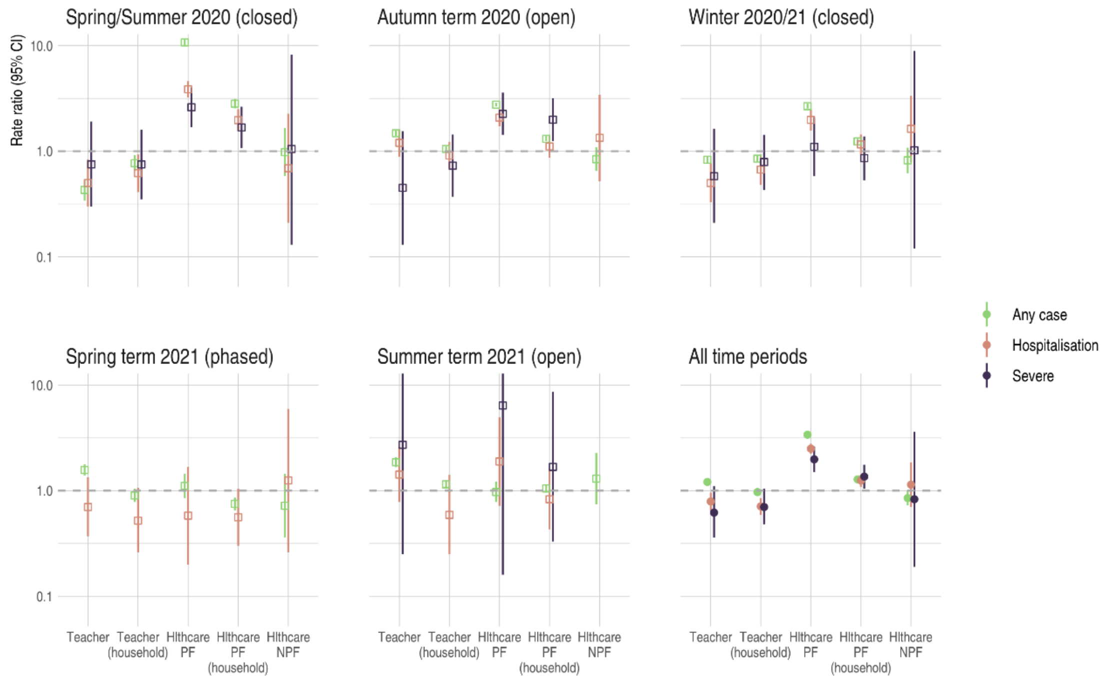

See main manuscript for description of method.

Rates of testing and testing positive

While we had results for all teachers who tested positive (via the case arm of the case control study), we did not have negative testing data for all teachers, only for those in the control arm. As such, we estimated the number of teachers tested as follows. For any teacher selected as a control at any time (a stratified random sample of the population), we excluded all tests occuring after their first positive test. Next we estimated the proportion of unique individuals tested during each calendar week by age and sex. Next we applied these proportions to the denominator teacher dataset to estimate the total number of teachers tested each week within each age and sex defined stratum, then summed this to obtain the total number of teachers tested each week. We repeated this exercise for healthcare workers and the general population. Figure S3-A shows the estimated proportion of each group tested using this approach.

Subsequently, we used the case arm of the study to identify the first positive test of each teacher and summed this by age and sex. We then divided this number by the total number in each group to obtain the test rates (Figure S3-B) and by the estimated number tested in each group to obtain the proportion testing positive of all those tested (Figure S3-C) for each week.

Figure S3-D shows the rate ratio for any case of COVID-19. This was obtained directly from the case-control study using the same definition as in the main manuscript via the same analysis - conditional logisitic regression. Unlike the main analysis, cases who did not have a positive case and their matched controls (ie

those defined solely on the basis of a hospital discharge diagnostic code or death) were excluded from the comparison for comprability with the other data shown in the panel plot (Figure S3) which relates to positive tests. Consistent with the fact that this was a working-age population only 595 cases were thus excluded.

Figure S3-E shows a formal comparison of the metrics plotted in Fig S3-C - the proportion testing positive of all those tested in a given week. This comparison was conducted entirely wihtin the case-control dataset and does not rely on estimating test rates based on the teacher (or healthcare worker) denominator datasets, hence it also includes a comparison of household members of teachers and healthcare workers. This comparison was implemented as follows.

First we restricted the case-control dataset to cases and controls who had been tested. The original stratification (by age in single years, sex and GP practice) would have led to too few strata with at least one case or control tested contemporaneously, therefore we created new strata based on a combination of week of testing and health board area of residence (there are 14 geographic health boards in Scotland). Effect estimates for teachers and healthcare workers were then obtained by fitting a generalised linear model with a logit link and binomial likelihood conditioning on these new stratifying variables (this has previously been shown to be mathematically identical to fitting a conditional logistic regression model although it is less computationally efficient in some circumstances) as well as on the other potential confounders and matching variables included in the main analysis. Figure S3-E shows the results of fitting this model in rolling 3-week periods from March to January 2021.

Healthcare workers were much more likely to be tested for SARS-CoV-2, while the number of teachers tested each week for SARS-CoV-2 was similar to the numbers of individuals in the general population except in late August and late December when there were short spikes in testing among teachers (Figure S3-A). Relative to the general population, the proportion of teachers testing positive (of those tested) increased more rapidly in September before gradually declining towards (but not reaching) the population average in the period from mid-September until the end of follow-up (Figures S3-C and S3-E). Household members of teachers were more similar to the general population (Figure S3-E).

The proportion testing positive in healthcare workers was less than that of the general population. This is paradoxical given the higher rates of severe COVID-19 and hospitalisation with COVID-19 in this group. The finding is too early to be explained by vaccination and the most plausible explanation is that the pattern of testing is different among healthcare workers, with a higher proportion of tests being screening tests than the other groups. We do not know the extent to which different patterns in testing account for differences observed in the proportion testing positive for teachers, household members of teachers, or household members of healthcare workers.

Figure S3 Testing, test positives and test positives as a proportion of those tested over time

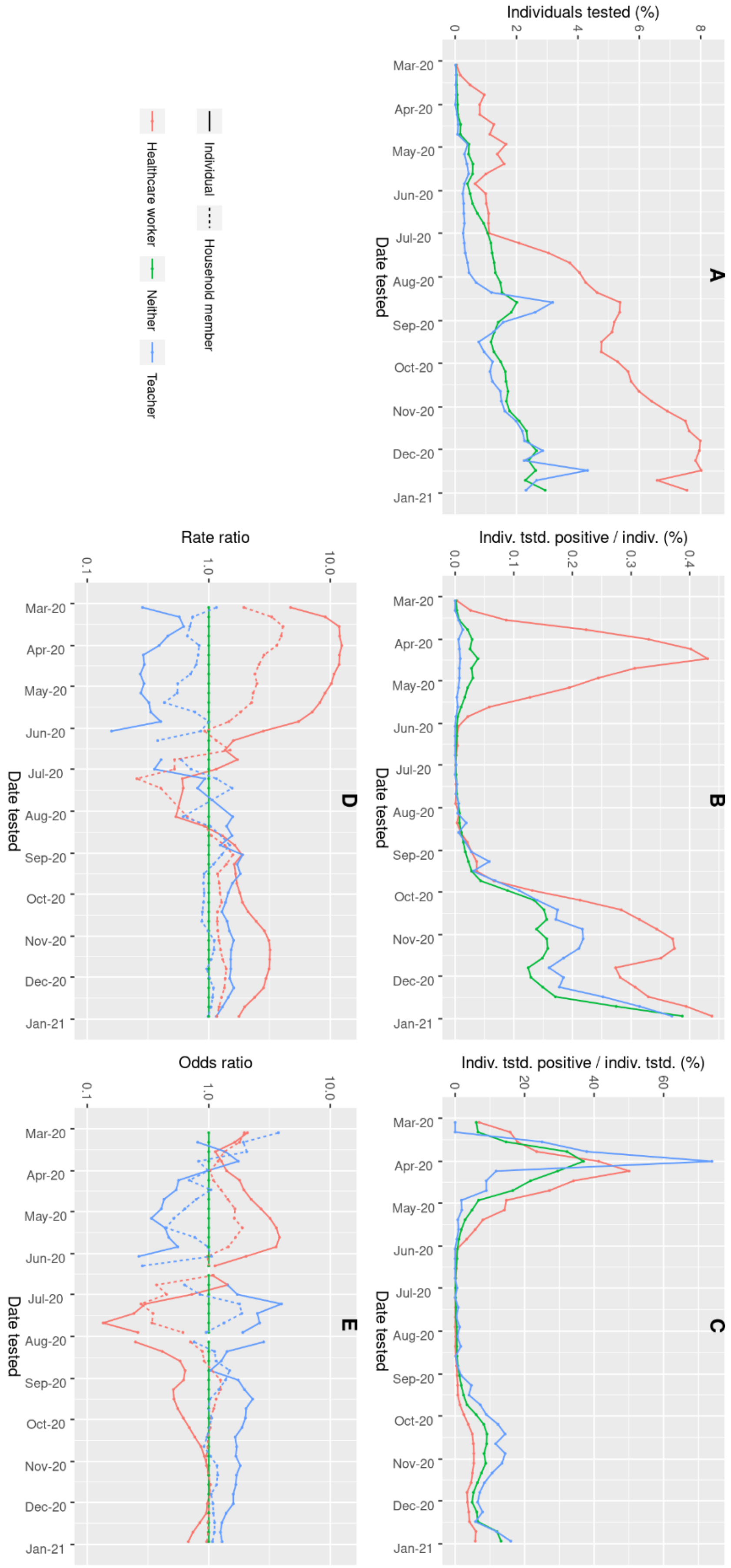

Counts of events

Table S1 - Counts of events by age, sex, time period and number of individuals in each group. Counts are mutually exclusive

Sex	Time period	Age group (years)	Group	Controls	Cases only	Hospitalised or severe
Men	Spring/Summer 2020 (closed)	21 - 30	Healthcare worker, non-patient facing	1	0	0
Men	Spring/Summer 2020 (closed)	21 - 30	Healthcare worker, patient facing	35	86	1
Men	Spring/Summer 2020 (closed)	21 - 30	Household member of healthcare worker	120	49	8
Men	Spring/Summer 2020 (closed)	21 - 30	Household member of teacher	94	8	0
Men	Spring/Summer 2020 (closed)	21 - 30	Neither	3097	444	38
Men	Spring/Summer 2020 (closed)	21 - 30	Teacher	15	3	0
Men	Autumn term 2020 (open)	21 - 30	Healthcare worker, non-patient facing	19	2	0
Men	Autumn term 2020 (open)	21 - 30	Healthcare worker, patient facing	141	101	5
Men	Autumn term 2020 (open)	21 - 30	Household member of healthcare worker	1083	233	3
Men	Autumn term 2020 (open)	21 - 30	Household member of teacher	839	183	1
Men	Autumn term 2020 (open)	21 - 30	Neither	27879	4480	75
Men	Autumn term 2020 (open)	21 - 30	Teacher	136	50	1
Men	Winter 2020/21 (closed)	21 - 30	Healthcare worker, non-patient facing	15	4	0
Men	Winter 2020/21 (closed)	21 - 30	Healthcare worker, patient facing	215	69	2
Men	Winter 2020/21 (closed)	21 - 30	Household member of healthcare worker	1507	306	1
Men	Winter 2020/21 (closed)	21 - 30	Household member of teacher	1027	163	1
Men	Winter 2020/21 (closed)	21 - 30	Neither	39338	5793	100
Men	Winter 2020/21 (closed)	21 - 30	Teacher	211	24	0
Men	Spring term 2021 (phased)	21 - 30	Healthcare worker, non-patient facing	2	0	0
Men	Spring term 2021 (phased)	21 - 30	Healthcare worker, patient facing	48	8	0
Men	Spring term 2021 (phased)	21 - 30	Household member of healthcare worker	344	61	2
Men	Spring term 2021 (phased)	21 - 30	Household member of teacher	228	34	2
Men	Spring term 2021 (phased)	21 - 30	Neither	9193	1581	46
Men	Spring term 2021 (phased)	21 - 30	Teacher	47	7	0

Sex	Time period	Age group (years)	Group	Controls	Cases only	Hospitalised or severe
Men	Summer term 2021 (open)	21 - 30	Healthcare worker, non-patient facing	29	2	0
Men	Summer term 2021 (open)	21 - 30	Healthcare worker, patient facing	273	20	0
Men	Summer term 2021 (open)	21 - 30	Household member of healthcare worker	2304	402	5
Men	Summer term 2021 (open)	21 - 30	Household member of teacher	1632	323	4
Men	Summer term 2021 (open)	21 - 30	Neither	59808	8343	104
Men	Summer term 2021 (open)	21 - 30	Teacher	263	64	1
Women	Spring/Summer 2020 (closed)	21 - 30	Healthcare worker, non-patient facing	5	0	0
Women	Spring/Summer 2020 (closed)	21 - 30	Healthcare worker, patient facing	222	402	14
Women	Spring/Summer 2020 (closed)	21 - 30	Household member of healthcare worker	163	50	4
Women	Spring/Summer 2020 (closed)	21 - 30	Household member of teacher	125	12	0
Women	Spring/Summer 2020 (closed)	21 - 30	Neither	6127	820	77
Women	Spring/Summer 2020 (closed)	21 - 30	Teacher	168	8	1
Women	Autumn term 2020 (open)	21 - 30	Healthcare worker, non-patient facing	37	5	1
Women	Autumn term 2020 (open)	21 - 30	Healthcare worker, patient facing	1206	604	7
Women	Autumn term 2020 (open)	21 - 30	Household member of healthcare worker	1025	235	4
Women	Autumn term 2020 (open)	21 - 30	Household member of teacher	766	153	4
Women	Autumn term 2020 (open)	21 - 30	Neither	37144	5813	138
Women	Autumn term 2020 (open)	21 - 30	Teacher	918	221	6
Women	Winter 2020/21 (closed)	21 - 30	Healthcare worker, non-patient facing	44	6	3
Women	Winter 2020/21 (closed)	21 - 30	Healthcare worker, patient facing	1386	493	10
Women	Winter 2020/21 (closed)	21 - 30	Household member of healthcare worker	1268	241	7
Women	Winter 2020/21 (closed)	21 - 30	Household member of teacher	832	121	2
Women	Winter 2020/21 (closed)	21 - 30	Neither	44501	6355	174
Women	Winter 2020/21 (closed)	21 - 30	Teacher	1159	151	2
Women	Spring term 2021 (phased)	21 - 30	Healthcare worker, non-patient facing	10	0	0
Women	Spring term 2021 (phased)	21 - 30	Healthcare worker, patient facing	294	36	2

Sex	Time period	Age group (years)	Group	Controls	Cases only	Hospitalised or severe
Women	Spring term 2021 (phased)	21 - 30	Household member of healthcare worker	238	21	2
Women	Spring term 2021 (phased)	21 - 30	Household member of teacher	186	23	0
Women	Spring term 2021 (phased)	21 - 30	Neither	9506	1715	82
Women	Spring term 2021 (phased)	21 - 30	Teacher	218	68	2
Women	Summer term 2021 (open)	21 - 30	Healthcare worker, non-patient facing	35	5	0
Women	Summer term 2021 (open)	21 - 30	Healthcare worker, patient facing	1163	90	4
Women	Summer term 2021 (open)	21 - 30	Household member of healthcare worker	1241	206	4
Women	Summer term 2021 (open)	21 - 30	Household member of teacher	924	163	1
Women	Summer term 2021 (open)	21 - 30	Neither	44182	5905	144
Women	Summer term 2021 (open)	21 - 30	Teacher	983	265	3
Men	Spring/Summer 2020 (closed)	31 - 40	Healthcare worker, non-patient facing	6	1	1
Men	Spring/Summer 2020 (closed)	31 - 40	Healthcare worker, patient facing	49	110	5
Men	Spring/Summer 2020 (closed)	31 - 40	Household member of healthcare worker	140	56	7
Men	Spring/Summer 2020 (closed)	31 - 40	Household member of teacher	144	12	1
Men	Spring/Summer 2020 (closed)	31 - 40	Neither	4353	419	97
Men	Spring/Summer 2020 (closed)	31 - 40	Teacher	57	3	0
Men	Autumn term 2020 (open)	31 - 40	Healthcare worker, non-patient facing	39	6	1
Men	Autumn term 2020 (open)	31 - 40	Healthcare worker, patient facing	277	140	5
Men	Autumn term 2020 (open)	31 - 40	Household member of healthcare worker	938	209	2
Men	Autumn term 2020 (open)	31 - 40	Household member of teacher	967	161	3
Men	Autumn term 2020 (open)	31 - 40	Neither	31665	4139	158
Men	Autumn term 2020 (open)	31 - 40	Teacher	324	102	2
Men	Winter 2020/21 (closed)	31 - 40	Healthcare worker, non-patient facing	60	7	1
Men	Winter 2020/21 (closed)	31 - 40	Healthcare worker, patient facing	372	98	5
Men	Winter 2020/21 (closed)	31 - 40	Household member of healthcare worker	1277	238	10
Men	Winter 2020/21 (closed)	31 - 40	Household member of teacher	1183	164	3

Sex	Time period	Age group (years)	Group	Controls	Cases only	Hospitalised or severe
Men	Winter 2020/21 (closed)	31 - 40	Neither	40868	5166	209
Men	Winter 2020/21 (closed)	31 - 40	Teacher	411	43	2
Men	Spring term 2021 (phased)	31 - 40	Healthcare worker, non-patient facing	9	1	0
Men	Spring term 2021 (phased)	31 - 40	Healthcare worker, patient facing	82	4	0
Men	Spring term 2021 (phased)	31 - 40	Household member of healthcare worker	369	52	3
Men	Spring term 2021 (phased)	31 - 40	Household member of teacher	344	52	0
Men	Spring term 2021 (phased)	31 - 40	Neither	12079	1726	105
Men	Spring term 2021 (phased)	31 - 40	Teacher	111	25	0
Men	Summer term 2021 (open)	31 - 40	Healthcare worker, non-patient facing	42	3	0
Men	Summer term 2021 (open)	31 - 40	Healthcare worker, patient facing	339	15	0
Men	Summer term 2021 (open)	31 - 40	Household member of healthcare worker	1079	154	5
Men	Summer term 2021 (open)	31 - 40	Household member of teacher	941	159	1
Men	Summer term 2021 (open)	31 - 40	Neither	33940	4077	157
Men	Summer term 2021 (open)	31 - 40	Teacher	335	80	2
Women	Spring/Summer 2020 (closed)	31 - 40	Healthcare worker, non-patient facing	18	1	0
Women	Spring/Summer 2020 (closed)	31 - 40	Healthcare worker, patient facing	372	439	17
Women	Spring/Summer 2020 (closed)	31 - 40	Household member of healthcare worker	92	16	1
Women	Spring/Summer 2020 (closed)	31 - 40	Household member of teacher	58	4	0
Women	Spring/Summer 2020 (closed)	31 - 40	Neither	7277	772	131
Women	Spring/Summer 2020 (closed)	31 - 40	Teacher	302	15	5
Women	Autumn term 2020 (open)	31 - 40	Healthcare worker, non-patient facing	100	4	0
Women	Autumn term 2020 (open)	31 - 40	Healthcare worker, patient facing	1904	749	28
Women	Autumn term 2020 (open)	31 - 40	Household member of healthcare worker	420	60	3
Women	Autumn term 2020 (open)	31 - 40	Household member of teacher	379	40	0
Women	Autumn term 2020 (open)	31 - 40	Neither	40070	5581	224
Women	Autumn term 2020 (open)	31 - 40	Teacher	1792	332	14

Sex	Time period	Age group (years)	Group	Controls	Cases only	Hospitalised or severe
Women	Winter 2020/21 (closed)	31 - 40	Healthcare worker, non-patient facing	106	9	0
Women	Winter 2020/21 (closed)	31 - 40	Healthcare worker, patient facing	2071	567	21
Women	Winter 2020/21 (closed)	31 - 40	Household member of healthcare worker	476	71	1
Women	Winter 2020/21 (closed)	31 - 40	Household member of teacher	407	41	3
Women	Winter 2020/21 (closed)	31 - 40	Neither	43261	5722	230
Women	Winter 2020/21 (closed)	31 - 40	Teacher	1840	245	10
Women	Spring term 2021 (phased)	31 - 40	Healthcare worker, non-patient facing	33	3	1
Women	Spring term 2021 (phased)	31 - 40	Healthcare worker, patient facing	494	47	1
Women	Spring term 2021 (phased)	31 - 40	Household member of healthcare worker	132	8	0
Women	Spring term 2021 (phased)	31 - 40	Household member of teacher	95	14	0
Women	Spring term 2021 (phased)	31 - 40	Neither	11316	1760	127
Women	Spring term 2021 (phased)	31 - 40	Teacher	487	111	5
Women	Summer term 2021 (open)	31 - 40	Healthcare worker, non-patient facing	79	6	0
Women	Summer term 2021 (open)	31 - 40	Healthcare worker, patient facing	1322	122	9
Women	Summer term 2021 (open)	31 - 40	Household member of healthcare worker	291	29	1
Women	Summer term 2021 (open)	31 - 40	Household member of teacher	262	29	0
Women	Summer term 2021 (open)	31 - 40	Neither	28560	3710	203
Women	Summer term 2021 (open)	31 - 40	Teacher	1223	271	7
Men	Spring/Summer 2020 (closed)	41 - 50	Healthcare worker, non-patient facing	11	1	0
Men	Spring/Summer 2020 (closed)	41 - 50	Healthcare worker, patient facing	62	135	22
Men	Spring/Summer 2020 (closed)	41 - 50	Household member of healthcare worker	159	62	17
Men	Spring/Summer 2020 (closed)	41 - 50	Household member of teacher	134	9	2
Men	Spring/Summer 2020 (closed)	41 - 50	Neither	5159	327	217
Men	Spring/Summer 2020 (closed)	41 - 50	Teacher	50	6	1
Men	Autumn term 2020 (open)	41 - 50	Healthcare worker, non-patient facing	57	7	0
Men	Autumn term 2020 (open)	41 - 50	Healthcare worker, patient facing	391	174	8

Sex	Time period	Age group (years)	Group	Controls	Cases only	Hospitalised or severe
Men	Autumn term 2020 (open)	41 - 50	Household member of healthcare worker	935	202	8
Men	Autumn term 2020 (open)	41 - 50	Household member of teacher	953	155	9
Men	Autumn term 2020 (open)	41 - 50	Neither	31596	3846	287
Men	Autumn term 2020 (open)	41 - 50	Teacher	287	92	5
Men	Winter 2020/21 (closed)	41 - 50	Healthcare worker, non-patient facing	76	4	0
Men	Winter 2020/21 (closed)	41 - 50	Healthcare worker, patient facing	388	96	5
Men	Winter 2020/21 (closed)	41 - 50	Household member of healthcare worker	1053	180	9
Men	Winter 2020/21 (closed)	41 - 50	Household member of teacher	1027	106	5
Men	Winter 2020/21 (closed)	41 - 50	Neither	35532	4212	310
Men	Winter 2020/21 (closed)	41 - 50	Teacher	309	28	0
Men	Spring term 2021 (phased)	41 - 50	Healthcare worker, non-patient facing	14	0	0
Men	Spring term 2021 (phased)	41 - 50	Healthcare worker, patient facing	109	11	0
Men	Spring term 2021 (phased)	41 - 50	Household member of healthcare worker	308	39	2
Men	Spring term 2021 (phased)	41 - 50	Household member of teacher	253	57	3
Men	Spring term 2021 (phased)	41 - 50	Neither	9490	1223	116
Men	Spring term 2021 (phased)	41 - 50	Teacher	76	17	0
Men	Summer term 2021 (open)	41 - 50	Healthcare worker, non-patient facing	57	2	0
Men	Summer term 2021 (open)	41 - 50	Healthcare worker, patient facing	313	19	0
Men	Summer term 2021 (open)	41 - 50	Household member of healthcare worker	709	107	1
Men	Summer term 2021 (open)	41 - 50	Household member of teacher	697	128	0
Men	Summer term 2021 (open)	41 - 50	Neither	24118	2811	153
Men	Summer term 2021 (open)	41 - 50	Teacher	250	58	3
Women	Spring/Summer 2020 (closed)	41 - 50	Healthcare worker, non-patient facing	38	4	0
Women	Spring/Summer 2020 (closed)	41 - 50	Healthcare worker, patient facing	474	450	44
Women	Spring/Summer 2020 (closed)	41 - 50	Household member of healthcare worker	90	36	2
Women	Spring/Summer 2020 (closed)	41 - 50	Household member of teacher	81	4	1

Sex	Time period	Age group (years)	Group	Controls	Cases only	Hospitalised or severe
Women	Spring/Summer 2020 (closed)	41 - 50	Neither	8472	826	189
Women	Spring/Summer 2020 (closed)	41 - 50	Teacher	342	9	4
Women	Autumn term 2020 (open)	41 - 50	Healthcare worker, non-patient facing	147	14	0
Women	Autumn term 2020 (open)	41 - 50	Healthcare worker, patient facing	1902	714	30
Women	Autumn term 2020 (open)	41 - 50	Household member of healthcare worker	419	77	3
Women	Autumn term 2020 (open)	41 - 50	Household member of teacher	294	42	1
Women	Autumn term 2020 (open)	41 - 50	Neither	37907	5068	275
Women	Autumn term 2020 (open)	41 - 50	Teacher	1518	275	9
Women	Winter 2020/21 (closed)	41 - 50	Healthcare worker, non-patient facing	139	6	3
Women	Winter 2020/21 (closed)	41 - 50	Healthcare worker, patient facing	1966	536	17
Women	Winter 2020/21 (closed)	41 - 50	Household member of healthcare worker	388	62	4
Women	Winter 2020/21 (closed)	41 - 50	Household member of teacher	326	29	4
Women	Winter 2020/21 (closed)	41 - 50	Neither	38628	4904	306
Women	Winter 2020/21 (closed)	41 - 50	Teacher	1477	132	5
Women	Spring term 2021 (phased)	41 - 50	Healthcare worker, non-patient facing	26	3	1
Women	Spring term 2021 (phased)	41 - 50	Healthcare worker, patient facing	462	43	3
Women	Spring term 2021 (phased)	41 - 50	Household member of healthcare worker	80	12	2
Women	Spring term 2021 (phased)	41 - 50	Household member of teacher	72	10	1
Women	Spring term 2021 (phased)	41 - 50	Neither	8736	1303	100
Women	Spring term 2021 (phased)	41 - 50	Teacher	363	86	1
Women	Summer term 2021 (open)	41 - 50	Healthcare worker, non-patient facing	86	7	0
Women	Summer term 2021 (open)	41 - 50	Healthcare worker, patient facing	1091	100	4
Women	Summer term 2021 (open)	41 - 50	Household member of healthcare worker	235	17	0
Women	Summer term 2021 (open)	41 - 50	Household member of teacher	177	22	1
Women	Summer term 2021 (open)	41 - 50	Neither	21868	2748	152
Women	Summer term 2021 (open)	41 - 50	Teacher	940	196	3

Sex	Time period	Age group (years)	Group	Controls	Cases only	Hospitalised or severe
Men	Spring/Summer 2020 (closed)	51 - 65	Healthcare worker, non-patient facing	19	1	0
Men	Spring/Summer 2020 (closed)	51 - 65	Healthcare worker, patient facing	171	180	26
Men	Spring/Summer 2020 (closed)	51 - 65	Household member of healthcare worker	416	98	48
Men	Spring/Summer 2020 (closed)	51 - 65	Household member of teacher	315	24	13
Men	Spring/Summer 2020 (closed)	51 - 65	Neither	12574	671	810
Men	Spring/Summer 2020 (closed)	51 - 65	Teacher	82	4	3
Men	Autumn term 2020 (open)	51 - 65	Healthcare worker, non-patient facing	83	5	1
Men	Autumn term 2020 (open)	51 - 65	Healthcare worker, patient facing	599	255	25
Men	Autumn term 2020 (open)	51 - 65	Household member of healthcare worker	1623	371	34
Men	Autumn term 2020 (open)	51 - 65	Household member of teacher	1337	221	21
Men	Autumn term 2020 (open)	51 - 65	Neither	54268	5952	1020
Men	Autumn term 2020 (open)	51 - 65	Teacher	361	69	5
Men	Winter 2020/21 (closed)	51 - 65	Healthcare worker, non-patient facing	103	5	0
Men	Winter 2020/21 (closed)	51 - 65	Healthcare worker, patient facing	675	207	15
Men	Winter 2020/21 (closed)	51 - 65	Household member of healthcare worker	1844	350	47
Men	Winter 2020/21 (closed)	51 - 65	Household member of teacher	1465	158	18
Men	Winter 2020/21 (closed)	51 - 65	Neither	59012	6191	1112
Men	Winter 2020/21 (closed)	51 - 65	Teacher	419	32	1
Men	Spring term 2021 (phased)	51 - 65	Healthcare worker, non-patient facing	23	1	0
Men	Spring term 2021 (phased)	51 - 65	Healthcare worker, patient facing	138	13	0
Men	Spring term 2021 (phased)	51 - 65	Household member of healthcare worker	328	45	2
Men	Spring term 2021 (phased)	51 - 65	Household member of teacher	273	32	1
Men	Spring term 2021 (phased)	51 - 65	Neither	10926	1311	247
Men	Spring term 2021 (phased)	51 - 65	Teacher	76	12	2
Men	Summer term 2021 (open)	51 - 65	Healthcare worker, non-patient facing	42	3	0
Men	Summer term 2021 (open)	51 - 65	Healthcare worker, patient facing	315	22	1

Sex	Time period	Age group (years)	Group	Controls	Cases only	Hospitalised or severe
Men	Summer term 2021 (open)	51 - 65	Household member of healthcare worker	723	101	4
Men	Summer term 2021 (open)	51 - 65	Household member of teacher	554	94	0
Men	Summer term 2021 (open)	51 - 65	Neither	21976	2494	173
Men	Summer term 2021 (open)	51 - 65	Teacher	153	37	0
Women	Spring/Summer 2020 (closed)	51 - 65	Healthcare worker, non-patient facing	45	4	2
Women	Spring/Summer 2020 (closed)	51 - 65	Healthcare worker, patient facing	782	633	75
Women	Spring/Summer 2020 (closed)	51 - 65	Household member of healthcare worker	253	69	9
Women	Spring/Summer 2020 (closed)	51 - 65	Household member of teacher	202	9	8
Women	Spring/Summer 2020 (closed)	51 - 65	Neither	17187	1368	647
Women	Spring/Summer 2020 (closed)	51 - 65	Teacher	408	8	2
Women	Autumn term 2020 (open)	51 - 65	Healthcare worker, non-patient facing	189	16	2
Women	Autumn term 2020 (open)	51 - 65	Healthcare worker, patient facing	2724	906	52
Women	Autumn term 2020 (open)	51 - 65	Household member of healthcare worker	819	160	19
Women	Autumn term 2020 (open)	51 - 65	Household member of teacher	834	121	10
Women	Autumn term 2020 (open)	51 - 65	Neither	61612	7356	806
Women	Autumn term 2020 (open)	51 - 65	Teacher	1419	228	12
Women	Winter 2020/21 (closed)	51 - 65	Healthcare worker, non-patient facing	184	7	2
Women	Winter 2020/21 (closed)	51 - 65	Healthcare worker, patient facing	2851	749	31
Women	Winter 2020/21 (closed)	51 - 65	Household member of healthcare worker	794	149	19
Women	Winter 2020/21 (closed)	51 - 65	Household member of teacher	821	89	6
Women	Winter 2020/21 (closed)	51 - 65	Neither	64511	7158	926
Women	Winter 2020/21 (closed)	51 - 65	Teacher	1520	134	4
Women	Spring term 2021 (phased)	51 - 65	Healthcare worker, non-patient facing	41	3	1
Women	Spring term 2021 (phased)	51 - 65	Healthcare worker, patient facing	460	49	3
Women	Spring term 2021 (phased)	51 - 65	Household member of healthcare worker	148	16	1
Women	Spring term 2021 (phased)	51 - 65	Household member of teacher	128	19	3

Sex	Time period	Age group (years)	Group	Controls	Cases only	Hospitalised or severe
Women	Spring term 2021 (phased)	51 - 65	Neither	10213	1339	209
Women	Spring term 2021 (phased)	51 - 65	Teacher	237	45	3
Women	Summer term 2021 (open)	51 - 65	Healthcare worker, non-patient facing	84	6	0
Women	Summer term 2021 (open)	51 - 65	Healthcare worker, patient facing	905	95	5
Women	Summer term 2021 (open)	51 - 65	Household member of healthcare worker	294	21	0
Women	Summer term 2021 (open)	51 - 65	Household member of teacher	253	50	1
Women	Summer term 2021 (open)	51 - 65	Neither	19577	2349	176
Women	Summer term 2021 (open)	51 - 65	Teacher	484	110	2

Effect measure estimates

The following tables display the same effect estimates as shown Tables 2 and 3 of the main manuscript but broken down into age and sex strata (Table S2a and S2b), additional time periods (Tables S3a and S3b) or age and sex strata and additional time periods (Tables S4a and S4b). Please see footnotes of Tables 2 and 3 of the main manuscript for additional details.

Table S2a - Rate ratios for any case, hospitalisation with COVID-19 and severe COVID-19 for teachers, healthcare workers and members of their households, stratified by time period

Adjustment	Time period	Outcome	Neither	Healthcare worker, patient facing	Household member of healthcare worker	Household member of teacher	Teacher	Healthcare worker, non-patient facing
Unadjusted	Spring/Summer 2020 (closed)	Any case	1	10.45 (9.83-11.11)	2.95 (2.67-3.25)	0.77 (0.65-0.92)	0.41 (0.32-0.52)	0.88 (0.52-1.48)
Adjusted	Spring/Summer 2020 (closed)	Any case	1	10.71 (10.06-11.40)	2.82 (2.55-3.12)	0.77 (0.65-0.92)	0.43 (0.34-0.55)	0.97 (0.57-1.65)
Unadjusted	Spring/Summer 2020 (closed)	Hospitalisation	1	3.49 (2.94-4.13)	1.96 (1.57-2.46)	0.58 (0.39-0.87)	0.44 (0.27-0.74)	0.62 (0.19-2.01)
Adjusted	Spring/Summer 2020 (closed)	Hospitalisation	1	3.73 (3.12-4.45)	1.96 (1.54-2.49)	0.62 (0.42-0.94)	0.49 (0.29-0.82)	0.68 (0.21-2.21)
Unadjusted	Spring/Summer 2020 (closed)	Severe	1	2.30 (1.52-3.47)	1.77 (1.16-2.72)	0.70 (0.34-1.45)	0.56 (0.23-1.40)	0.74 (0.10-5.66)
Adjusted	Spring/Summer 2020 (closed)	Severe	1	2.49 (1.62-3.84)	1.71 (1.09-2.68)	0.75 (0.35-1.61)	0.71 (0.28-1.80)	1.02 (0.13-7.94)
Unadjusted	Autumn term 2020 (open)	Any case	1	2.76 (2.66-2.87)	1.51 (1.44-1.60)	1.20 (1.13-1.27)	1.48 (1.40-1.57)	0.70 (0.55-0.91)
Adjusted	Autumn term 2020 (open)	Any case	1	2.76 (2.66-2.87)	1.31 (1.25-1.39)	1.05 (0.98-1.11)	1.48 (1.40-1.57)	0.84 (0.65-1.09)
Unadjusted	Autumn term 2020 (open)	Hospitalisation	1	1.97 (1.65-2.35)	1.21 (0.95-1.54)	0.90 (0.67-1.21)	1.09 (0.82-1.46)	1.02 (0.41-2.57)
Adjusted	Autumn term 2020 (open)	Hospitalisation	1	2.07 (1.72-2.49)	1.13 (0.88-1.45)	0.92 (0.68-1.24)	1.19 (0.88-1.60)	1.32 (0.51-3.39)
Unadjusted	Autumn term 2020 (open)	Severe	1	1.88 (1.21-2.91)	1.97 (1.28-3.03)	0.70 (0.36-1.34)	0.49 (0.15-1.58)	-
Adjusted	Autumn term 2020 (open)	Severe	1	2.38 (1.50-3.78)	2.03 (1.28-3.23)	0.73 (0.37-1.44)	0.40 (0.11-1.42)	-
Unadjusted	Winter 2020/21 (closed)	Any case	1	2.07 (1.99-2.16)	1.42 (1.35-1.50)	0.95 (0.89-1.01)	0.81 (0.76-0.88)	0.61 (0.47-0.80)
Adjusted	Winter 2020/21 (closed)	Any case	1	2.09 (2.01-2.18)	1.23 (1.17-1.30)	0.84 (0.78-0.90)	0.83 (0.77-0.90)	0.75 (0.57-0.99)

Adjustment	Time period	Outcome	Neither	Healthcare worker, patient facing	Household member of healthcare worker	Household member of teacher	Teacher	Healthcare worker, non-patient facing
Unadjusted	Winter 2020/21 (closed)	Hospitalisation	1	1.11 (0.90-1.37)	1.31 (1.06-1.61)	0.68 (0.50-0.94)	0.42 (0.28-0.63)	1.22 (0.61-2.46)
Adjusted	Winter 2020/21 (closed)	Hospitalisation	1	1.10 (0.89-1.36)	1.13 (0.91-1.41)	0.64 (0.46-0.89)	0.51 (0.33-0.77)	1.49 (0.73-3.06)
Unadjusted	Winter 2020/21 (closed)	Severe	1	0.54 (0.29-1.00)	1.03 (0.66-1.62)	0.87 (0.49-1.55)	0.41 (0.15-1.12)	0.85 (0.11-6.72)
Adjusted	Winter 2020/21 (closed)	Severe	1	0.63 (0.34-1.17)	0.86 (0.53-1.37)	0.79 (0.43-1.43)	0.56 (0.20-1.56)	1.05 (0.12-8.95)
Unadjusted	Spring term 2021 (phased)	Any case	1	0.60 (0.52-0.69)	0.84 (0.74-0.95)	1.01 (0.89-1.15)	1.48 (1.32-1.65)	0.55 (0.32-0.94)
Adjusted	Spring term 2021 (phased)	Any case	1	0.59 (0.52-0.68)	0.72 (0.64-0.82)	0.87 (0.76-0.99)	1.57 (1.40-1.76)	0.68 (0.39-1.17)
Unadjusted	Spring term 2021 (phased)	Hospitalisation	1	0.32 (0.16-0.63)	0.56 (0.32-0.96)	0.52 (0.28-1.00)	0.67 (0.38-1.18)	0.90 (0.27-2.97)
Adjusted	Spring term 2021 (phased)	Hospitalisation	1	0.34 (0.17-0.66)	0.54 (0.31-0.94)	0.53 (0.28-1.01)	0.71 (0.40-1.27)	0.93 (0.27-3.14)
Unadjusted	Spring term 2021 (phased)	Severe	1	-	0.30 (0.04-2.21)	-	0.57 (0.13-2.41)	-
Adjusted	Spring term 2021 (phased)	Severe	1	-	0.26 (0.03-1.97)	-	0.57 (0.13-2.54)	-
Unadjusted	Summer term 2021 (open)	Any case	1	0.61 (0.55-0.67)	1.09 (1.02-1.16)	1.30 (1.22-1.39)	1.77 (1.65-1.89)	0.56 (0.40-0.79)
Adjusted	Summer term 2021 (open)	Any case	1	0.59 (0.54-0.65)	0.94 (0.88-1.00)	1.10 (1.03-1.18)	1.69 (1.58-1.81)	0.69 (0.49-0.97)
Unadjusted	Summer term 2021 (open)	Hospitalisation	1	0.55 (0.35-0.85)	0.80 (0.51-1.25)	0.33 (0.16-0.68)	0.77 (0.49-1.21)	-
Adjusted	Summer term 2021 (open)	Hospitalisation	1	0.56 (0.36-0.87)	0.71 (0.45-1.12)	0.33 (0.16-0.67)	0.86 (0.54-1.37)	-

Adjustment	Time period	Outcome	Neither	Healthcare worker, patient facing	Household member of healthcare worker	Household member of teacher	Teacher	Healthcare worker, non-patient facing
Unadjusted	Summer term 2021 (open)	Severe	1	0.23 (0.03-1.70)	1.54 (0.59-4.04)	-	0.26 (0.03-1.91)	-
Adjusted	Summer term 2021 (open)	Severe	1	0.22 (0.03-1.67)	1.49 (0.55-4.08)	-	0.32 (0.04-2.48)	-

Table S2 Rate ratios for any case, hospitalisation with COVID-19 and severe COVID-19 for teachers by sector, stratified by time period

**Adjustment**	**Time period**	**Outcome**	**Household member of primary teacher**	**Neither**	**Household member of secondary teacher**	**Household member other teacher**	**Nursery/Primary or Nursery**	**Primary**	**Secondary**	**Teacher in other sector**
Unadjusted	Spring/Summer 2020 (closed)	Any case	0.67 (0.51-0.88)	1	0.86 (0.67-1.12)	0.85 (0.55-1.33)	0.36 (0.21-0.60)	0.44 (0.27-0.71)	0.29 (0.19-0.46)	0.73 (0.46-1.15)
Adjusted	Spring/Summer 2020 (closed)	Any case	0.67 (0.51-0.88)	1	0.85 (0.66-1.10)	0.87 (0.56-1.36)	0.38 (0.23-0.64)	0.47 (0.29-0.76)	0.30 (0.19-0.47)	0.78 (0.49-1.23)
Unadjusted	Spring/Summer 2020 (closed)	Hospitalisation	0.68 (0.39-1.17)	1	0.60 (0.33-1.08)	0.18 (0.03-1.31)	0.28 (0.07-1.16)	0.38 (0.12-1.19)	0.48 (0.22-1.02)	0.63 (0.23-1.75)
Adjusted	Spring/Summer 2020 (closed)	Hospitalisation	0.73 (0.41-1.29)	1	0.64 (0.35-1.17)	0.20 (0.03-1.44)	0.26 (0.06-1.10)	0.41 (0.13-1.33)	0.55 (0.26-1.20)	0.75 (0.27-2.09)
Unadjusted	Autumn term 2020 (open)	Any case	1.21 (1.11-1.32)	1	1.14 (1.03-1.27)	1.29 (1.10-1.51)	1.32 (1.17-1.50)	1.66 (1.50-1.85)	1.46 (1.33-1.60)	1.47 (1.26-1.71)
Adjusted	Autumn term 2020 (open)	Any case	1.05 (0.96-1.14)	1	1.00 (0.90-1.11)	1.18 (1.01-1.38)	1.33 (1.17-1.51)	1.65 (1.48-1.84)	1.47 (1.34-1.62)	1.46 (1.25-1.70)
Unadjusted	Autumn term 2020 (open)	Hospitalisation	1.00 (0.66-1.51)	1	0.67 (0.39-1.16)	1.14 (0.61-2.14)	0.59 (0.26-1.35)	0.89 (0.46-1.70)	1.25 (0.83-1.89)	1.68 (0.91-3.10)
Adjusted	Autumn term 2020 (open)	Hospitalisation	1.05 (0.68-1.60)	1	0.64 (0.37-1.12)	1.24 (0.64-2.38)	0.63 (0.27-1.47)	0.99 (0.51-1.92)	1.36 (0.88-2.08)	1.80 (0.95-3.43)
Unadjusted	Winter 2020/21 (closed)	Any case	1.03 (0.94-1.13)	1	0.87 (0.78-0.98)	0.87 (0.72-1.04)	0.80 (0.69-0.93)	0.98 (0.86-1.13)	0.74 (0.66-0.84)	0.74 (0.60-0.91)
Adjusted	Winter 2020/21 (closed)	Any case	0.90 (0.82-0.99)	1	0.77 (0.69-0.87)	0.80 (0.67-0.97)	0.81 (0.70-0.94)	1.00 (0.87-1.15)	0.77 (0.68-0.87)	0.76 (0.62-0.93)
Unadjusted	Winter 2020/21 (closed)	Hospitalisation	0.81 (0.53-1.23)	1	0.57 (0.33-1.00)	0.55 (0.22-1.36)	0.66 (0.32-1.35)	0.28 (0.09-0.88)	0.41 (0.22-0.78)	0.30 (0.09-0.95)
Adjusted	Winter 2020/21 (closed)	Hospitalisation	0.77 (0.50-1.19)	1	0.49 (0.28-0.88)	0.61 (0.24-1.52)	0.75 (0.36-1.57)	0.33 (0.10-1.06)	0.51 (0.27-0.97)	0.36 (0.11-1.15)
Unadjusted	Spring term 2021 (phased)	Any case	1.16 (0.97-1.38)	1	0.88 (0.71-1.11)	0.85 (0.59-1.23)	2.00 (1.64-2.45)	2.27 (1.89-2.73)	0.78 (0.62-0.98)	1.15 (0.83-1.60)
Adjusted	Spring term 2021 (phased)	Any case	0.99 (0.83-1.19)	1	0.76 (0.61-0.95)	0.72 (0.50-1.04)	2.15 (1.75-2.64)	2.44 (2.02-2.95)	0.81 (0.64-1.03)	1.18 (0.84-1.64)
Unadjusted	Spring term 2021 (phased)	Hospitalisation	0.93 (0.45-1.94)	1	0.14 (0.02-1.01)	0.30 (0.04-2.20)	1.27 (0.49-3.28)	0.41 (0.10-1.71)	0.63 (0.25-1.56)	0.38 (0.05-2.81)

Adjustment	Time period	Outcome	Household member of primary teacher	Neither	Household member of secondary teacher	Household member other teacher	Nursery/Primary or Nursery	Primary	Secondary	Teacher in other sector
Adjusted	Spring term 2021 (phased)	Hospitalisation	0.94 (0.45-1.97)	1	0.15 (0.02-1.09)	0.27 (0.04-1.98)	1.54 (0.58-4.13)	0.46 (0.11-1.94)	0.62 (0.24-1.56)	0.39 (0.05-2.92)
Unadjusted	Summer term 2021 (open)	Any case	1.48 (1.35-1.63)	1	1.13 (1.00-1.27)	1.16 (0.97-1.39)	1.87 (1.64-2.13)	2.15 (1.89-2.44)	1.66 (1.49-1.84)	1.23 (0.99-1.52)
Adjusted	Summer term 2021 (open)	Any case	1.24 (1.13-1.36)	1	0.96 (0.85-1.08)	1.02 (0.85-1.22)	1.75 (1.54-2.00)	2.08 (1.83-2.36)	1.60 (1.43-1.78)	1.18 (0.95-1.46)
Unadjusted	Summer term 2021 (open)	Hospitalisation	0.42 (0.17-1.05)	1	0.11 (0.02-0.79)	0.67 (0.16-2.82)	0.92 (0.39-2.13)	0.49 (0.15-1.57)	0.92 (0.48-1.78)	0.53 (0.13-2.22)
Adjusted	Summer term 2021 (open)	Hospitalisation	0.40 (0.16-1.01)	1	0.11 (0.02-0.80)	0.66 (0.15-2.87)	0.96 (0.41-2.26)	0.62 (0.19-2.00)	1.05 (0.53-2.06)	0.51 (0.12-2.21)

Table S3a - Rate ratios for any case, hospitalisation with COVID-19 and severe COVID-19 for teachers, healthcare workers and members of their households, stratified by time period, age and sex

Adjustment	Time period	Sex	Age group (years)	Outcome	Neither	Healthcare worker, patient facing	Household member of healthcare worker	Household member of teacher	Teacher	Healthcare worker, non-patient facing
Unadjusted	Spring/Summer 2020 (closed)	Men	21 - 30	Any case	1	22.48 (14.91-33.91)	3.14 (2.31-4.27)	0.54 (0.27-1.07)	0.95 (0.28-3.14)	-
Adjusted	Spring/Summer 2020 (closed)	Men	21 - 30	Any case	1	22.35 (14.73-33.93)	2.85 (2.08-3.90)	0.48 (0.24-0.96)	0.94 (0.28-3.14)	-
Unadjusted	Spring/Summer 2020 (closed)	Men	21 - 30	Hospitalisation	1	-	-	-	-	-
Adjusted	Spring/Summer 2020 (closed)	Men	21 - 30	Hospitalisation	1	-	-	-	-	-
Unadjusted	Spring/Summer 2020 (closed)	Men	31 - 40	Any case	1	22.70 (16.07-32.08)	3.71 (2.75-5.02)	0.74 (0.43-1.28)	0.51 (0.16-1.66)	3.06 (0.63-14.85)
Adjusted	Spring/Summer 2020 (closed)	Men	31 - 40	Any case	1	22.21 (15.62-31.59)	3.54 (2.59-4.82)	0.66 (0.37-1.17)	0.53 (0.16-1.72)	2.95 (0.59-14.79)
Unadjusted	Spring/Summer 2020 (closed)	Men	31 - 40	Hospitalisation	1	7.02 (2.13-23.09)	1.96 (0.85-4.52)	0.25 (0.03-1.83)	0.00 (0.00-88613969364933643599872.00)	5.05 (0.27-93.40)
Adjusted	Spring/Summer 2020 (closed)	Men	31 - 40	Hospitalisation	1	8.94 (2.36-33.92)	1.89 (0.75-4.77)	0.27 (0.03-2.13)	-	9.96 (0.46-215.84)
Unadjusted	Spring/Summer 2020 (closed)	Men	41 - 50	Any case	1	23.99 (17.73-32.46)	4.62 (3.48-6.12)	0.76 (0.43-1.34)	1.19 (0.53-2.66)	0.92 (0.12-7.09)
Adjusted	Spring/Summer 2020 (closed)	Men	41 - 50	Any case	1	23.33 (17.12-31.81)	4.26 (3.18-5.71)	0.69 (0.38-1.22)	1.18 (0.52-2.65)	1.09 (0.14-8.43)
Unadjusted	Spring/Summer 2020 (closed)	Men	41 - 50	Hospitalisation	1	7.03 (4.00-12.35)	3.20 (1.80-5.69)	0.68 (0.26-1.77)	0.38 (0.05-2.90)	-
Adjusted	Spring/Summer 2020 (closed)	Men	41 - 50	Hospitalisation	1	6.69 (3.67-12.19)	3.24 (1.75-5.99)	0.63 (0.23-1.70)	0.42 (0.05-3.34)	-
Unadjusted	Spring/Summer 2020 (closed)	Men	51 - 65	Any case	1	11.00 (8.94-13.54)	3.12 (2.58-3.77)	1.05 (0.75-1.46)	0.61 (0.28-1.32)	0.44 (0.06-3.25)

Adjustment	Time period	Sex	Age group (years)	Outcome	Neither	Healthcare worker, patient facing	Household member of healthcare worker	Household member of teacher	Teacher	Healthcare worker, non-patient facing
Adjusted	Spring/Summer 2020 (closed)	Men	51 - 65	Any case	1	11.06 (8.95-13.67)	2.86 (2.35-3.47)	0.99 (0.70-1.38)	0.65 (0.30-1.41)	0.54 (0.07-4.02)
Unadjusted	Spring/Summer 2020 (closed)	Men	51 - 65	Hospitalisation	1	3.31 (2.11-5.21)	1.94 (1.41-2.69)	0.64 (0.34-1.18)	0.62 (0.19-2.00)	-
Adjusted	Spring/Summer 2020 (closed)	Men	51 - 65	Hospitalisation	1	3.48 (2.17-5.58)	1.85 (1.32-2.60)	0.63 (0.33-1.20)	0.68 (0.21-2.22)	-
Unadjusted	Spring/Summer 2020 (closed)	Women	21 - 30	Any case	1	14.27 (12.03-16.94)	1.96 (1.46-2.65)	0.54 (0.31-0.95)	0.34 (0.18-0.67)	-
Adjusted	Spring/Summer 2020 (closed)	Women	21 - 30	Any case	1	14.56 (12.22-17.35)	1.97 (1.45-2.66)	0.56 (0.32-0.98)	0.37 (0.19-0.73)	-
Unadjusted	Spring/Summer 2020 (closed)	Women	21 - 30	Hospitalisation	1	6.28 (2.98-13.26)	2.09 (0.69-6.35)	-	0.50 (0.07-3.80)	-
Adjusted	Spring/Summer 2020 (closed)	Women	21 - 30	Hospitalisation	1	7.04 (3.03-16.35)	2.77 (0.81-9.51)	-	0.73 (0.09-5.98)	-
Unadjusted	Spring/Summer 2020 (closed)	Women	31 - 40	Any case	1	10.24 (8.82-11.88)	1.60 (0.96-2.67)	0.82 (0.43-1.55)	0.53 (0.34-0.84)	0.55 (0.07-4.06)
Adjusted	Spring/Summer 2020 (closed)	Women	31 - 40	Any case	1	10.63 (9.12-12.39)	1.58 (0.95-2.65)	0.86 (0.45-1.64)	0.55 (0.35-0.88)	0.57 (0.08-4.27)
Unadjusted	Spring/Summer 2020 (closed)	Women	31 - 40	Hospitalisation	1	2.62 (1.48-4.65)	0.62 (0.08-4.74)	-	0.94 (0.37-2.42)	-
Adjusted	Spring/Summer 2020 (closed)	Women	31 - 40	Hospitalisation	1	2.62 (1.45-4.74)	0.66 (0.09-5.11)	-	1.11 (0.42-2.94)	-
Unadjusted	Spring/Summer 2020 (closed)	Women	41 - 50	Any case	1	8.95 (7.79-10.28)	3.56 (2.47-5.13)	0.66 (0.35-1.26)	0.34 (0.19-0.59)	1.03 (0.37-2.86)
Adjusted	Spring/Summer 2020 (closed)	Women	41 - 50	Any case	1	9.37 (8.13-10.80)	3.45 (2.39-4.99)	0.68 (0.36-1.28)	0.37 (0.21-0.65)	1.16 (0.41-3.26)
Unadjusted	Spring/Summer 2020 (closed)	Women	41 - 50	Hospitalisation	1	4.21 (2.85-6.24)	1.47 (0.43-5.03)	0.39 (0.05-3.01)	0.56 (0.20-1.55)	-
Adjusted	Spring/Summer 2020 (closed)	Women	41 - 50	Hospitalisation	1	4.72 (3.11-7.19)	1.02 (0.25-4.17)	0.51 (0.07-3.99)	0.57 (0.20-1.63)	-
Unadjusted	Spring/Summer 2020 (closed)	Women	51 - 65	Any case	1	7.59 (6.80-8.46)	2.64 (2.06-3.39)	0.81 (0.55-1.18)	0.21 (0.11-0.38)	0.94 (0.41-2.18)

Adjustment	Time period	Sex	Age group (years)	Outcome	Neither	Healthcare worker, patient facing	Household member of healthcare worker	Household member of teacher	Teacher	Healthcare worker, non-patient facing
Adjusted	Spring/Summer 2020 (closed)	Women	51 - 65	Any case	1	7.88 (7.06-8.81)	2.58 (2.00-3.32)	0.83 (0.57-1.21)	0.23 (0.12-0.43)	1.08 (0.47-2.49)
Unadjusted	Spring/Summer 2020 (closed)	Women	51 - 65	Hospitalisation	1	2.67 (2.04-3.51)	1.21 (0.62-2.35)	0.88 (0.44-1.77)	0.15 (0.04-0.62)	1.13 (0.26-4.89)
Adjusted	Spring/Summer 2020 (closed)	Women	51 - 65	Hospitalisation	1	2.96 (2.23-3.95)	1.28 (0.64-2.58)	0.95 (0.46-1.95)	0.18 (0.04-0.74)	1.05 (0.24-4.72)
Unadjusted	Autumn term 2020 (open)	Men	21 - 30	Any case	1	4.73 (3.72-6.01)	1.27 (1.10-1.46)	1.30 (1.11-1.52)	2.17 (1.59-2.95)	0.98 (0.23-4.20)
Adjusted	Autumn term 2020 (open)	Men	21 - 30	Any case	1	4.66 (3.66-5.95)	1.16 (1.01-1.33)	1.15 (0.98-1.35)	2.13 (1.56-2.90)	1.14 (0.27-4.92)
Unadjusted	Autumn term 2020 (open)	Men	21 - 30	Hospitalisation	1	-	-	-	-	-
Adjusted	Autumn term 2020 (open)	Men	21 - 30	Hospitalisation	1	-	-	-	-	-
Unadjusted	Autumn term 2020 (open)	Men	31 - 40	Any case	1	3.55 (2.92-4.32)	1.55 (1.34-1.80)	1.17 (1.00-1.38)	2.33 (1.87-2.89)	1.42 (0.64-3.16)
Adjusted	Autumn term 2020 (open)	Men	31 - 40	Any case	1	3.45 (2.83-4.20)	1.40 (1.21-1.62)	1.07 (0.91-1.27)	2.29 (1.83-2.86)	1.70 (0.76-3.79)
Unadjusted	Autumn term 2020 (open)	Men	31 - 40	Hospitalisation	1	11.68 (3.11-43.91)	0.34 (0.08-1.40)	0.64 (0.19-2.12)	0.83 (0.19-3.56)	5.54 (0.48-63.70)
Adjusted	Autumn term 2020 (open)	Men	31 - 40	Hospitalisation	1	11.86 (2.60-54.03)	0.32 (0.07-1.43)	0.66 (0.19-2.26)	1.00 (0.22-4.64)	4.74 (0.35-63.44)
Unadjusted	Autumn term 2020 (open)	Men	41 - 50	Any case	1	3.36 (2.83-4.00)	1.66 (1.43-1.92)	1.32 (1.12-1.56)	2.27 (1.82-2.85)	0.94 (0.43-2.05)
Adjusted	Autumn term 2020 (open)	Men	41 - 50	Any case	1	3.22 (2.70-3.85)	1.35 (1.16-1.57)	1.10 (0.93-1.31)	2.10 (1.67-2.65)	1.11 (0.50-2.44)
Unadjusted	Autumn term 2020 (open)	Men	41 - 50	Hospitalisation	1	2.20 (1.01-4.78)	1.04 (0.49-2.20)	1.05 (0.52-2.11)	2.24 (0.84-5.99)	-
Adjusted	Autumn term 2020 (open)	Men	41 - 50	Hospitalisation	1	2.27 (1.01-5.11)	0.90 (0.41-1.97)	1.16 (0.56-2.43)	2.61 (0.93-7.32)	-
Unadjusted	Autumn term 2020 (open)	Men	51 - 65	Any case	1	3.06 (2.67-3.51)	1.87 (1.68-2.08)	1.34 (1.17-1.53)	1.46 (1.14-1.87)	0.56 (0.25-1.27)

Adjustment	Time period	Sex	Age group (years)	Outcome	Neither	Healthcare worker, patient facing	Household member of healthcare worker	Household member of teacher	Teacher	Healthcare worker, non-patient facing
Adjusted	Autumn term 2020 (open)	Men	51 - 65	Any case	1	2.94 (2.55-3.38)	1.46 (1.31-1.63)	1.06 (0.93-1.22)	1.39 (1.08-1.79)	0.76 (0.33-1.75)
Unadjusted	Autumn term 2020 (open)	Men	51 - 65	Hospitalisation	1	1.76 (1.14-2.71)	1.16 (0.81-1.66)	0.84 (0.53-1.34)	0.83 (0.33-2.06)	0.96 (0.12-7.52)
Adjusted	Autumn term 2020 (open)	Men	51 - 65	Hospitalisation	1	1.73 (1.11-2.70)	1.08 (0.74-1.58)	0.88 (0.54-1.42)	0.69 (0.26-1.82)	0.96 (0.12-7.77)
Unadjusted	Autumn term 2020 (open)	Women	21 - 30	Any case	1	3.06 (2.78-3.37)	1.35 (1.18-1.55)	1.18 (1.01-1.40)	1.56 (1.35-1.80)	1.08 (0.46-2.52)
Adjusted	Autumn term 2020 (open)	Women	21 - 30	Any case	1	3.11 (2.82-3.43)	1.27 (1.11-1.45)	1.12 (0.95-1.33)	1.60 (1.39-1.85)	1.24 (0.53-2.91)
Unadjusted	Autumn term 2020 (open)	Women	21 - 30	Hospitalisation	1	1.64 (0.71-3.77)	1.32 (0.50-3.44)	1.68 (0.55-5.14)	1.80 (0.73-4.44)	-
Adjusted	Autumn term 2020 (open)	Women	21 - 30	Hospitalisation	1	1.42 (0.57-3.54)	1.45 (0.53-3.97)	1.75 (0.51-6.02)	1.67 (0.63-4.42)	-
Unadjusted	Autumn term 2020 (open)	Women	31 - 40	Any case	1	2.60 (2.39-2.83)	1.07 (0.83-1.37)	0.80 (0.60-1.06)	1.33 (1.18-1.49)	0.26 (0.10-0.69)
Adjusted	Autumn term 2020 (open)	Women	31 - 40	Any case	1	2.64 (2.42-2.87)	0.97 (0.75-1.24)	0.75 (0.56-1.00)	1.40 (1.24-1.58)	0.29 (0.11-0.79)
Unadjusted	Autumn term 2020 (open)	Women	31 - 40	Hospitalisation	1	2.33 (1.51-3.60)	1.80 (0.61-5.37)	0.39 (0.05-2.91)	1.48 (0.83-2.64)	-
Adjusted	Autumn term 2020 (open)	Women	31 - 40	Hospitalisation	1	2.46 (1.56-3.87)	1.62 (0.52-5.06)	0.36 (0.05-2.81)	1.64 (0.89-3.00)	-
Unadjusted	Autumn term 2020 (open)	Women	41 - 50	Any case	1	2.59 (2.37-2.82)	1.33 (1.06-1.68)	0.99 (0.74-1.31)	1.35 (1.19-1.53)	0.73 (0.42-1.26)
Adjusted	Autumn term 2020 (open)	Women	41 - 50	Any case	1	2.59 (2.37-2.83)	1.15 (0.91-1.45)	0.84 (0.63-1.12)	1.31 (1.15-1.50)	0.89 (0.51-1.53)
Unadjusted	Autumn term 2020 (open)	Women	41 - 50	Hospitalisation	1	2.43 (1.58-3.73)	1.08 (0.32-3.61)	0.69 (0.16-2.98)	1.03 (0.51-2.09)	-
Adjusted	Autumn term 2020 (open)	Women	41 - 50	Hospitalisation	1	2.55 (1.63-3.99)	1.06 (0.31-3.65)	0.64 (0.14-2.88)	1.12 (0.54-2.30)	-
Unadjusted	Autumn term 2020 (open)	Women	51 - 65	Any case	1	2.46 (2.28-2.65)	1.67 (1.43-1.94)	1.12 (0.94-1.32)	1.28 (1.11-1.46)	0.69 (0.43-1.12)

Adjustment	Time period	Sex	Age group (years)	Outcome	Neither	Healthcare worker, patient facing	Household member of healthcare worker	Household member of teacher	Teacher	Healthcare worker, non-patient facing
Adjusted	Autumn term 2020 (open)	Women	51 - 65	Any case	1	2.44 (2.26-2.63)	1.37 (1.17-1.60)	0.90 (0.76-1.08)	1.26 (1.10-1.45)	0.88 (0.55-1.43)
Unadjusted	Autumn term 2020 (open)	Women	51 - 65	Hospitalisation	1	1.55 (1.14-2.09)	2.04 (1.24-3.35)	1.11 (0.59-2.08)	0.75 (0.41-1.36)	1.12 (0.26-4.87)
Adjusted	Autumn term 2020 (open)	Women	51 - 65	Hospitalisation	1	1.74 (1.27-2.40)	1.80 (1.06-3.07)	1.03 (0.53-1.99)	0.92 (0.50-1.70)	1.73 (0.37-8.04)
Unadjusted	Winter 2020/21 (closed)	Men	21 - 30	Any case	1	2.16 (1.66-2.82)	1.31 (1.17-1.48)	1.05 (0.89-1.24)	0.73 (0.48-1.11)	1.82 (0.62-5.33)
Adjusted	Winter 2020/21 (closed)	Men	21 - 30	Any case	1	2.20 (1.69-2.88)	1.19 (1.05-1.34)	0.94 (0.80-1.11)	0.72 (0.47-1.09)	2.06 (0.70-6.10)
Unadjusted	Winter 2020/21 (closed)	Men	21 - 30	Hospitalisation	1	5.12 (0.83-31.63)	0.25 (0.03-1.82)	0.52 (0.07-3.94)	-	-
Adjusted	Winter 2020/21 (closed)	Men	21 - 30	Hospitalisation	1	7.24 (0.92-56.83)	0.22 (0.03-1.63)	0.49 (0.06-4.05)	-	-
Unadjusted	Winter 2020/21 (closed)	Men	31 - 40	Any case	1	2.15 (1.73-2.67)	1.48 (1.29-1.70)	1.11 (0.94-1.30)	0.83 (0.61-1.12)	1.18 (0.56-2.46)
Adjusted	Winter 2020/21 (closed)	Men	31 - 40	Any case	1	2.17 (1.74-2.71)	1.31 (1.14-1.51)	1.06 (0.90-1.25)	0.88 (0.65-1.20)	1.58 (0.75-3.32)
Unadjusted	Winter 2020/21 (closed)	Men	31 - 40	Hospitalisation	1	3.71 (1.31-10.48)	1.96 (0.97-3.96)	0.56 (0.17-1.82)	0.77 (0.18-3.29)	3.23 (0.33-31.34)
Adjusted	Winter 2020/21 (closed)	Men	31 - 40	Hospitalisation	1	3.33 (1.09-10.20)	1.47 (0.70-3.11)	0.55 (0.17-1.83)	0.90 (0.20-3.99)	8.21 (0.73-92.27)

Adjustment	Time period	Sex	Age group (years)	Outcome	Neither	Healthcare worker, patient facing	Household member of healthcare worker	Household member of teacher	Teacher	Healthcare worker, non-patient facing
Unadjusted	Winter 2020/21 (closed)	Men	41 - 50	Any case	1	1.90 (1.53-2.35)	1.41 (1.21-1.64)	0.85 (0.70-1.03)	0.69 (0.47-1.01)	0.44 (0.16-1.20)
Adjusted	Winter 2020/21 (closed)	Men	41 - 50	Any case	1	1.86 (1.50-2.32)	1.23 (1.05-1.44)	0.76 (0.62-0.93)	0.67 (0.45-0.99)	0.52 (0.19-1.43)
Unadjusted	Winter 2020/21 (closed)	Men	41 - 50	Hospitalisation	1	1.15 (0.45-2.95)	1.13 (0.56-2.29)	0.62 (0.25-1.56)	-	-
Adjusted	Winter 2020/21 (closed)	Men	41 - 50	Hospitalisation	1	1.13 (0.43-2.95)	0.95 (0.45-1.98)	0.68 (0.26-1.76)	-	-
Unadjusted	Winter 2020/21 (closed)	Men	51 - 65	Any case	1	2.46 (2.12-2.86)	1.68 (1.51-1.87)	0.95 (0.81-1.11)	0.64 (0.45-0.91)	0.41 (0.17-1.00)
Adjusted	Winter 2020/21 (closed)	Men	51 - 65	Any case	1	2.41 (2.06-2.81)	1.32 (1.18-1.48)	0.76 (0.65-0.89)	0.62 (0.43-0.88)	0.53 (0.22-1.31)
Unadjusted	Winter 2020/21 (closed)	Men	51 - 65	Hospitalisation	1	1.08 (0.63-1.86)	1.33 (0.97-1.82)	0.75 (0.46-1.22)	0.13 (0.02-0.97)	-
Adjusted	Winter 2020/21 (closed)	Men	51 - 65	Hospitalisation	1	1.01 (0.58-1.76)	1.13 (0.81-1.57)	0.66 (0.40-1.09)	0.14 (0.02-1.05)	-
Unadjusted	Winter 2020/21 (closed)	Women	21 - 30	Any case	1	2.36 (2.13-2.62)	1.24 (1.08-1.41)	0.95 (0.79-1.14)	0.87 (0.74-1.04)	1.36 (0.68-2.75)
Adjusted	Winter 2020/21 (closed)	Women	21 - 30	Any case	1	2.41 (2.17-2.67)	1.13 (0.99-1.30)	0.88 (0.74-1.06)	0.92 (0.78-1.09)	1.71 (0.84-3.48)

Adjustment	Time period	Sex	Age group (years)	Outcome	Neither	Healthcare worker, patient facing	Household member of healthcare worker	Household member of teacher	Teacher	Healthcare worker, non-patient facing
Unadjusted	Winter 2020/21 (closed)	Women	21 - 30	Hospitalisation	1	1.79 (0.89-3.59)	1.16 (0.52-2.59)	0.68 (0.16-2.88)	0.35 (0.08-1.45)	26.61 (2.76-256.90)
Adjusted	Winter 2020/21 (closed)	Women	21 - 30	Hospitalisation	1	1.68 (0.81-3.51)	1.26 (0.55-2.90)	0.68 (0.15-2.98)	0.37 (0.09-1.60)	13.73 (1.36-138.38)
Unadjusted	Winter 2020/21 (closed)	Women	31 - 40	Any case	1	1.93 (1.75-2.11)	1.10 (0.86-1.40)	0.77 (0.58-1.04)	1.00 (0.87-1.14)	0.59 (0.30-1.15)
Adjusted	Winter 2020/21 (closed)	Women	31 - 40	Any case	1	1.97 (1.79-2.17)	0.98 (0.77-1.26)	0.70 (0.52-0.94)	1.04 (0.91-1.19)	0.71 (0.36-1.39)
Unadjusted	Winter 2020/21 (closed)	Women	31 - 40	Hospitalisation	1	1.56 (0.96-2.52)	0.67 (0.16-2.81)	1.14 (0.34-3.89)	0.93 (0.47-1.82)	-
Adjusted	Winter 2020/21 (closed)	Women	31 - 40	Hospitalisation	1	1.44 (0.87-2.38)	0.55 (0.13-2.35)	1.07 (0.31-3.75)	0.96 (0.48-1.91)	-
Unadjusted	Winter 2020/21 (closed)	Women	41 - 50	Any case	1	1.96 (1.78-2.16)	1.33 (1.04-1.72)	0.73 (0.52-1.03)	0.69 (0.58-0.83)	0.49 (0.25-0.97)
Adjusted	Winter 2020/21 (closed)	Women	41 - 50	Any case	1	2.01 (1.82-2.22)	1.14 (0.88-1.47)	0.63 (0.45-0.89)	0.71 (0.59-0.85)	0.60 (0.31-1.18)
Unadjusted	Winter 2020/21 (closed)	Women	41 - 50	Hospitalisation	1	1.07 (0.63-1.80)	1.33 (0.52-3.41)	1.31 (0.46-3.74)	0.47 (0.19-1.16)	2.96 (0.79-11.02)
Adjusted	Winter 2020/21 (closed)	Women	41 - 50	Hospitalisation	1	1.11 (0.64-1.92)	1.51 (0.57-3.98)	1.42 (0.48-4.16)	0.64 (0.25-1.62)	4.45 (1.10-18.09)

Adjustment	Time period	Sex	Age group (years)	Outcome	Neither	Healthcare worker, patient facing	Household member of healthcare worker	Household member of teacher	Teacher	Healthcare worker, non-patient facing
Unadjusted	Winter 2020/21 (closed)	Women	51 - 65	Any case	1	2.02 (1.86-2.19)	1.63 (1.39-1.91)	0.91 (0.75-1.11)	0.72 (0.61-0.86)	0.40 (0.20-0.77)
Adjusted	Winter 2020/21 (closed)	Women	51 - 65	Any case	1	2.02 (1.86-2.19)	1.30 (1.10-1.53)	0.73 (0.60-0.90)	0.72 (0.61-0.86)	0.50 (0.25-0.97)
Unadjusted	Winter 2020/21 (closed)	Women	51 - 65	Hospitalisation	1	0.77 (0.53-1.12)	1.67 (1.04-2.66)	0.44 (0.19-1.00)	0.24 (0.09-0.64)	0.69 (0.16-2.93)
Adjusted	Winter 2020/21 (closed)	Women	51 - 65	Hospitalisation	1	0.80 (0.55-1.18)	1.39 (0.86-2.26)	0.38 (0.17-0.89)	0.31 (0.11-0.86)	0.89 (0.21-3.86)
Unadjusted	Spring term 2021 (phased)	Men	21 - 30	Any case	1	0.91 (0.44-1.91)	1.01 (0.77-1.31)	0.84 (0.60-1.19)	0.87 (0.40-1.90)	-
Adjusted	Spring term 2021 (phased)	Men	21 - 30	Any case	1	0.93 (0.44-1.95)	0.95 (0.73-1.24)	0.79 (0.56-1.12)	0.95 (0.43-2.07)	-
Unadjusted	Spring term 2021 (phased)	Men	21 - 30	Hospitalisation	1	-	-	-	-	-
Adjusted	Spring term 2021 (phased)	Men	21 - 30	Hospitalisation	1	-	-	-	-	-
Unadjusted	Spring term 2021 (phased)	Men	31 - 40	Any case	1	0.27 (0.10-0.73)	0.92 (0.69-1.22)	1.07 (0.80-1.43)	1.46 (0.96-2.24)	0.52 (0.07-3.87)
Adjusted	Spring term 2021 (phased)	Men	31 - 40	Any case	1	0.24 (0.09-0.65)	0.80 (0.60-1.06)	0.97 (0.72-1.31)	1.57 (1.02-2.43)	0.74 (0.10-5.61)
Unadjusted	Spring term 2021 (phased)	Men	31 - 40	Hospitalisation	1	-	-	-	-	-
Adjusted	Spring term 2021 (phased)	Men	31 - 40	Hospitalisation	1	-	-	-	-	-
Unadjusted	Spring term 2021 (phased)	Men	41 - 50	Any case	1	0.61 (0.33-1.13)	0.98 (0.71-1.34)	1.67 (1.26-2.21)	1.48 (0.90-2.45)	-
Adjusted	Spring term 2021 (phased)	Men	41 - 50	Any case	1	0.56 (0.30-1.04)	0.80 (0.58-1.11)	1.39 (1.04-1.86)	1.39 (0.83-2.34)	-

Adjustment	Time period	Sex	Age group (years)	Outcome	Neither	Healthcare worker, patient facing	Household member of healthcare worker	Household member of teacher	Teacher	Healthcare worker, non-patient facing
Unadjusted	Spring term 2021 (phased)	Men	41 - 50	Hospitalisation	1	-	-	-	-	-
Adjusted	Spring term 2021 (phased)	Men	41 - 50	Hospitalisation	1	-	-	-	-	-
Unadjusted	Spring term 2021 (phased)	Men	51 - 65	Any case	1	0.61 (0.35-1.07)	0.99 (0.73-1.33)	0.83 (0.58-1.19)	1.24 (0.70-2.17)	0.30 (0.04-2.19)
Adjusted	Spring term 2021 (phased)	Men	51 - 65	Any case	1	0.61 (0.35-1.09)	0.79 (0.58-1.08)	0.68 (0.47-0.98)	1.23 (0.69-2.18)	0.38 (0.05-2.81)
Unadjusted	Spring term 2021 (phased)	Men	51 - 65	Hospitalisation	1	-	0.23 (0.06-0.93)	0.18 (0.02-1.28)	1.27 (0.29-5.63)	-
Adjusted	Spring term 2021 (phased)	Men	51 - 65	Hospitalisation	1	-	0.24 (0.06-1.01)	0.17 (0.02-1.29)	1.75 (0.38-7.94)	-
Unadjusted	Spring term 2021 (phased)	Women	21 - 30	Any case	1	0.65 (0.47-0.91)	0.55 (0.38-0.79)	0.64 (0.42-0.97)	1.65 (1.27-2.15)	-
Adjusted	Spring term 2021 (phased)	Women	21 - 30	Any case	1	0.70 (0.50-0.98)	0.49 (0.34-0.71)	0.61 (0.40-0.93)	1.85 (1.41-2.42)	-
Unadjusted	Spring term 2021 (phased)	Women	21 - 30	Hospitalisation	1	-	-	-	-	-
Adjusted	Spring term 2021 (phased)	Women	21 - 30	Hospitalisation	1	-	-	-	-	-
Unadjusted	Spring term 2021 (phased)	Women	31 - 40	Any case	1	0.53 (0.40-0.72)	0.36 (0.18-0.73)	1.03 (0.62-1.70)	1.46 (1.19-1.79)	0.85 (0.30-2.37)
Adjusted	Spring term 2021 (phased)	Women	31 - 40	Any case	1	0.52 (0.38-0.71)	0.31 (0.15-0.63)	0.86 (0.52-1.44)	1.60 (1.29-1.99)	1.08 (0.38-3.10)
Unadjusted	Spring term 2021 (phased)	Women	31 - 40	Hospitalisation	1	0.17 (0.02-1.28)	-	-	1.02 (0.40-2.63)	2.92 (0.30-28.14)
Adjusted	Spring term 2021 (phased)	Women	31 - 40	Hospitalisation	1	0.16 (0.02-1.21)	-	-	1.18 (0.43-3.29)	3.04 (0.29-31.61)
Unadjusted	Spring term 2021 (phased)	Women	41 - 50	Any case	1	0.59 (0.44-0.80)	0.98 (0.57-1.68)	0.97 (0.53-1.76)	1.58 (1.24-2.00)	0.77 (0.28-2.14)
Adjusted	Spring term 2021 (phased)	Women	41 - 50	Any case	1	0.56 (0.41-0.76)	0.88 (0.51-1.52)	0.78 (0.42-1.42)	1.66 (1.30-2.12)	0.96 (0.34-2.73)

Adjustment	Time period	Sex	Age group (years)	Outcome	Neither	Healthcare worker, patient facing	Household member of healthcare worker	Household member of teacher	Teacher	Healthcare worker, non-patient facing
Unadjusted	Spring term 2021 (phased)	Women	41 - 50	Hospitalisation	1	0.53 (0.16-1.74)	1.71 (0.38-7.79)	1.88 (0.22-16.40)	0.31 (0.04-2.29)	1.16 (0.14-9.85)
Adjusted	Spring term 2021 (phased)	Women	41 - 50	Hospitalisation	1	0.54 (0.16-1.80)	1.79 (0.38-8.38)	1.94 (0.22-16.83)	0.29 (0.04-2.20)	1.42 (0.16-12.78)
Unadjusted	Spring term 2021 (phased)	Women	51 - 65	Any case	1	0.66 (0.50-0.88)	0.77 (0.47-1.25)	1.10 (0.72-1.69)	1.42 (1.04-1.94)	0.72 (0.26-1.99)
Adjusted	Spring term 2021 (phased)	Women	51 - 65	Any case	1	0.65 (0.49-0.87)	0.61 (0.37-1.00)	0.83 (0.53-1.28)	1.47 (1.07-2.03)	0.94 (0.34-2.64)
Unadjusted	Spring term 2021 (phased)	Women	51 - 65	Hospitalisation	1	0.38 (0.12-1.24)	0.39 (0.05-2.88)	1.23 (0.37-4.13)	0.71 (0.22-2.31)	0.85 (0.11-6.60)
Adjusted	Spring term 2021 (phased)	Women	51 - 65	Hospitalisation	1	0.43 (0.13-1.41)	0.32 (0.04-2.59)	1.28 (0.37-4.47)	0.75 (0.22-2.54)	0.97 (0.12-8.00)
Unadjusted	Summer term 2021 (open)	Men	21 - 30	Any case	1	0.45 (0.29-0.71)	1.18 (1.07-1.32)	1.32 (1.18-1.49)	1.68 (1.29-2.20)	0.47 (0.11-1.96)
Adjusted	Summer term 2021 (open)	Men	21 - 30	Any case	1	0.44 (0.28-0.69)	1.07 (0.96-1.19)	1.15 (1.02-1.29)	1.55 (1.18-2.03)	0.45 (0.11-1.89)
Unadjusted	Summer term 2021 (open)	Men	21 - 30	Hospitalisation	1	-	-	-	-	-
Adjusted	Summer term 2021 (open)	Men	21 - 30	Hospitalisation	1	-	-	-	-	-
Unadjusted	Summer term 2021 (open)	Men	31 - 40	Any case	1	0.36 (0.22-0.59)	1.15 (0.98-1.36)	1.33 (1.12-1.57)	1.85 (1.46-2.36)	0.56 (0.17-1.79)
Adjusted	Summer term 2021 (open)	Men	31 - 40	Any case	1	0.36 (0.22-0.59)	1.05 (0.89-1.24)	1.21 (1.02-1.44)	1.73 (1.35-2.21)	0.65 (0.20-2.10)
Unadjusted	Summer term 2021 (open)	Men	31 - 40	Hospitalisation	1	-	1.25 (0.48-3.23)	0.22 (0.03-1.58)	1.68 (0.36-7.82)	-
Adjusted	Summer term 2021 (open)	Men	31 - 40	Hospitalisation	1	-	1.09 (0.41-2.89)	0.21 (0.03-1.55)	2.02 (0.41-9.84)	-
Unadjusted	Summer term 2021 (open)	Men	41 - 50	Any case	1	0.43 (0.27-0.68)	1.18 (0.96-1.45)	1.46 (1.21-1.77)	2.07 (1.56-2.74)	0.29 (0.07-1.17)
Adjusted	Summer term 2021 (open)	Men	41 - 50	Any case	1	0.38 (0.24-0.62)	0.97 (0.79-1.19)	1.14 (0.93-1.39)	1.83 (1.37-2.46)	0.41 (0.10-1.70)

Adjustment	Time period	Sex	Age group (years)	Outcome	Neither	Healthcare worker, patient facing	Household member of healthcare worker	Household member of teacher	Teacher	Healthcare worker, non-patient facing
Unadjusted	Summer term 2021 (open)	Men	41 - 50	Hospitalisation	1	-	0.23 (0.03-1.68)	-	2.60 (0.71-9.56)	-
Adjusted	Summer term 2021 (open)	Men	41 - 50	Hospitalisation	1	-	0.22 (0.03-1.71)	-	3.16 (0.81-12.35)	-
Unadjusted	Summer term 2021 (open)	Men	51 - 65	Any case	1	0.54 (0.35-0.82)	1.14 (0.93-1.39)	1.42 (1.14-1.78)	1.88 (1.31-2.68)	0.62 (0.19-2.01)
Adjusted	Summer term 2021 (open)	Men	51 - 65	Any case	1	0.53 (0.35-0.82)	0.92 (0.75-1.13)	1.07 (0.86-1.35)	1.64 (1.14-2.36)	0.91 (0.28-2.98)
Unadjusted	Summer term 2021 (open)	Men	51 - 65	Hospitalisation	1	0.50 (0.07-3.74)	0.75 (0.27-2.11)	-	-	-
Adjusted	Summer term 2021 (open)	Men	51 - 65	Hospitalisation	1	0.53 (0.07-4.12)	0.78 (0.26-2.27)	-	-	-
Unadjusted	Summer term 2021 (open)	Women	21 - 30	Any case	1	0.53 (0.43-0.65)	1.15 (0.99-1.32)	1.22 (1.04-1.44)	1.92 (1.67-2.20)	1.03 (0.41-2.60)
Adjusted	Summer term 2021 (open)	Women	21 - 30	Any case	1	0.53 (0.43-0.65)	1.05 (0.91-1.21)	1.11 (0.94-1.31)	1.88 (1.64-2.16)	1.20 (0.47-3.03)
Unadjusted	Summer term 2021 (open)	Women	21 - 30	Hospitalisation	1	0.83 (0.29-2.34)	1.18 (0.45-3.09)	0.41 (0.05-3.04)	0.82 (0.25-2.76)	-
Adjusted	Summer term 2021 (open)	Women	21 - 30	Hospitalisation	1	1.01 (0.35-2.92)	0.99 (0.37-2.64)	0.60 (0.08-4.57)	1.01 (0.29-3.48)	-
Unadjusted	Summer term 2021 (open)	Women	31 - 40	Any case	1	0.67 (0.56-0.80)	0.69 (0.48-0.98)	0.87 (0.61-1.24)	1.66 (1.45-1.90)	0.47 (0.21-1.07)
Adjusted	Summer term 2021 (open)	Women	31 - 40	Any case	1	0.66 (0.55-0.80)	0.56 (0.39-0.80)	0.73 (0.51-1.06)	1.80 (1.56-2.07)	0.61 (0.27-1.40)
Unadjusted	Summer term 2021 (open)	Women	31 - 40	Hospitalisation	1	0.82 (0.41-1.66)	0.64 (0.08-4.87)	-	0.81 (0.37-1.79)	-
Adjusted	Summer term 2021 (open)	Women	31 - 40	Hospitalisation	1	0.74 (0.36-1.51)	0.66 (0.08-5.23)	-	0.84 (0.37-1.90)	-
Unadjusted	Summer term 2021 (open)	Women	41 - 50	Any case	1	0.66 (0.54-0.81)	0.52 (0.32-0.84)	0.98 (0.64-1.49)	1.64 (1.40-1.92)	0.62 (0.29-1.33)
Adjusted	Summer term 2021 (open)	Women	41 - 50	Any case	1	0.63 (0.51-0.78)	0.43 (0.27-0.70)	0.80 (0.52-1.24)	1.55 (1.32-1.83)	0.86 (0.39-1.89)

Adjustment	Time period	Sex	Age group (years)	Outcome	Neither	Healthcare worker, patient facing	Household member of healthcare worker	Household member of teacher	Teacher	Healthcare worker, non-patient facing
Unadjusted	Summer term 2021 (open)	Women	41 - 50	Hospitalisation	1	0.34 (0.11-1.12)	-	1.16 (0.14-9.34)	0.54 (0.17-1.79)	-
Adjusted	Summer term 2021 (open)	Women	41 - 50	Hospitalisation	1	0.32 (0.10-1.06)	-	1.35 (0.16-11.61)	0.74 (0.22-2.50)	-
Unadjusted	Summer term 2021 (open)	Women	51 - 65	Any case	1	0.77 (0.63-0.95)	0.53 (0.35-0.81)	1.48 (1.10-2.00)	1.77 (1.44-2.18)	0.57 (0.25-1.30)
Adjusted	Summer term 2021 (open)	Women	51 - 65	Any case	1	0.72 (0.58-0.90)	0.38 (0.25-0.58)	1.06 (0.78-1.43)	1.60 (1.30-1.99)	0.87 (0.38-2.02)
Unadjusted	Summer term 2021 (open)	Women	51 - 65	Hospitalisation	1	0.54 (0.22-1.36)	-	0.36 (0.05-2.67)	0.35 (0.08-1.48)	-
Adjusted	Summer term 2021 (open)	Women	51 - 65	Hospitalisation	1	0.58 (0.23-1.47)	-	0.35 (0.05-2.73)	0.38 (0.09-1.62)	-

Table S3b Rate ratios for any case, hospitalisation with COVID-19 and severe COVID-19 for teachers by sector, stratified by time period, age and sex

Adjustment	Time period	Sex	Age group (years)	Outcome	Household member of primary teacher	Neither	Household member of secondary teacher	Household member other teacher	Nursery/Primary or Nursery	Primary	Secondary	Teacher in other sector
Unadjusted	Spring/Summer 2020 (closed)	Men	21 - 30	Any case	0.21 (0.05-0.86)	1	1.12 (0.46-2.71)	0.62 (0.12-3.25)	-	2.44 (0.24-24.42)	-	7.31 (0.97-55.34)
Adjusted	Spring/Summer 2020 (closed)	Men	21 - 30	Any case	0.19 (0.05-0.78)	1	0.98 (0.40-2.39)	0.57 (0.11-3.13)	-	2.32 (0.22-24.18)	-	7.70 (1.00-59.44)
Unadjusted	Spring/Summer 2020 (closed)	Men	21 - 30	Hospitalisation	-	1	-	-	-	-	-	-
Adjusted	Spring/Summer 2020 (closed)	Men	21 - 30	Hospitalisation	-	1	-	-	-	-	-	-
Unadjusted	Spring/Summer 2020 (closed)	Men	31 - 40	Any case	0.82 (0.40-1.66)	1	0.53 (0.19-1.46)	1.10 (0.25-4.78)	1.44 (0.17-11.90)	1.01 (0.13-7.95)	0.29 (0.04-2.15)	-
Adjusted	Spring/Summer 2020 (closed)	Men	31 - 40	Any case	0.78 (0.38-1.60)	1	0.39 (0.13-1.20)	1.07 (0.24-4.71)	1.56 (0.19-13.02)	1.17 (0.15-9.19)	0.30 (0.04-2.22)	-
Unadjusted	Spring/Summer 2020 (closed)	Men	31 - 40	Hospitalisation	0.49 (0.06-3.65)	1	-	-	-	-	-	-
Adjusted	Spring/Summer 2020 (closed)	Men	31 - 40	Hospitalisation	0.42 (0.05-3.53)	1	-	-	-	-	-	-
Unadjusted	Spring/Summer 2020 (closed)	Men	41 - 50	Any case	0.75 (0.34-1.66)	1	0.64 (0.24-1.72)	1.10 (0.29-4.13)	-	2.26 (0.26-19.43)	0.56 (0.13-2.38)	3.54 (1.08-11.62)
Adjusted	Spring/Summer 2020 (closed)	Men	41 - 50	Any case	0.70 (0.32-1.56)	1	0.56 (0.20-1.55)	0.94 (0.25-3.50)	-	2.34 (0.27-20.36)	0.53 (0.12-2.28)	3.34 (1.02-10.93)

Adjustment	Time period	Sex	Age group (years)	Outcome	Household member of primary teacher	Neither	Household member of secondary teacher	Household member other teacher	Nursery/Primary or Nursery	Primary	Secondary	Teacher in other sector
Unadjusted	Spring/Summer 2020 (closed)	Men	41 - 50	Hospitalisation	0.86 (0.24-3.02)	1	0.71 (0.16-3.26)	-	-	-	0.65 (0.08-5.11)	-
Adjusted	Spring/Summer 2020 (closed)	Men	41 - 50	Hospitalisation	0.79 (0.21-2.92)	1	0.72 (0.15-3.43)	-	-	-	0.67 (0.08-5.67)	-
Unadjusted	Spring/Summer 2020 (closed)	Men	51 - 65	Any case	0.77 (0.44-1.34)	1	1.40 (0.87-2.27)	1.04 (0.47-2.28)	-	2.52 (0.29-21.81)	0.62 (0.24-1.55)	0.46 (0.06-3.44)
Adjusted	Spring/Summer 2020 (closed)	Men	51 - 65	Any case	0.73 (0.42-1.28)	1	1.27 (0.78-2.06)	1.05 (0.47-2.31)	-	2.95 (0.34-25.66)	0.65 (0.26-1.64)	0.51 (0.07-3.78)
Unadjusted	Spring/Summer 2020 (closed)	Men	51 - 65	Hospitalisation	0.50 (0.18-1.38)	1	1.02 (0.47-2.25)	-	-	-	0.64 (0.15-2.73)	0.85 (0.11-6.56)
Adjusted	Spring/Summer 2020 (closed)	Men	51 - 65	Hospitalisation	0.54 (0.19-1.52)	1	0.91 (0.40-2.05)	-	-	-	0.71 (0.17-3.03)	0.92 (0.12-7.37)
Unadjusted	Spring/Summer 2020 (closed)	Women	21 - 30	Any case	0.53 (0.22-1.30)	1	0.74 (0.35-1.53)	-	0.39 (0.12-1.25)	0.58 (0.21-1.60)	0.12 (0.02-0.84)	0.32 (0.04-2.37)
Adjusted	Spring/Summer 2020 (closed)	Women	21 - 30	Any case	0.55 (0.22-1.34)	1	0.76 (0.36-1.59)	-	0.42 (0.13-1.34)	0.64 (0.23-1.77)	0.13 (0.02-0.91)	0.35 (0.05-2.56)
Unadjusted	Spring/Summer 2020 (closed)	Women	21 - 30	Hospitalisation	-	1	-	-	-	1.82 (0.21-15.43)	-	-
Adjusted	Spring/Summer 2020 (closed)	Women	21 - 30	Hospitalisation	-	1	-	-	-	4.19 (0.39-44.90)	-	-

Adjustment	Time period	Sex	Age group (years)	Outcome	Household member of primary teacher	Neither	Household member of secondary teacher	Household member other teacher	Nursery/Primary or Nursery	Primary	Secondary	Teacher in other sector
Unadjusted	Spring/Summer 2020 (closed)	Women	31 - 40	Any case	0.46 (0.10-2.14)	1	0.77 (0.32-1.84)	1.63 (0.46-5.80)	0.47 (0.19-1.16)	0.66 (0.29-1.52)	0.48 (0.21-1.08)	0.57 (0.18-1.82)
Adjusted	Spring/Summer 2020 (closed)	Women	31 - 40	Any case	0.45 (0.09-2.18)	1	0.82 (0.34-1.96)	1.72 (0.49-6.08)	0.53 (0.21-1.29)	0.70 (0.31-1.61)	0.43 (0.18-1.07)	0.61 (0.19-1.97)
Unadjusted	Spring/Summer 2020 (closed)	Women	31 - 40	Hospitalisation	-	1	-	-	0.94 (0.12-7.36)	0.53 (0.07-4.04)	1.62 (0.36-7.36)	0.89 (0.11-7.12)
Adjusted	Spring/Summer 2020 (closed)	Women	31 - 40	Hospitalisation	-	1	-	-	1.10 (0.14-8.68)	0.53 (0.07-4.27)	2.17 (0.45-10.40)	1.19 (0.15-9.68)
Unadjusted	Spring/Summer 2020 (closed)	Women	41 - 50	Any case	0.38 (0.11-1.28)	1	0.77 (0.31-1.91)	1.35 (0.32-5.67)	0.51 (0.21-1.25)	0.23 (0.06-0.93)	0.07 (0.01-0.52)	0.83 (0.33-2.07)
Adjusted	Spring/Summer 2020 (closed)	Women	41 - 50	Any case	0.35 (0.10-1.18)	1	0.83 (0.33-2.06)	1.49 (0.37-6.04)	0.57 (0.23-1.41)	0.25 (0.06-1.03)	0.08 (0.01-0.55)	0.91 (0.36-2.28)
Unadjusted	Spring/Summer 2020 (closed)	Women	41 - 50	Hospitalisation	-	1	0.97 (0.11-8.51)	-	0.46 (0.06-3.44)	0.84 (0.11-6.45)	0.41 (0.06-3.06)	0.73 (0.09-5.63)
Adjusted	Spring/Summer 2020 (closed)	Women	41 - 50	Hospitalisation	-	1	1.26 (0.14-11.17)	-	0.36 (0.05-2.83)	1.07 (0.14-8.39)	0.53 (0.07-3.97)	0.71 (0.09-5.73)
Unadjusted	Spring/Summer 2020 (closed)	Women	51 - 65	Any case	0.96 (0.56-1.67)	1	0.71 (0.40-1.25)	0.68 (0.20-2.35)	0.09 (0.01-0.62)	0.08 (0.01-0.58)	0.25 (0.09-0.67)	0.46 (0.17-1.26)
Adjusted	Spring/Summer 2020 (closed)	Women	51 - 65	Any case	0.99 (0.57-1.71)	1	0.73 (0.41-1.29)	0.72 (0.21-2.44)	0.10 (0.01-0.69)	0.09 (0.01-0.65)	0.28 (0.10-0.76)	0.49 (0.18-1.34)

Adjustment	Time period	Sex	Age group (years)	Outcome	Household member of primary teacher	Neither	Household member of secondary teacher	Household member other teacher	Nursery/Primary or Nursery	Primary	Secondary	Teacher in other sector
Unadjusted	Spring/Summer 2020 (closed)	Women	51 - 65	Hospitalisation	1.69 (0.70-4.10)	1	0.35 (0.08-1.45)	1.00 (0.13-8.01)	-	-	0.20 (0.03-1.45)	0.48 (0.06-3.57)
Adjusted	Spring/Summer 2020 (closed)	Women	51 - 65	Hospitalisation	1.83 (0.72-4.65)	1	0.39 (0.09-1.67)	0.91 (0.11-7.51)	-	-	0.27 (0.04-1.98)	0.60 (0.08-4.55)
Unadjusted	Autumn term 2020 (open)	Men	21 - 30	Any case	1.30 (1.04-1.62)	1	1.27 (0.97-1.67)	1.33 (0.89-1.98)	1.93 (0.74-5.05)	1.52 (0.68-3.39)	2.29 (1.57-3.34)	3.45 (1.23-9.69)
Adjusted	Autumn term 2020 (open)	Men	21 - 30	Any case	1.17 (0.94-1.46)	1	1.12 (0.85-1.46)	1.18 (0.79-1.76)	1.78 (0.68-4.68)	1.52 (0.68-3.40)	2.27 (1.55-3.33)	3.12 (1.09-8.91)
Unadjusted	Autumn term 2020 (open)	Men	21 - 30	Hospitalisation	-	1	-	-	-	-	-	-
Adjusted	Autumn term 2020 (open)	Men	21 - 30	Hospitalisation	-	1	-	-	-	-	-	-
Unadjusted	Autumn term 2020 (open)	Men	31 - 40	Any case	1.14 (0.91-1.43)	1	1.20 (0.91-1.57)	1.23 (0.79-1.92)	2.54 (1.48-4.37)	2.13 (1.22-3.74)	2.30 (1.73-3.06)	2.45 (1.30-4.64)
Adjusted	Autumn term 2020 (open)	Men	31 - 40	Any case	1.03 (0.82-1.29)	1	1.12 (0.85-1.47)	1.15 (0.74-1.79)	2.58 (1.49-4.47)	2.06 (1.17-3.62)	2.25 (1.68-3.02)	2.46 (1.29-4.69)
Unadjusted	Autumn term 2020 (open)	Men	31 - 40	Hospitalisation	1.18 (0.35-4.05)	1	-	-	4.07 (0.36-45.50)	-	0.91 (0.12-7.09)	-
Adjusted	Autumn term 2020 (open)	Men	31 - 40	Hospitalisation	1.32 (0.36-4.88)	1	-	-	6.90 (0.44-108.17)	-	0.89 (0.10-7.79)	-
Unadjusted	Autumn term 2020 (open)	Men	41 - 50	Any case	1.39 (1.12-1.74)	1	1.15 (0.86-1.54)	1.48 (0.99-2.23)	1.34 (0.57-3.17)	2.50 (1.42-4.43)	2.15 (1.59-2.92)	2.97 (1.88-4.69)

Adjustment	Time period	Sex	Age group (years)	Outcome	Household member of primary teacher	Neither	Household member of secondary teacher	Household member other teacher	Nursery/Primary or Nursery	Primary	Secondary	Teacher in other sector
Adjusted	Autumn term 2020 (open)	Men	41 - 50	Any case	1.14 (0.91-1.43)	1	0.95 (0.71-1.28)	1.35 (0.89-2.04)	1.48 (0.62-3.53)	2.12 (1.18-3.79)	2.01 (1.47-2.74)	2.66 (1.66-4.24)
Unadjusted	Autumn term 2020 (open)	Men	41 - 50	Hospitalisation	1.46 (0.56-3.81)	1	0.29 (0.04-2.16)	1.65 (0.48-5.69)	-	-	2.19 (0.61-7.83)	10.02 (1.41-71.25)
Adjusted	Autumn term 2020 (open)	Men	41 - 50	Hospitalisation	1.63 (0.59-4.49)	1	0.31 (0.04-2.35)	2.08 (0.58-7.41)	-	-	3.31 (0.86-12.73)	6.52 (0.89-47.75)
Unadjusted	Autumn term 2020 (open)	Men	51 - 65	Any case	1.36 (1.13-1.63)	1	1.31 (1.03-1.66)	1.34 (0.93-1.93)	3.23 (1.56-6.67)	0.28 (0.04-2.07)	1.46 (1.08-1.98)	1.31 (0.74-2.30)
Adjusted	Autumn term 2020 (open)	Men	51 - 65	Any case	1.08 (0.90-1.30)	1	1.02 (0.80-1.30)	1.12 (0.77-1.62)	2.77 (1.32-5.79)	0.28 (0.04-2.05)	1.41 (1.04-1.92)	1.24 (0.70-2.21)
Unadjusted	Autumn term 2020 (open)	Men	51 - 65	Hospitalisation	0.74 (0.37-1.47)	1	0.97 (0.44-2.11)	0.94 (0.33-2.64)	1.30 (0.16-10.58)	-	0.73 (0.22-2.36)	1.08 (0.13-8.64)
Adjusted	Autumn term 2020 (open)	Men	51 - 65	Hospitalisation	0.81 (0.40-1.63)	1	0.95 (0.43-2.11)	0.93 (0.32-2.70)	0.64 (0.06-6.93)	-	0.73 (0.22-2.46)	0.66 (0.07-5.78)
Unadjusted	Autumn term 2020 (open)	Women	21 - 30	Any case	1.19 (0.94-1.50)	1	1.13 (0.86-1.48)	1.32 (0.89-1.95)	1.29 (0.96-1.74)	1.88 (1.49-2.36)	1.40 (1.09-1.81)	1.78 (1.08-2.96)
Adjusted	Autumn term 2020 (open)	Women	21 - 30	Any case	1.11 (0.88-1.41)	1	1.07 (0.81-1.40)	1.30 (0.88-1.92)	1.33 (0.98-1.80)	1.92 (1.52-2.41)	1.45 (1.13-1.88)	1.87 (1.13-3.11)
Unadjusted	Autumn term 2020 (open)	Women	21 - 30	Hospitalisation	2.34 (0.47-11.64)	1	-	3.84 (0.67-22.13)	1.31 (0.16-10.52)	3.12 (0.81-12.00)	1.77 (0.38-8.18)	-

Adjustment	Time period	Sex	Age group (years)	Outcome	Household member of primary teacher	Neither	Household member of secondary teacher	Household member other teacher	Nursery/Primary or Nursery	Primary	Secondary	Teacher in other sector
Adjusted	Autumn term 2020 (open)	Women	21 - 30	Hospitalisation	2.82 (0.49-16.20)	1	-	3.91 (0.59-25.93)	1.55 (0.18-13.42)	3.25 (0.79-13.31)	1.28 (0.22-7.38)	-
Unadjusted	Autumn term 2020 (open)	Women	31 - 40	Any case	0.71 (0.43-1.18)	1	0.79 (0.53-1.19)	1.06 (0.53-2.12)	1.11 (0.87-1.41)	1.46 (1.19-1.78)	1.39 (1.14-1.69)	1.34 (0.95-1.87)
Adjusted	Autumn term 2020 (open)	Women	31 - 40	Any case	0.66 (0.40-1.09)	1	0.74 (0.49-1.11)	1.03 (0.51-2.07)	1.18 (0.93-1.50)	1.52 (1.24-1.87)	1.48 (1.21-1.80)	1.37 (0.98-1.93)
Unadjusted	Autumn term 2020 (open)	Women	31 - 40	Hospitalisation	1.25 (0.15-10.37)	1	-	-	0.45 (0.06-3.37)	1.08 (0.33-3.58)	1.93 (0.84-4.41)	3.52 (0.93-13.30)
Adjusted	Autumn term 2020 (open)	Women	31 - 40	Hospitalisation	0.88 (0.09-8.31)	1	-	-	0.54 (0.07-4.11)	1.12 (0.33-3.80)	2.17 (0.92-5.10)	3.73 (0.96-14.52)
Unadjusted	Autumn term 2020 (open)	Women	41 - 50	Any case	1.04 (0.64-1.67)	1	1.04 (0.70-1.53)	0.75 (0.35-1.65)	1.32 (1.02-1.71)	1.54 (1.22-1.95)	1.25 (1.00-1.55)	1.30 (0.95-1.79)
Adjusted	Autumn term 2020 (open)	Women	41 - 50	Any case	0.88 (0.54-1.42)	1	0.87 (0.58-1.29)	0.69 (0.32-1.51)	1.28 (0.98-1.66)	1.47 (1.17-1.86)	1.23 (0.99-1.54)	1.28 (0.93-1.77)
Unadjusted	Autumn term 2020 (open)	Women	41 - 50	Hospitalisation	-	1	1.18 (0.26-5.29)	-	-	0.95 (0.22-4.09)	1.23 (0.43-3.52)	5.14 (1.28-20.66)
Adjusted	Autumn term 2020 (open)	Women	41 - 50	Hospitalisation	-	1	0.98 (0.21-4.61)	-	-	0.84 (0.19-3.71)	1.41 (0.48-4.17)	5.38 (1.27-22.84)
Unadjusted	Autumn term 2020 (open)	Women	51 - 65	Any case	1.06 (0.82-1.37)	1	1.09 (0.84-1.41)	1.44 (0.91-2.26)	1.29 (0.98-1.70)	1.80 (1.42-2.28)	0.98 (0.75-1.28)	1.10 (0.79-1.53)

Adjustment	Time period	Sex	Age group (years)	Outcome	Household member of primary teacher	Neither	Household member of secondary teacher	Household member other teacher	Nursery/Primary or Nursery	Primary	Secondary	Teacher in other sector
Adjusted	Autumn term 2020 (open)	Women	51 - 65	Any case	0.82 (0.63-1.07)	1	0.91 (0.70-1.18)	1.25 (0.79-1.98)	1.24 (0.94-1.64)	1.74 (1.37-2.22)	1.00 (0.76-1.31)	1.09 (0.78-1.53)
Unadjusted	Autumn term 2020 (open)	Women	51 - 65	Hospitalisation	1.31 (0.56-3.11)	1	0.72 (0.22-2.35)	1.65 (0.36-7.48)	0.62 (0.15-2.60)	0.50 (0.12-2.07)	0.97 (0.38-2.44)	0.83 (0.26-2.72)
Adjusted	Autumn term 2020 (open)	Women	51 - 65	Hospitalisation	1.11 (0.46-2.71)	1	0.68 (0.20-2.32)	2.10 (0.44-10.02)	0.68 (0.16-2.93)	0.68 (0.16-2.93)	1.05 (0.40-2.73)	1.22 (0.36-4.12)
Unadjusted	Winter 2020/21 (closed)	Men	21 - 30	Any case	0.98 (0.78-1.23)	1	1.13 (0.85-1.49)	1.16 (0.77-1.73)	0.85 (0.30-2.39)	0.53 (0.17-1.71)	0.77 (0.46-1.29)	0.54 (0.07-4.11)
Adjusted	Winter 2020/21 (closed)	Men	21 - 30	Any case	0.87 (0.69-1.10)	1	1.01 (0.76-1.34)	1.06 (0.70-1.60)	0.91 (0.32-2.56)	0.48 (0.15-1.56)	0.76 (0.46-1.28)	0.53 (0.07-4.05)
Unadjusted	Winter 2020/21 (closed)	Men	21 - 30	Hospitalisation	-	1	1.98 (0.23-17.10)	-	-	-	-	-
Adjusted	Winter 2020/21 (closed)	Men	21 - 30	Hospitalisation	-	1	1.53 (0.16-14.19)	-	-	-	-	-
Unadjusted	Winter 2020/21 (closed)	Men	31 - 40	Any case	1.32 (1.08-1.61)	1	0.87 (0.64-1.18)	0.82 (0.50-1.35)	0.98 (0.45-2.15)	1.08 (0.49-2.36)	0.78 (0.52-1.16)	0.68 (0.27-1.70)
Adjusted	Winter 2020/21 (closed)	Men	31 - 40	Any case	1.26 (1.03-1.54)	1	0.81 (0.59-1.11)	0.82 (0.50-1.36)	1.06 (0.48-2.35)	1.17 (0.53-2.59)	0.83 (0.56-1.25)	0.70 (0.28-1.75)
Unadjusted	Winter 2020/21 (closed)	Men	31 - 40	Hospitalisation	0.36 (0.05-2.64)	1	1.10 (0.25-4.76)	-	3.40 (0.35-32.80)	-	-	3.67 (0.33-40.77)

Adjustment	Time period	Sex	Age group (years)	Outcome	Household member of primary teacher	Neither	Household member of secondary teacher	Household member other teacher	Nursery/Primary or Nursery	Primary	Secondary	Teacher in other sector
Adjusted	Winter 2020/21 (closed)	Men	31 - 40	Hospitalisation	0.35 (0.05-2.62)	1	0.94 (0.21-4.30)	-	4.22 (0.39-45.09)	-	-	2.62 (0.21-32.51)
Unadjusted	Winter 2020/21 (closed)	Men	41 - 50	Any case	0.77 (0.59-1.01)	1	1.13 (0.84-1.53)	0.53 (0.28-1.00)	0.76 (0.24-2.48)	2.01 (0.94-4.34)	0.43 (0.24-0.79)	0.81 (0.35-1.87)
Adjusted	Winter 2020/21 (closed)	Men	41 - 50	Any case	0.69 (0.52-0.91)	1	1.01 (0.74-1.37)	0.48 (0.25-0.90)	0.89 (0.27-2.90)	1.98 (0.91-4.32)	0.42 (0.23-0.77)	0.75 (0.32-1.74)
Unadjusted	Winter 2020/21 (closed)	Men	41 - 50	Hospitalisation	0.76 (0.23-2.50)	1	0.37 (0.05-2.77)	0.72 (0.09-5.49)	-	-	-	-
Adjusted	Winter 2020/21 (closed)	Men	41 - 50	Hospitalisation	0.64 (0.19-2.21)	1	0.50 (0.06-3.79)	1.34 (0.17-10.59)	-	-	-	-
Unadjusted	Winter 2020/21 (closed)	Men	51 - 65	Any case	0.98 (0.80-1.21)	1	0.86 (0.65-1.14)	1.02 (0.68-1.53)	1.12 (0.34-3.73)	0.91 (0.28-2.97)	0.49 (0.30-0.80)	0.91 (0.48-1.74)
Adjusted	Winter 2020/21 (closed)	Men	51 - 65	Any case	0.76 (0.61-0.93)	1	0.70 (0.53-0.92)	0.94 (0.62-1.41)	0.97 (0.29-3.25)	0.88 (0.26-2.92)	0.48 (0.29-0.78)	0.87 (0.45-1.67)
Unadjusted	Winter 2020/21 (closed)	Men	51 - 65	Hospitalisation	0.92 (0.50-1.67)	1	0.38 (0.12-1.19)	1.04 (0.31-3.43)	-	-	-	0.46 (0.06-3.44)
Adjusted	Winter 2020/21 (closed)	Men	51 - 65	Hospitalisation	0.88 (0.47-1.62)	1	0.28 (0.09-0.92)	0.94 (0.28-3.20)	-	-	-	0.54 (0.07-4.09)
Unadjusted	Winter 2020/21 (closed)	Women	21 - 30	Any case	1.10 (0.86-1.42)	1	0.75 (0.54-1.04)	0.98 (0.63-1.53)	0.86 (0.63-1.18)	0.95 (0.71-1.27)	0.85 (0.63-1.14)	0.75 (0.40-1.39)

Adjustment	Time period	Sex	Age group (years)	Outcome	Household member of primary teacher	Neither	Household member of secondary teacher	Household member other teacher	Nursery/Primary or Nursery	Primary	Secondary	Teacher in other sector
Adjusted	Winter 2020/21 (closed)	Women	21 - 30	Any case	1.02 (0.79-1.31)	1	0.70 (0.51-0.97)	0.94 (0.60-1.47)	0.90 (0.66-1.23)	0.99 (0.74-1.33)	0.89 (0.67-1.20)	0.82 (0.44-1.52)
Unadjusted	Winter 2020/21 (closed)	Women	21 - 30	Hospitalisation	0.77 (0.10-6.01)	1	1.21 (0.15-9.71)	-	-	1.46 (0.32-6.68)	-	-
Adjusted	Winter 2020/21 (closed)	Women	21 - 30	Hospitalisation	0.76 (0.09-6.09)	1	0.92 (0.11-7.87)	-	-	2.04 (0.42-9.80)	-	-
Unadjusted	Winter 2020/21 (closed)	Women	31 - 40	Any case	0.88 (0.55-1.42)	1	0.85 (0.58-1.26)	0.29 (0.09-0.92)	0.91 (0.70-1.17)	1.22 (0.98-1.54)	0.91 (0.71-1.15)	0.93 (0.62-1.40)
Adjusted	Winter 2020/21 (closed)	Women	31 - 40	Any case	0.77 (0.48-1.24)	1	0.77 (0.52-1.15)	0.28 (0.09-0.90)	0.93 (0.72-1.20)	1.27 (1.01-1.60)	0.97 (0.76-1.23)	1.00 (0.66-1.50)
Unadjusted	Winter 2020/21 (closed)	Women	31 - 40	Hospitalisation	1.23 (0.15-10.20)	1	1.90 (0.40-9.04)	-	0.88 (0.27-2.92)	0.47 (0.06-3.56)	1.57 (0.65-3.78)	-
Adjusted	Winter 2020/21 (closed)	Women	31 - 40	Hospitalisation	1.22 (0.14-10.34)	1	1.52 (0.30-7.66)	-	0.78 (0.23-2.66)	0.47 (0.06-3.62)	1.87 (0.76-4.62)	-
Unadjusted	Winter 2020/21 (closed)	Women	41 - 50	Any case	1.14 (0.70-1.86)	1	0.56 (0.33-0.95)	0.48 (0.17-1.33)	0.65 (0.47-0.91)	0.70 (0.48-1.01)	0.76 (0.57-1.02)	0.61 (0.38-0.99)
Adjusted	Winter 2020/21 (closed)	Women	41 - 50	Any case	0.91 (0.55-1.49)	1	0.51 (0.30-0.87)	0.42 (0.15-1.17)	0.68 (0.48-0.95)	0.70 (0.48-1.02)	0.78 (0.59-1.05)	0.63 (0.39-1.02)
Unadjusted	Winter 2020/21 (closed)	Women	41 - 50	Hospitalisation	1.24 (0.15-9.99)	1	1.36 (0.31-6.01)	1.26 (0.16-10.12)	0.77 (0.18-3.28)	-	0.55 (0.13-2.30)	0.68 (0.09-5.34)

Adjustment	Time period	Sex	Age group (years)	Outcome	Household member of primary teacher	Neither	Household member of secondary teacher	Household member other teacher	Nursery/Primary or Nursery	Primary	Secondary	Teacher in other sector
Adjusted	Winter 2020/21 (closed)	Women	41 - 50	Hospitalisation	1.39 (0.16-11.75)	1	1.50 (0.33-6.87)	1.30 (0.15-10.88)	1.32 (0.29-6.01)	-	0.73 (0.17-3.13)	0.76 (0.10-6.03)
Unadjusted	Winter 2020/21 (closed)	Women	51 - 65	Any case	1.04 (0.78-1.38)	1	0.75 (0.54-1.03)	1.09 (0.63-1.86)	0.72 (0.51-1.02)	0.90 (0.66-1.23)	0.63 (0.45-0.88)	0.65 (0.43-0.98)
Adjusted	Winter 2020/21 (closed)	Women	51 - 65	Any case	0.82 (0.61-1.09)	1	0.60 (0.43-0.83)	0.95 (0.55-1.64)	0.67 (0.47-0.96)	0.88 (0.64-1.21)	0.67 (0.48-0.94)	0.66 (0.43-1.00)
Unadjusted	Winter 2020/21 (closed)	Women	51 - 65	Hospitalisation	0.83 (0.33-2.08)	1	0.16 (0.02-1.13)	-	0.50 (0.12-2.07)	-	0.34 (0.08-1.41)	-
Adjusted	Winter 2020/21 (closed)	Women	51 - 65	Hospitalisation	0.71 (0.28-1.84)	1	0.13 (0.02-0.96)	-	0.55 (0.13-2.33)	-	0.52 (0.12-2.16)	-
Unadjusted	Spring term 2021 (phased)	Men	21 - 30	Any case	0.72 (0.43-1.20)	1	1.00 (0.59-1.72)	0.93 (0.37-2.35)	4.08 (1.02-16.32)	0.78 (0.10-6.09)	0.37 (0.09-1.51)	1.88 (0.22-16.14)
Adjusted	Spring term 2021 (phased)	Men	21 - 30	Any case	0.65 (0.39-1.10)	1	0.98 (0.57-1.68)	0.87 (0.34-2.20)	4.69 (1.15-19.02)	0.88 (0.11-6.94)	0.39 (0.09-1.61)	2.03 (0.24-17.52)
Unadjusted	Spring term 2021 (phased)	Men	21 - 30	Hospitalisation	-	1	-	-	-	-	-	-
Adjusted	Spring term 2021 (phased)	Men	21 - 30	Hospitalisation	-	1	-	-	-	-	-	-
Unadjusted	Spring term 2021 (phased)	Men	31 - 40	Any case	1.26 (0.88-1.82)	1	1.01 (0.60-1.71)	0.44 (0.14-1.40)	2.65 (1.06-6.61)	2.80 (1.02-7.66)	1.11 (0.61-2.03)	0.90 (0.21-3.83)
Adjusted	Spring term 2021 (phased)	Men	31 - 40	Any case	1.19 (0.82-1.73)	1	0.87 (0.51-1.48)	0.38 (0.12-1.23)	2.95 (1.17-7.44)	2.94 (1.06-8.15)	1.19 (0.64-2.20)	0.93 (0.21-4.04)

Adjustment	Time period	Sex	Age group (years)	Outcome	Household member of primary teacher	Neither	Household member of secondary teacher	Household member other teacher	Nursery/Primary or Nursery	Primary	Secondary	Teacher in other sector
Unadjusted	Spring term 2021 (phased)	Men	31 - 40	Hospitalisation	-	1	-	-	-	-	-	-
Adjusted	Spring term 2021 (phased)	Men	31 - 40	Hospitalisation	-	1	-	-	-	-	-	-
Unadjusted	Spring term 2021 (phased)	Men	41 - 50	Any case	2.23 (1.58-3.15)	1	0.85 (0.46-1.57)	1.67 (0.75-3.75)	2.00 (0.68-5.92)	2.65 (0.98-7.14)	1.24 (0.59-2.61)	0.55 (0.07-4.18)
Adjusted	Spring term 2021 (phased)	Men	41 - 50	Any case	1.93 (1.36-2.75)	1	0.70 (0.37-1.31)	1.25 (0.55-2.85)	2.24 (0.73-6.87)	2.87 (1.04-7.94)	1.10 (0.51-2.36)	0.43 (0.06-3.40)
Unadjusted	Spring term 2021 (phased)	Men	41 - 50	Hospitalisation	-	1	-	-	-	-	-	-
Adjusted	Spring term 2021 (phased)	Men	41 - 50	Hospitalisation	-	1	-	-	-	-	-	-
Unadjusted	Spring term 2021 (phased)	Men	51 - 65	Any case	0.93 (0.58-1.49)	1	0.84 (0.45-1.57)	0.48 (0.15-1.54)	2.40 (0.67-8.63)	2.81 (0.77-10.21)	0.85 (0.37-1.96)	1.03 (0.24-4.45)
Adjusted	Spring term 2021 (phased)	Men	51 - 65	Any case	0.75 (0.46-1.21)	1	0.67 (0.36-1.25)	0.44 (0.14-1.41)	2.39 (0.64-8.93)	2.92 (0.76-11.20)	0.82 (0.35-1.92)	1.10 (0.25-4.87)
Unadjusted	Spring term 2021 (phased)	Men	51 - 65	Hospitalisation	0.39 (0.05-2.89)	1	-	-	-	-	2.75 (0.55-13.74)	-
Adjusted	Spring term 2021 (phased)	Men	51 - 65	Hospitalisation	0.37 (0.05-2.82)	1	-	-	-	-	4.23 (0.82-21.85)	-
Unadjusted	Spring term 2021 (phased)	Women	21 - 30	Any case	0.52 (0.27-1.03)	1	0.78 (0.42-1.45)	0.62 (0.22-1.72)	1.65 (1.01-2.70)	2.08 (1.38-3.11)	1.06 (0.61-1.85)	2.22 (0.97-5.05)

Adjustment	Time period	Sex	Age group (years)	Outcome	Household member of primary teacher	Neither	Household member of secondary teacher	Household member other teacher	Nursery/Primary or Nursery	Primary	Secondary	Teacher in other sector
Adjusted	Spring term 2021 (phased)	Women	21 - 30	Any case	0.49 (0.25-0.98)	1	0.80 (0.43-1.49)	0.54 (0.19-1.50)	1.75 (1.06-2.90)	2.29 (1.51-3.46)	1.27 (0.72-2.23)	2.46 (1.05-5.72)
Unadjusted	Spring term 2021 (phased)	Women	21 - 30	Hospitalisation	-	1	-	-	-	-	-	-
Adjusted	Spring term 2021 (phased)	Women	21 - 30	Hospitalisation	-	1	-	-	-	-	-	-
Unadjusted	Spring term 2021 (phased)	Women	31 - 40	Any case	1.63 (0.73-3.67)	1	1.00 (0.50-1.99)	0.31 (0.04-2.30)	1.62 (1.12-2.33)	2.53 (1.86-3.44)	0.72 (0.45-1.15)	0.84 (0.42-1.66)
Adjusted	Spring term 2021 (phased)	Women	31 - 40	Any case	1.37 (0.60-3.13)	1	0.87 (0.43-1.76)	0.23 (0.03-1.70)	1.89 (1.29-2.77)	2.85 (2.06-3.94)	0.72 (0.44-1.19)	0.87 (0.43-1.75)
Unadjusted	Spring term 2021 (phased)	Women	31 - 40	Hospitalisation	-	1	-	-	3.65 (1.08-12.37)	0.83 (0.11-6.50)	-	-
Adjusted	Spring term 2021 (phased)	Women	31 - 40	Hospitalisation	-	1	-	-	7.11 (1.71-29.54)	0.85 (0.10-6.97)	-	-
Unadjusted	Spring term 2021 (phased)	Women	41 - 50	Any case	1.38 (0.54-3.54)	1	0.76 (0.30-1.89)	0.93 (0.22-3.96)	2.65 (1.81-3.89)	2.09 (1.39-3.14)	0.52 (0.28-1.00)	1.42 (0.77-2.63)
Adjusted	Spring term 2021 (phased)	Women	41 - 50	Any case	1.07 (0.41-2.79)	1	0.58 (0.23-1.47)	0.88 (0.20-3.81)	2.72 (1.83-4.04)	2.36 (1.55-3.60)	0.55 (0.28-1.05)	1.40 (0.74-2.63)
Unadjusted	Spring term 2021 (phased)	Women	41 - 50	Hospitalisation	12.17 (0.72-206.95)	1	-	-	-	-	0.79 (0.10-6.11)	-
Adjusted	Spring term 2021 (phased)	Women	41 - 50	Hospitalisation	11.73 (0.71-194.80)	1	-	-	-	-	0.64 (0.08-5.29)	-

Adjustment	Time period	Sex	Age group (years)	Outcome	Household member of primary teacher	Neither	Household member of secondary teacher	Household member other teacher	Nursery/Primary or Nursery	Primary	Secondary	Teacher in other sector
Unadjusted	Spring term 2021 (phased)	Women	51 - 65	Any case	1.13 (0.58-2.20)	1	0.74 (0.36-1.54)	2.81 (1.11-7.10)	2.02 (1.15-3.56)	2.22 (1.34-3.69)	0.61 (0.28-1.30)	1.10 (0.50-2.43)
Adjusted	Spring term 2021 (phased)	Women	51 - 65	Any case	0.75 (0.38-1.48)	1	0.62 (0.30-1.28)	2.20 (0.84-5.75)	1.99 (1.12-3.54)	2.21 (1.31-3.71)	0.67 (0.31-1.46)	1.16 (0.52-2.59)
Unadjusted	Spring term 2021 (phased)	Women	51 - 65	Hospitalisation	2.04 (0.44-9.52)	1	-	-	1.41 (0.17-11.75)	-	0.48 (0.06-3.62)	2.37 (0.26-21.23)
Adjusted	Spring term 2021 (phased)	Women	51 - 65	Hospitalisation	1.90 (0.39-9.37)	1	-	-	1.59 (0.19-13.47)	-	0.46 (0.06-3.59)	2.57 (0.28-23.84)
Unadjusted	Summer term 2021 (open)	Men	21 - 30	Any case	1.55 (1.32-1.81)	1	1.08 (0.87-1.34)	1.21 (0.89-1.64)	1.49 (0.73-3.01)	1.94 (1.06-3.53)	1.64 (1.16-2.32)	1.89 (0.72-4.99)
Adjusted	Summer term 2021 (open)	Men	21 - 30	Any case	1.34 (1.14-1.57)	1	0.93 (0.75-1.15)	1.07 (0.79-1.45)	1.36 (0.67-2.76)	1.78 (0.97-3.26)	1.48 (1.04-2.12)	2.03 (0.77-5.39)
Unadjusted	Summer term 2021 (open)	Men	21 - 30	Hospitalisation	-	1	-	-	-	-	-	-
Adjusted	Summer term 2021 (open)	Men	21 - 30	Hospitalisation	-	1	-	-	-	-	-	-
Unadjusted	Summer term 2021 (open)	Men	31 - 40	Any case	1.54 (1.24-1.92)	1	1.16 (0.85-1.58)	0.94 (0.57-1.56)	1.26 (0.62-2.54)	2.80 (1.51-5.22)	2.04 (1.51-2.75)	1.06 (0.48-2.32)
Adjusted	Summer term 2021 (open)	Men	31 - 40	Any case	1.38 (1.11-1.73)	1	1.06 (0.78-1.45)	0.94 (0.57-1.55)	1.11 (0.53-2.34)	2.82 (1.51-5.27)	1.89 (1.39-2.57)	0.99 (0.45-2.18)
Unadjusted	Summer term 2021 (open)	Men	31 - 40	Hospitalisation	-	1	0.84 (0.10-6.74)	-	-	4.66 (0.42-51.41)	1.22 (0.14-10.44)	-

Adjustment	Time period	Sex	Age group (years)	Outcome	Household member of primary teacher	Neither	Household member of secondary teacher	Household member other teacher	Nursery/Primary or Nursery	Primary	Secondary	Teacher in other sector
Adjusted	Summer term 2021 (open)	Men	31 - 40	Hospitalisation	-	1	0.72 (0.09-5.91)	-	-	3.80 (0.32-45.78)	1.55 (0.17-14.04)	-
Unadjusted	Summer term 2021 (open)	Men	41 - 50	Any case	1.53 (1.18-1.98)	1	1.24 (0.88-1.75)	1.74 (1.10-2.75)	2.07 (0.91-4.70)	3.94 (2.06-7.55)	1.88 (1.31-2.70)	1.39 (0.54-3.55)
Adjusted	Summer term 2021 (open)	Men	41 - 50	Any case	1.13 (0.87-1.48)	1	1.01 (0.71-1.44)	1.44 (0.90-2.31)	1.82 (0.78-4.23)	3.52 (1.81-6.86)	1.65 (1.13-2.39)	1.30 (0.50-3.40)
Unadjusted	Summer term 2021 (open)	Men	41 - 50	Hospitalisation	-	1	-	-	8.37 (0.52-135.24)	-	1.03 (0.13-8.16)	-
Adjusted	Summer term 2021 (open)	Men	41 - 50	Hospitalisation	-	1	-	-	8.14 (0.45-148.81)	-	1.20 (0.14-10.22)	-
Unadjusted	Summer term 2021 (open)	Men	51 - 65	Any case	1.48 (1.10-1.99)	1	1.31 (0.88-1.95)	1.47 (0.85-2.53)	2.24 (0.63-7.96)	1.94 (0.55-6.77)	2.09 (1.36-3.20)	1.15 (0.45-2.92)
Adjusted	Summer term 2021 (open)	Men	51 - 65	Any case	1.12 (0.82-1.51)	1	0.99 (0.66-1.48)	1.10 (0.63-1.93)	2.49 (0.69-9.06)	1.85 (0.51-6.74)	1.74 (1.12-2.69)	1.04 (0.40-2.68)
Unadjusted	Summer term 2021 (open)	Men	51 - 65	Hospitalisation	-	1	-	-	-	-	-	-
Adjusted	Summer term 2021 (open)	Men	51 - 65	Hospitalisation	-	1	-	-	-	-	-	-
Unadjusted	Summer term 2021 (open)	Women	21 - 30	Any case	1.40 (1.10-1.76)	1	1.10 (0.83-1.45)	1.06 (0.71-1.59)	2.15 (1.68-2.74)	1.82 (1.43-2.33)	1.95 (1.55-2.44)	1.25 (0.68-2.29)
Adjusted	Summer term 2021 (open)	Women	21 - 30	Any case	1.26 (0.99-1.59)	1	1.01 (0.76-1.33)	0.96 (0.64-1.43)	2.07 (1.62-2.65)	1.82 (1.42-2.32)	1.91 (1.51-2.40)	1.26 (0.69-2.32)

Adjustment	Time period	Sex	Age group (years)	Outcome	Household member of primary teacher	Neither	Household member of secondary teacher	Household member other teacher	Nursery/Primary or Nursery	Primary	Secondary	Teacher in other sector
Unadjusted	Summer term 2021 (open)	Women	21 - 30	Hospitalisation	0.81 (0.10-6.36)	1	-	-	-	1.15 (0.14-9.31)	1.52 (0.32-7.12)	-
Adjusted	Summer term 2021 (open)	Women	21 - 30	Hospitalisation	1.27 (0.16-10.22)	1	-	-	-	1.66 (0.19-14.36)	2.02 (0.41-10.00)	-
Unadjusted	Summer term 2021 (open)	Women	31 - 40	Any case	1.07 (0.62-1.88)	1	0.90 (0.55-1.49)	0.32 (0.08-1.32)	1.74 (1.37-2.22)	2.33 (1.85-2.92)	1.26 (0.99-1.62)	1.23 (0.81-1.85)
Adjusted	Summer term 2021 (open)	Women	31 - 40	Any case	0.92 (0.52-1.61)	1	0.75 (0.45-1.24)	0.29 (0.07-1.20)	1.85 (1.44-2.37)	2.57 (2.03-3.25)	1.41 (1.09-1.82)	1.25 (0.82-1.90)
Unadjusted	Summer term 2021 (open)	Women	31 - 40	Hospitalisation	-	1	-	-	1.19 (0.35-4.00)	-	0.66 (0.16-2.81)	1.77 (0.39-8.12)
Adjusted	Summer term 2021 (open)	Women	31 - 40	Hospitalisation	-	1	-	-	1.05 (0.31-3.62)	-	0.80 (0.18-3.44)	1.73 (0.36-8.36)
Unadjusted	Summer term 2021 (open)	Women	41 - 50	Any case	1.51 (0.81-2.79)	1	0.69 (0.35-1.37)	0.83 (0.25-2.71)	1.63 (1.23-2.17)	2.00 (1.49-2.69)	1.51 (1.15-1.99)	1.40 (0.93-2.10)
Adjusted	Summer term 2021 (open)	Women	41 - 50	Any case	1.26 (0.67-2.37)	1	0.56 (0.28-1.12)	0.69 (0.21-2.31)	1.45 (1.08-1.95)	1.85 (1.36-2.51)	1.53 (1.15-2.04)	1.36 (0.89-2.07)
Unadjusted	Summer term 2021 (open)	Women	41 - 50	Hospitalisation	-	1	-	-	0.58 (0.08-4.47)	-	1.39 (0.30-6.31)	-
Adjusted	Summer term 2021 (open)	Women	41 - 50	Hospitalisation	-	1	-	-	0.86 (0.11-6.76)	-	1.91 (0.39-9.34)	-

Adjustment	Time period	Sex	Age group (years)	Outcome	Household member of primary teacher	Neither	Household member of secondary teacher	Household member other teacher	Nursery/Primary or Nursery	Primary	Secondary	Teacher in other sector
Unadjusted	Summer term 2021 (open)	Women	51 - 65	Any case	1.43 (0.90-2.25)	1	1.55 (1.02-2.36)	1.39 (0.49-3.96)	2.47 (1.70-3.57)	2.34 (1.60-3.43)	1.42 (0.97-2.07)	0.86 (0.45-1.64)
Adjusted	Summer term 2021 (open)	Women	51 - 65	Any case	1.01 (0.64-1.61)	1	1.10 (0.72-1.68)	1.09 (0.38-3.15)	2.01 (1.37-2.94)	2.03 (1.37-3.01)	1.42 (0.96-2.10)	0.81 (0.42-1.56)
Unadjusted	Summer term 2021 (open)	Women	51 - 65	Hospitalisation	-	1	-	1.90 (0.21-17.09)	1.22 (0.15-9.97)	-	0.42 (0.06-3.22)	-
Adjusted	Summer term 2021 (open)	Women	51 - 65	Hospitalisation	-	1	-	2.60 (0.26-25.92)	1.07 (0.13-9.12)	-	0.51 (0.06-4.11)	-

Table S4 Comparison of rate ratios for any case, hospitalisation with COVID-19 and severe COVID-19 for autumn 2020 (open) and summer 2021 (open) versus spring/summer 2020 (closed)

**Outcome**	**Adjustment**	**Period**	**Teacher**	**Household member of teacher**	**Healthcare worker, patient facing**	**Household member of healthcare worker**	**Healthcare worker, non-patient facing**
Any case	Unadjusted	Re-opened 2021	4.33 (3.39-5.55)	1.68 (1.40-2.02)	0.06 (0.05-0.06)	0.37 (0.33-0.42)	0.64 (0.34-1.20)
Any case	Adjusted	Re-opened 2021	3.95 (3.08-5.07)	1.43 (1.18-1.73)	0.06 (0.05-0.06)	0.33 (0.30-0.38)	0.70 (0.37-1.33)
Any case	Unadjusted	Re-opened 2020	3.64 (2.85-4.64)	1.55 (1.29-1.86)	0.26 (0.25-0.28)	0.51 (0.46-0.57)	0.80 (0.45-1.44)
Any case	Adjusted	Re-opened 2020	3.46 (2.70-4.43)	1.36 (1.13-1.64)	0.26 (0.24-0.28)	0.47 (0.42-0.52)	0.87 (0.48-1.56)
Hospitalisation	Unadjusted	Re-opened 2021	1.73 (0.88-3.42)	0.57 (0.25-1.30)	0.16 (0.10-0.25)	0.41 (0.24-0.67)	-
Hospitalisation	Adjusted	Re-opened 2021	1.69 (0.85-3.38)	0.53 (0.23-1.20)	0.14 (0.09-0.23)	0.36 (0.21-0.61)	-
Hospitalisation	Unadjusted	Re-opened 2020	2.46 (1.38-4.40)	1.55 (0.95-2.53)	0.56 (0.44-0.72)	0.62 (0.44-0.86)	1.64 (0.37-7.27)
Hospitalisation	Adjusted	Re-opened 2020	2.37 (1.31-4.28)	1.47 (0.88-2.44)	0.54 (0.42-0.70)	0.57 (0.40-0.80)	1.93 (0.42-8.77)
Severe	Unadjusted	Re-opened 2021	0.45 (0.05-4.12)	-	0.10 (0.01-0.77)	0.87 (0.30-2.49)	-
Severe	Adjusted	Re-opened 2021	0.44 (0.05-4.15)	-	0.08 (0.01-0.64)	0.89 (0.30-2.67)	-
Severe	Unadjusted	Re-opened 2020	0.87 (0.20-3.84)	1.00 (0.38-2.65)	0.82 (0.45-1.49)	1.11 (0.61-2.03)	-
Severe	Adjusted	Re-opened 2020	0.59 (0.13-2.80)	0.97 (0.35-2.71)	0.87 (0.46-1.63)	1.18 (0.62-2.27)	-

Table of abbreviations

Abbreviation	Full text	Explanation
CHI	Community Health Index	Unique, individual health care identifier used across health care contacts in Scotland
SIMD	Scottish Index of Multiple Deprivation	Area-based measure of deprivation
SMR	Scottish Morbidity Records	Healthcare utilisation records
PIS	Prescribing Information System	Holds data relating to all prescriptions dispensed in Scotland
HSC-PBPP	Health and Social Care Public Benefit and Privacy Panel	Provides scrutiny and approval to ensure maintenance of information governance principles in the use of public data
DPIA	Data Protection Impact Assessment	
GTCS	General Teaching Council for Scotland	Maintains the professional register for teachers in Scotland
P1 to P7	Primary 1 to 7	Name applied to each year of education from age 5-12
S1 to S6	Secondary 1 to 6	Name applied to each year of education from age 13-18
CERG	COVID-19 Education Recovery Group	Scottish Government convened multi-agency group for management of COVID-19 in education
GPCD	General Practitioner Contractor Database	Database of General Practitioners in Scotland
SWISS	Scottish Workforce Information Standard System	Database of NHS staff in Scotland
UPRN	Unique Property Reference Number	
ICU	Intensive Care Unit	
RR	Risk ratio	
CI	Confidence interval	95% confidence intervals are reported throughout
OR	Odds ratio	
ONS	Office for National Statistics	
PHS	Public Health Scotland	
ECDC	European Centre for Disease Control	
